# Emergent coexistence and the limits of reductionism in ecological communities

**DOI:** 10.1371/journal.pcbi.1014116

**Published:** 2026-03-27

**Authors:** Guim Aguadé-Gorgorió, Sonia Kéfi

**Affiliations:** 1 Institut des Sciences de l’Évolution de Montpellier, University of Montpellier, Montpellier, France; 2 Santa Fe Institute, Santa Fe, New Mexico, United States of America; University of Padua: Universita degli Studi di Padova, ITALY

## Abstract

Understanding the extent to which the species composition of a community can be explained by pairwise interactions is a long-standing question in ecology. Recent observations have revealed that stable microbial communities contain a high number of species that cannot coexist in pairs, providing new empirical elements to explore this question from a fresh perspective. Here, using species-rich models of ecological communities with pairwise interactions alone, we show that emergent coexistence arises naturally in an extent that is consistent with empirical observations. Interestingly, this phenomenon does not require additional mechanisms like intransitive or higher-order interactions; rather, coexistence can arise from the dense networks of indirect effects. As diversity increases, we show that indirect effects can become so intricate that pairwise interactions decouple from community composition, revealing a fundamental limit to reductionist explanations of coexistence. Our findings provide the theoretical foundations to understand how simple pairwise interactions can lead to emergent coexistence patterns in ecological communities.

## Author summary

Ecological communities often contain species that cannot coexist by pairs, yet thrive together in the full community. This observation of Emergent Coexistence (EC) raises a fundamental question: how much can we really learn about a community by studying pairwise interactions between its constituent species isolated from the community? Using mathematical models of species-rich communities, we show that EC is a common outcome when many species interact by pairs, and does not require higher-order nor rock-paper-scissors interactions. In these communities, long chains of indirect effects–where one species influences another through intermediaries–can accumulate and reshape coexistence patterns, to the point where pairwise relations increasingly fail to predict which species will persist in the full community. Our work provides a theoretical foundation to understand when pairwise measurements can inform about species coexistence in complex ecological communities.

*In this great chain of causes and effects, no single fact can be considered in isolation*.

- Alexander Von Humboldt

## Introduction

Ecological systems are inherently complex, because of the number of constituent species and the diverse ways in which they depend on each other. Typically, ecological communities are conceptualized as a set of species connected by a network of pairwise interactions [1]. Despite knowing that additional mechanisms other than pairwise interactions may be in place [2], this framework continues to dominate how ecological communities are conceptualized and studied.

**Fig 1:**
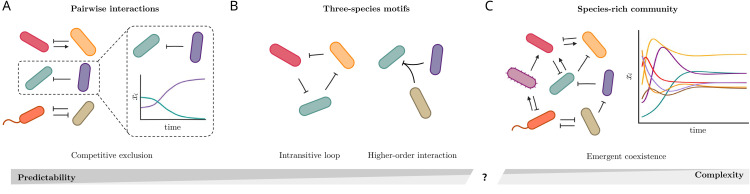
Can we predict community composition from pairwise interactions?. **(A)** Experimental studies of microbial communities are able to grow species by pairs and infer if they coexist or exclude one another (dashed box, [11]). **(B)** In three-species models, competitive exclusion can be avoided, e.g., by intransitive competition motifs such as rock-paper-scissors loops, or by higher-order interactions [22]. **(C)** Yet, species-rich microbial communities can coexist and harbor many excluding pairs and no rock-paper-scissors loops, hinting to the possibility that coexistence is an emergent, community-level property [11].

However, a long-standing problem in ecology is the extent to which pairwise interactions alone are sufficient to understand and predict community properties such as coexistence and stability. This naturally leads to a fundamental question: can knowledge of pairwise interactions reliably predict which species combinations will form a stable community, especially so as diversity increases (Fig 1)? Specifically, how much does pairwise coexistence translate to coexistence in larger communities? Are species pairs that exhibit competitive exclusion inherently incompatible? Coherence between pairwise and community-level coexistence would support reductionist views of community assembly, which could be leveraged to synthetically assemble species consortia in the lab [3,4]. Yet, lessons learned from complex systems indicate that predicting the behavior of large ensembles from information of system subsets is not always possible [5,6]. In ecology, quantifying the extent to which multispecies coexistence falls within the domains of reductionism or emergence remains an open task.

Experiments with microbial communities have recently shed a new light on this question. Microbial ecosystems indeed offer a powerful experimental platform for investigating how species interactions govern fundamental community properties [3,7–10]. Their unique tractability enables researchers to perform controlled co-culture experiments, allowing the inference of pairwise species relationships [4,11,12] (Fig 1). A recent experimental observation suggests that stable microbial communities often contain many species pairs that, when isolated, fail to coexist due to strong competition [4,11–13] (Fig 1A). This phenomenon, in which community composition seems to contradict expectations from pairwise interactions, has recently been labeled as *Emergent Coexistence* (EC, [11], Fig 1C). While cooperative interactions are common in microbial communities [14], the apparent incompatibility of many of their constituent species raises questions about the mechanisms that support stable coexistence in those communities. This apparent paradox suggests that pairwise interactions alone may be insufficient to predict species coexistence, pointing towards more complex, community-level mechanisms, as similarly discussed for plant communities [15,16].

Mathematical models have long been a cornerstone in exploring how species interactions shape their coexistence [17,18]. Many mechanisms have been proposed to circumvent the problem of competitive exclusion, including positive interactions [19], non-random network structures [20] or the role of space in reducing effective competition [18]. Modern coexistence theory further formalizes these ideas by distinguishing between stabilizing mechanisms, which promote niche differentiation, and equalizing mechanisms, which reduce fitness differences among species [18]. Despite these strong theoretical foundations for multispecies coexistence, a mathematical framework quantifying if and how information from pairwise interactions predicts species-rich community composition is still lacking [4,11,21].

In laboratory observations of EC in microbial communities, pairwise competition is strong and pervasive, and space does not seem to play a major role [4,11–13]. In this context, typical explanations for multispecies coexistence involve higher-order interactions or intransitive competition [1,23] (Fig 1B). Higher-order interactions emerge when a third species can modulate the interaction strength between the first two species [2,23]. Intransitive competition is characterized by a lack of competitive hierarchy, for example in a rock-paper-scissors loop (Fig 1B, [24]). Both of these mechanisms could buffer pairwise exclusions through the presence of a third species. However, their empirical prevalence and explanatory power in natural and laboratory ecosystems remain unclear [1,25,26]. Moreover, EC has also been observed without intransitive loops [11,13].

In the absence of higher-order or intransitive mechanisms, a species-rich community is still pervaded by indirect effects, by which a species’ growth is influenced by chains of cumulative effects through other species (Fig 1C, [22,27,28]). These indirect effects, exemplified by the principle “the enemy of my enemy is my friend”, have been extensively studied in small-scale interaction motifs as a mechanism of coexistence in the presence of strong competition [22,27,29] (Fig 1B). However, as community complexity increases, quantifying the magnitude and significance of these indirect effects in both theoretical models and empirical systems remains a formidable challenge [30–32].

Here, we study if EC is possible in species-rich models of random pairwise interactions due to indirect effects alone, or else if additional mechanisms need to be invoked. We explore EC in the generalized Lotka-Volterra (GLV) model, a simple phenomenological description of a community of many interacting species [9,10,12,33], as well as in other dynamical models (described in S1 Text I.G). Dynamical models based on pairwise interactions between species are an idealized coarse-graining of more complex ecological mechanisms involving consumer-resource dynamics, environmental coupling or metabolite cross-feeding [34,35]. By assuming this phenomenological approximation, in which we lump all processes into effective interaction strengths between species, our main goal is to provide a first understanding of how pairwise interactions shape –and the extent to which they allow us to predict– the emergent species composition of a community. The GLV model describes the abundance *N*_*i*_ of a set of *S* species that follow:


$$\frac{dN_{i}}{dt}=r_{i}N_{i}\left(1-\frac{N_{i}+\sum_{j\neq i}^{S}a_{ij}N_{j}}{K_{i}}\right),$$ (1)

where *r*_*i*_ are the intrinsic growth rates, *K*_*i*_ the carrying capacities and *a*_*ij*_ the effects of each other species *j* on the growth of species *i*. A common simplification is to write the model in terms of the relative yield of species to their carrying capacity, $x_{i}=N_{i}/K_{i}$ [36], and define interaction strengths relative to self-regulation ($A_{ij}=a_{ij}K_{j}/K_{i}$; [32,37–39], see Materials and Methods (MM)). In this setting, the dynamics of species *i* in the GLV model follow:


$$\frac{dx_{i}}{dt}=r_{i}x_{i}\left(1-x_{i}+\sum_{j\neq i}^{S}A_{ij}x_{j}\right).$$ (2)

The growth of species *i* is modulated by linear replication, quadratic self-regulation and the interaction with each of the other species, mediated by *A*_*ij*_. From an empirical perspective, inferring all the S(S−1) interactions of *A* remains a formidable and often ill-posed challenge [12,40,41]: even if the GLV model was the true generative process of the studied ecological dynamics, accumulated research indicates that multiple *A* matrices can simultaneously provide a good fit to the same temporal datasets, yielding apparently excellent but non-identifiable fits [42–45]. Instead, a common procedure to study the properties of (2) without the need to infer the exact values of *A* is to assume that we can, at least, infer the statistical properties of interactions such as the interaction strength (μ), heterogeneity (σ) and network connectivity (*C*), and sample random interaction matrices based on those statistics [33,46–48].

While interaction networks of macro-organisms such as food webs or plant-pollinator systems are typically highly structured [20], empirical characterizations of microbial interaction matrices suggest that the random-interaction assumption is a reasonable first approximation for describing their aggregate statistical properties [10,39,49,50]. Experimental inference of microbial interaction networks consistently reveals broad and heterogeneous distributions of interaction coefficients centered near zero, often with both positive and negative links and a variable connectivity [12,51]. Interaction matrices in small microbial consortia assembled in the laboratory are often dense (C≈0.7−0.9), likely due to limited resource complexity [12,26,52], whereas empirical matrices for larger communities are typically much sparser, with only a small fraction of all possible pairwise interactions exerting a measurable effect [41,49].

Across all these systems characterized by different interaction properties, and starting from a large pool of *S* randomly interacting species, are there subsets of species that can coexist stably even if some of their constituent species do not coexist in pairs (Fig 1)? Here, we study whether this is possible, the mechanisms behind it, and the consequences for our understanding of multispecies coexistence.

## Results

### Emergent coexistence is common in models with random pairwise interactions

We investigate the possibility of stable communities with EC in the GLV model (Eq. 2). We start from a pool of *S* species and random interaction coefficients *A*_*ij*_ defined by μ and σ, and study if the system reaches a stable state, in which $S^{*}\leq S$ species coexist with interaction coefficients A^*^, where at least one pair of the coexisting species would not coexist outside of the community due to competitive exclusion. Species *i* and *j* are considered an excluding pair and would not coexist in isolation if either $A_{ij}^{*}<-1$, $A_{ji}^{*}<-1$ or both. We call such a stable state that contains at least one exclusionary pair an *EC state*. Note that EC states require, by definition, coexistence between at least three species: a stable state with one survivor has no interactions, and a stable state with two survivors requires that their interaction is not exclusionary.

**Fig 2:**
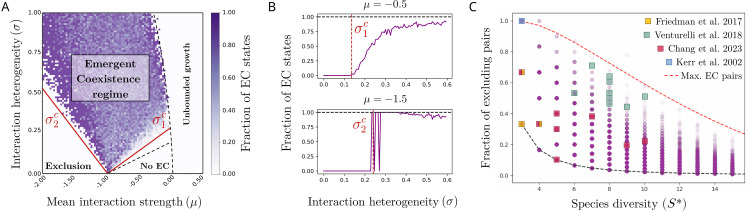
Emergent coexistence in models of pairwise interactions. The GLV model has a rich parameter space with four dynamical regimes depending on the values of the mean interaction strength (μ) and standard deviation (σ) [33,39,53]. Note that in some GLV model studies [33], μ and σ are rescaled with pool diversity *S*, which implies a focus on the weak interactions domain. Other studies, such as here and [39,54], do not rescale interactions with diversity; this allows for stronger competition and leads to the full parameter space as presented here. **(A)** Simulations of the GLV model starting from a pool of *S*=80 species and interactions randomly sampled from a normal distribution (see Materials and Methods). For each, we simulate 100 systems, each with a different *A* matrix and different random initial conditions, and divide the number of final stable states that harbor at least one excluding pair (EC states) against the number of stable states that contain at least three coexisting species, with or without EC. Most stable communities harbor EC within an area delimited by two analytically estimated boundaries ($\sigma_{1}^{c}$, $\sigma_{2}^{c}$, red lines, see MM). For random and uncorrelated interactions, the EC regime is found inside the multistability regime, one of the four dynamical regimes of the GLV model (delimited by dashed lines, [54]). **(B)** Vertical slices of (A) at μ=−0.5 and μ=−1.5 and increasing σ, showing the transitions towards the regime where EC is prevalent. **(C)** For each community with EC found in **(A)**, we measure the final diversity S^*^ and the fraction of excluding pairs. Simulated communities (purple circles) and empirical data ([4,11,12,24], colored squares) are consistent with the predicted minimum fraction (dark dashed line, one pair over all possible pairs) and maximum fraction of excluding pairs (red dashed line, obtained from predicting the maximum possible diversity and fraction of excluding interactions given (μ,σ), see MM and S1 Text **II.**B.2) for the mathematical details.

To explore the presence of EC states in the GLV model numerically, we define a range of possible mean (μ) and heterogeneity (σ) of interactions between species (Fig 2A). For each pair of μ and σ values, we sample 100 systems, each with a different interaction matrix *A* and random initial conditions *x*_*i*_(*t* = 0). We integrate the GLV dynamics for these 100 systems, and we divide the number of stable states that contain EC against the number of stable s*t*ates that contain at least three coexisting species, with or without EC (Fig 2A). This provides a proxy for how common or rare it is to find stable communities that are EC states: if we compare the number of EC states against all possible stable states without the three-species requirement, we observe the same results with a region where EC fractions are very low, indicating not that EC states are very rare, but that we are sampling μ,σ values where coexistence of more than two species is very rare (Fig A in S1 Text). To focus on the role played by interspecies interactions, we present in the main text the results with homogeneous and normalized growth rates (*r*_*i*_ = *r* = 1) and carrying capacities (*K*_*i*_ = *K* = 1), and set network connectivity equal to 1 (*C* = 1). We describe in detail in S1 Text II.A,B,J how assuming heterogeneous growth rates, carrying capacities, and reduced connectivity yields qualitatively similar results to those presented here.

In all cases, results reveal that EC is a common outcome in the GLV model, provided that pairwise interactions are competitive and sufficiently heterogeneous (Fig 2B). If heterogeneity is low (σ≈0), EC is still possible provided that mean interaction strength is close to self-regulation (μ≈−1). In this large domain of the parameter space, a large fraction of stable communities harbor species pairs that cannot coexist in isolation (Fig 2A and 2B). We find analytical lower limits for the boundaries of this EC regime ($\sigma_{1}^{c}$ and $\sigma_{2}^{c}$ in Fig 2A), by considering that, for EC to be observed in the final community, a necessary –albeit not sufficient– condition is that exclusionary interactions and coexisting pairs need to be present in the initial species pool (see MM as well as S1 Text II.A for a step-by-step derivation). Notably, most communities with EC in the assumption of random and uncorrelated interactions are found inside a regime of the GLV model characterized by multiple stable states [33,53]. If interactions are not uncorrelated, but purely anti-symmetrical, the GLV model does not harbor multistability, and EC can happen inside the unique fixed point regime [33]. If interactions are purely symmetrical, we show mathematically in S1 Text II.A that stable communities cannot harbor EC (see [21] for a similar discussion on interaction symmetry and growth-competition trade-offs). The presence of multistability implies that different initial conditions, yet with the same species pool *S* and interactions *A*, can lead to different subsets of surviving species [54]. This is consistent with empirical observations, that have found EC in systems where varying the initial species abundances leads to different stable species compositions [11,55]. This highlights a possible, yet under-explored, link between interaction heterogeneity, EC and community multistability.

By replicating the study of Fig 2A with different models, we find that EC is still a prevalent outcome when growth rates and carrying capacities are heterogeneous and when interactions are sparse (see MM and Fig D, Fig F and Fig S in S1 Text). Additionally, the same qualitative results hold beyond the assumption of fully random interactions, when *A*_*ij*_ have a predator-prey structure [56], are highly skewed [41,49,57], are row- and column-correlated due to heterogeneous carrying capacities [38] or have nested or single-resource structures (Fig D in S1 Text). Finally, we show that stable communities with EC are also present in non-GLV models with Allee effects, multilayer interactions, saturating responses or sublinear growth ([53,58], Fig E in S1 Text), while recent work has found EC across a family of models with growth-competition and competition-colonization trade-offs [21]. Taken together, these results suggest that EC is not particular to the GLV model or the random interaction assumption, but rather is a general outcome in species-rich models once many interactions are competitive and heterogeneous. Our analysis so far only detects states that have *at least* one excluding pair, treating EC as a binary property. Following recent experimental [11] and numerical [21] results, a follow up question is to study the fraction of excluding pairs and hence the extent of EC that a single community can sustain.

### Analytical predictions match observations of the fraction of excluding pairs

A striking observation of EC experiments is that as many as 60∼70% of species pairs in a stable community do not coexist in co-culture [11,12]. Here, for each community found in the (μ,σ) range of Fig 2A with at least one excluding pair, we measure the fraction of species pairs that interact via competitive exclusion and hence would not coexist if isolated (Fig 2C, MM). We find that communities can sustain a highly variable fraction of excluding pairs, with the maximum fraction of such pairs decaying with species diversity (Fig 2C). The decrease of strongly competitive pairs is consistent with classical results from random matrix theory and the GLV model, for which larger communities become unstable if competition strength overcomes given limits [33,46]. Interestingly, Fig 2C conveys the intuition that EC is intrinsically a phenomenon of intermediate diversity, that falls between the common modeling extremes of either very small or large-*S* limits and would be hard to capture by either limiting perspective [53,59].

To estimate the maximum competition, and hence the maximum fraction of excluding pairs that communities with EC can sustain before becoming unstable, we use recent corrections of May’s limit for communities of moderate size. This allows us to predict the maximum diversity and fraction of exclusionary interactions for a community given (μ,σ) (Fig 2C red dashed line, see MM for an explanation of the method and S1 Text II.B.2 for the complete mathematical development, [54].) Additionally, the minimal fraction of excluding pairs in EC communities is simply one excluding pair over the total number of species pairs in a community of size S^*^, $S^{*}(S^{*}-1)/2$ (Fig 2C, dark dashed line). It is interesting to recall that these two bounds are qualitatively different: the lower bound reflects the presence of at least one exclusionary pair and is generically attained for all EC states. In comparison, the higher bound reflects the maximum fraction of excluding pairs that specific systems with random pairwise interactions and GLV dynamics can attain. In both cases, the bounds on the fraction of excluding pairs are consistent with experimental data [4,11,12], and confirm that the fraction of excluding pairs can be very large in communities of moderate size: a community of 10 species could sustain as many as 60% of species pairs that do not coexist in co-culture.

The qualitative trends observed in Fig 2C remain robust under heterogeneous carrying capacities or nested or hierarchical interactions (Fig F in S1 Text), as well as when incorporating growth rate heterogeneity or matrix sparsity, yet with small quantitative variations: increasing growth rate heterogeneity reduces the likelihood of observing large stable communities that sustain many excluding pairs. In contrast, greater network sparsity weakens overall competition, making diverse communities with many excluding pairs more likely (Fig G in S1 Text). Finally, recent work has found that larger fractions of excluding pairs can coexist if carrying capacities are sampled to ensure species coexistence [21]. In sum, we hypothesize that finding more excluding pairs in larger communities requires the presence of non-random interaction structures that can further enhance species coexistence. Given the results shown in Fig 2, a natural question to ask is how all these species can coexist if so many interactions are strongly competitive and there are no higher-order effects.

### Multispecies coexistence decouples from pairwise interactions at high collectivity

Early work on three-species models showed that positive indirect effects of length two, as in “the enemy of my enemy is my friend”, could allow competitors to coexist [22,29]. Yet, in networks of many interacting species and long chains of indirect effects, it becomes hard to determine who is friend and who is enemy. Importantly, recent work has shed new light to our understanding of indirect effects in species-rich communities [32]. Given a pool of *S* species interacting through matrix *A*, equilibrium states of the GLV model will be those in which at least a subset of S^*^ species with interactions encoded in A^*^ coexist with positive abundances. This translates to finding a subset of species with positive abundances by solving:


$$x^{*}=(I-A^{*}{)}^{-1}1.$$ (3)

Here the *i*,*j* element of the inverse matrix, $(I-A^{*}{)}_{ij}^{-1}$, encodes the net effect of species *j* on the equilibrium abundance of species *i* [29–32] (see S1 Text I.B for a detailed step by step development). The link between direct and net effects can be better understood by using the Neumann series [32]:


$$(I-A^{*}{)}^{-1}=I+A^{*}+(A^{*}{)}^{2}+(A^{*}{)}^{3}+...$$ (4)

which allows us to express the equilibrium abundance of a given species as


$$x_{i}^{*}=1+\sum_{j}A_{ij}^{*}+\sum_{j,k}A_{ik}^{*}A_{kj}^{*}+\sum_{j,k,l}A_{ik}^{*}A_{kl}^{*}A_{lj}^{*}+...$$ (5)

The abundance of a species is affected by direct interactions, but also by the cumulative effects of indirect interactions through all other species [32]. The inverse matrix $(I-A^{*}{)}^{-1}$ therefore contains not only the effects of self-regulation and direct interactions explicitly written in Eqs. (1,2), but also all the indirect effects across increasingly longer chains of species, that together make up the *net* effects between species in a community [32,60,61]. The central question of EC is therefore to understand the relation between A^*^, the direct interactions between species pairs, and $(I-A^{*}{)}^{-1}$, the net effects between species in the context of the full community.

A first fundamental result is that, even in a community where all interactions are competitive ($A_{ij}^{*}<0$ ∀ i≠j), indirect effects involving an even number of species can be positive, so that some competitors positively impact the equilibrium abundance of others. For the communities with EC found in Fig 2A, we find that half of the elements of $(I-A^{*}{)}^{-1}$ are positive, meaning that competitors are similarly exerting positive and negative net effects on others (Fig I in S1 Text). Given this result, the reductionist expectation is that, if we can estimate *A*_*ij*_ experimentally, we can assemble a community by selecting species pairs that can coexist [4], or, at least, by identifying the right “enemy of my enemy” motifs through the powers of A^*^. Yet, to which extent is this possible, and how does the diversity of species and their interactions preclude our capacity to learn multispecies coexistence from *A*?

**Fig 3:**
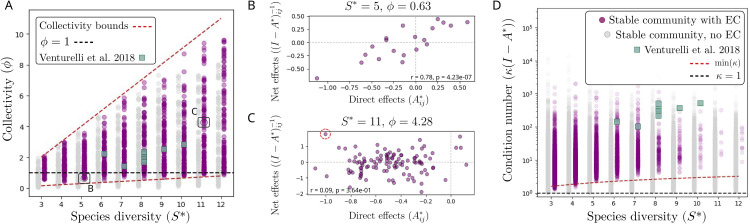
Strong indirect effects decouple pairwise coexistence from community coexistence. Collectivity (ϕ; A) and condition number (κ; D) for stable communities with (purple) and without (gray) EC, sampled within the (μ,σ) range of Fig 2A (see MM). We also plot ϕ,κ values of empirical matrices in [12], the only dataset from Fig 2A for which A^*^ matrices are estimated and ϕ and κ can be measured (green squares). **(A)** Most communities with EC have collectivity (ϕ) close to or larger than 1 (dark dashed line), with analytical estimates predicting $\phi ∼S^{*}|\mu^{*}|$ (red dashed lines, see MM). **(B)** For a community with low ϕ, the direct interactions between species (each off-diagonal element of the matrix of direct interactions $A_{ij}^{*}$, x-axis) and the net effects within the context of the full community (each off-diagonal element of the matrix of net species effects $(I-A^{*}{)}_{ij}^{-1}$, y-axis) are highly correlated (Pearson correlation coefficient *r* and p-value ***p*)**. **(C)** Instead, for a community with high ϕ, direct and net effects become uncorrelated: a species *j* can be a strong competitor when isolated against *i*, but have a positive impact on the abundance of *i* through interactions with the rest of the community (red dashed circle). **(D)** Most simulated and empirical [12] interaction matrices with EC have a condition much larger than 1, implying that small measurement errors of pairwise interactions can potentially induce large errors in the prediction of species coexistence. Condition number κ is plotted in logarithmic scale, and we show the lowest expectation for κ in communities with EC (red dashed line), which increases with diversity as $\kappa ∼S^{*}$ (see MM).

As presented in detail in [32] and [62], the series of Eqs. (4–5), for which we can write the abundance of a given species as a finite sum of chains of indirect effects across all species in the community, does not always converge. If interactions are consistently strong, each longer indirect effect might be more important than shorter ones, to the point where the series of Eq. (5) diverges. We hypothesize here that strong indirect effects provide an explanation to empirical observations of EC. More concretely, the series no longer converges when the *collectivity* metric ϕ, the spectral radius of the interaction matrix A^*^, is larger than 1 ([32,63]). A fundamental consequence of high collectivity is that the direct, pairwise effect of species *j* on species *i* ($A_{ij}^{*}$) becomes uncorrelated from the net effect of species *j* on *i* in the context of the full community ($(I-A^{*}{)}_{ij}^{-1}$). Species *j* can therefore compete strongly with species *i*, but, through complex indirect effects across the community, may exert a positive net effect on it (Figs 3B, 3C and Fig F in S1 Text). The collectivity metric therefore quantifies the extent to which pairwise interactions correlate with (and hence inform about) the coexistence of given species within a species community (see S1 Text II.E and Fig J in S1 Text for a detailed analysis on the correlations between direct and net effects matrices).

Again, we generate initial pools of *S* = 80 species and interaction matrices *A* sampled within the (μ,σ) range of Fig 2A, we sample subcommunities from *A* that fulfill feasibility and stability conditions, and measure ϕ as the modulus of the largest eigenvalue of A^*^ for each stable community (see MM). Communities with no EC (Fig 3A, gray) can have ϕ<1, indicative of weak indirect effects, but also ϕ>1, indicating that indirect effects are strong. For example, many strong but not excluding competitors ($A_{ij}^{*}\approx -0.9$) can have high ϕ and no EC. In comparison, most communities with EC (Fig 3A, purple) have ϕ close to or larger than 1, with ϕ<1 states becoming rarer for larger communities. Predictions from random matrix theory (red dashed lines) indicate that the smallest possible ϕ in these EC communities increases bilinearly with species diversity and interaction strength as $\phi ∼S^{*}|\mu^{*}|$ (see MM). This implies that communities with EC will easily become highly collective and cross the ϕ=1 threshold as diversity increases. Our results indicate that collectivity ϕ, even if ϕ>1 is not a strict condition for EC, provides the simplest metric to quantify the complexity of indirect effects and, therefore, the extent to which pairwise observations provide information about multispecies community composition. As collectivity increases, the likelihood of correctly assembling communities based on pairwise coexistence decreases abruptly see S1 Text II.G and Fig L in S1 Text for simulated tests on assembling communities from pairwise interactions with varying ϕ values).

If species coexistence (Eq. 3) cannot be directly inferred from adding chains of pairwise interactions (Eq. 4–5), an alternative approach could be to infer it by estimating $A_{ij}^{*}$ experimentally and then inverting $(I-A^{*}{)}^{-1}$ numerically. The feasibility of this method can be assessed using the *condition number* $\kappa (I-A^{*})$, which quantifies how small measurement errors in A^*^ are amplified when inverting the matrix, with κ=1 being the best-case scenario where the magnitude of the errors is not amplified [64–66]. We study the same communities for which we previously measured ϕ, and we measure their condition number κ numerically using the singular value decomposition (see MM). Our analysis reveals that κ can remain low for stable communities with weak interactions and no EC, yet it rapidly increases with diversity for communities where strong indirect effects and EC are present (Fig 3D and Fig H in S1 Text). The condition number follows $\kappa (A^{*})=s_{M}(A^{*})/s_{m}(A^{*})$, where *s*_*i*_ are the largest (*M*) and smallest (*m*) singular values of the matrix A^*^, and we use analytical estimates of *s*_*i*_ from random matrix theory to define a lower bound for κ in communities with EC (Fig 3E, red dashed line, see MM). The accuracy of inferring species abundances (and hence species coexistence) by inverting an imprecisely measured A^*^ matrix decays abruptly as κ increases: assuming for example an error of about 10% in measurements of *A*_*ij*_ and a system with a moderate $\kappa (I-A^{*})=5$, only about 50% of the predicted coexisting sets of species would truly coexist, while this precision drops to only 15% of successful predictions for κ=20 (see S1 Text II.F and Fig L in S1 Text for simulated tests on predicting coexistence with measurement errors in *A*). This means that small errors in the measurements of pairwise interactions can lead to substantial errors in the inverse matrix and therefore a decreased predictability of species abundances at equilibrium.

These results align with evidence that reductionist assembly rules work well for small communities of three or four species [4,67], but can break down in more diverse communities where indirect effects can become pervasive [11]. While obtaining exact values of ϕ and κ for a given community would require precise and often inaccessible knowledge of the full interaction matrix A^*^ [43], random matrix theory provides a first approximation to estimate bounds for collectivity metrics only from the statistics of interactions (μ,σ) and not the full matrix (Fig 3, red dashed lines, see [32] for a study of the validity of these estimates when having incomplete knowledge of A^*^). An important open challenge is therefore to quantify how empirical interaction matrices deviate from random approximations, and how these deviations alter indirect effects and collectivity. By doing so, our results suggest that ϕ and κ will offer valuable metrics to infer collectivity and ecological predictability given the statistics of an empirical interaction matrix, as we demonstrate in two proposed experimental case studies in S1 Text II.G.

### Balanced feedback loops maintain stability under strong competition

The results above describe the mechanisms by which strongly competing species can coexist due to indirect effects. Beyond coexistence, experimental communities with EC have also been found to be dynamically stable [4,11], and we study here the mechanisms that guarantee this stability. Mathematically, linear stability can be captured by the eigenvalues of the Jacobian matrix at equilibrium having negative real part [56].

Theoretical results pioneered by May predict that a community will become unstable once diversity, interaction strength and heterogeneity overcome a predictable threshold [33,46,56]. We measure the diversity S^*^ and interaction statistics $\mu^{*},\sigma^{*}$ of stable communities found with the same method of Fig 3A and 3D, and estimate their instability threshold, for which the community would be expected to become unstable if $\mu^{*}<\mu_{c}=\sqrt{S^{*}/2}\sigma^{*}-1$ [33,54]. We find that communities with EC remain stable even if competition is stronger (more negative) than this theoretical limit (Fig M in S1 Text).

This apparent violation is because random matrix predictions only operate for very large communities, as numerically tested in [54], whereas EC experiments and simulations typically involve moderate diversity (Fig 2C). In communities of moderate size, not just the statistical properties of interactions, but their species-level organization influences stability. To understand how realistic communities between from the *S* = 2 and the S→∞ limits can remain stable beyond May’s threshold for competition, we recall the Routh-Hurwitz criteria, necessary conditions for the stability of a Jacobian matrix of any given size [68]. These conditions require that interactions are structured so that negative, stabilizing loops control positive loops that would instead amplify perturbations (see MM, S1 Text II.H and [28,69] for detailed explanations on the Routh-Hurwitz criteria). We find that the stable communities with EC and moderate diversity, even when they violate predictions from random matrix theory, still fulfill the Routh-Hurwitz criteria as expected (Fig M and Fig N in S1 Text). Experimental observations of stable EC under strong competition could therefore be explained by the presence of specific loop architectures in the structure of pairwise interactions [63].

### Emergent coexistence does not require intransitive competition

Coexistence under strong competition has often been linked to intransitivity in theoretical models [1,23,70,71]. Intransitivity can be defined by a lack of hierarchy in species competition, by which no species can become the most dominant of the community. This can be assessed by measuring the abundance of triplets forming a rock-paper-scissors (RPS) motif, or the likelihood of exclusions by species with a lower rank (Low Rank Exclusions, LRE) in the competitive hierarchy, among many possible metrics (see MM and S1 Text II.I for additional intransitivity metrics). Yet, in empirical communities with more than three species, the presence and role of these motifs remain unclear. While some communities appear highly intransitive [72], evidence indicates that intransitivity is in fact rare across species-rich systems [1,25,26]. Experimental communities with EC have been observed with strikingly low intransitivity percentages (0−0.3% of triplets are RPS, 1−3% of exclusions are LRE in [11–13]).

**Fig 4:**
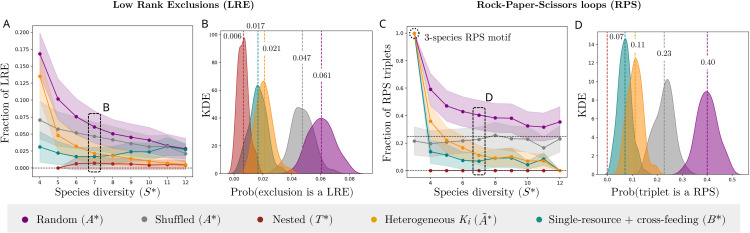
Emergent coexistence does not require intransitive competition. We sample stable communities with EC as done in Fig 3 for the GLV model with different interaction matrices, and measure their intransitivity properties (see MM). We compare intransitivity in communities with EC found under random interactions (A^*^, purple, within the parameter range of Fig 2A), reshuffled A^*^ (gray, reshuffling the off-diagonal elements of each previously found A^*^ matrix), nested interactions (T^*^, red), interactions correlated with carrying capacities (${\tilde{A}}^{*}$, gold) and single-resource competition with cross-feeding ($B^{*}$, green). See MM for a description of how each interaction matrix is built. **(A,B)** Mean (dots) and standard deviation (shaded areas) for the fraction of exclusions where a lower-ranked species excludes a higher-ranked one (LRE) across stable communities with EC, where the rank is defined as the number of wins minus number of losses divided by the number of total interactions (see MM). **(C,D)** Mean (dots) and standard deviation (shaded areas) for the fraction of triplets that are rock-paper-scissors (RPS) across the same stable communities, where a triplet is a trio of species all connected by excluding interactions (see MM). For panels **(B,D)**, Kernel Density Estimates (KDE, see S1 Text **II.**I.4 for the numerical method) allow us to visualize the expected probability distribution of intransitivity metrics in communities with $S^{*}=7$ species.

Here, we measure the fractions of exclusions that are LRE and triplets that are RPS for stable communities with EC sampled again from interaction matrices within the (μ,σ) range of Fig 2A (see MM). We find that intransitivity metrics in communities with EC become lower as species diversity increases (Fig 4 and Fig L and Fig M in S1 Text). In small communities, specific intransitive motifs are common and necessary for coexistence (Fig 2C), consistent with classical RPS observations in small empirical systems [24]. In more complex and collective communities, many interaction chains can rescue species from extinction, and the presence of intransitive motifs tends towards the random expectation as diversity increases (Fig 4). Yet, in some experiments, rock-paper-scissors motifs are almost absent from diverse EC communities assembled in the lab [11,13], which is markedly below the random expectation of ∼25% of triplets being rock-paper-scissors (Fig 4C and 4D, see S1 Text II.I for a detailed analysis of the statistics of RPS triplets in random matrices).

One possible explanation by which EC could emerge with very low intransitivity is that competitive interactions are somehow hierarchical. This could be captured, for example, by a fully nested or triangular interaction matrix *T*, where the upper triangle of the matrix has random interactions as defined above, yet the lower triangle is filled with zeros (see MM). This implies a hierarchy in which species 1 is impacted by all others, species 2 by all except species 1, and so on. However, there is no a priori ordering by which the strengths of these interactions are themselves hierarchical: species 1 is impacted by all others, but the strength of these impacts is still sampled at random.

As recently proposed in [38], another mechanism by which interactions could be hierarchical without the need to impose a triangular structure is related to heterogeneity in carrying capacities. In the main text we have sampled *A*_*ij*_ from a random distribution, yet *A*_*ij*_ are originally defined as $A_{ij}=a_{ij}K_{j}/K_{i}$. If *a* is a random matrix, but *K*_*i*_ are heterogeneous, the resulting *A*_*ij*_ could have row and column correlations related to species carrying capacities (see MM), by which species with highest *K*_*i*_ are the less impacted by others, and the most impactful to others [38,59]. To differentiate from the random uncorrelated matrix *A* we have studied above, we here define $\tilde{A}$ as the matrix with elements $a_{ij}K_{j}/K_{i}$, with *a*_*ij*_ sampled as *A*_*ij*_ above and *K*_*i*_ sampled from a uniform distribution (see MM).

Finally, hierarchical interactions could also emerge from specific properties of the studied microbial communities, in which species compete for a single resource and cooperate via cross-feeding [11,34,55]. Here we define a scenario in which competition is defined by a single trait (the capacity to consume the available resource, $\gamma_{i}$), so that interactions become strictly hierarchical [48]. On top of this, we can incorporate other weak competition mechanisms $\alpha_{ij}$ [73], together with positive cross-feeding *C*_*ij*_ > 0 [34,55]. The new competition matrix *B* that implicitly describes resource competition and cross-feeding writes (see MM)


$$B_{ij}=C_{ij}-\frac{2\gamma_{j}}{\gamma_{i}+\gamma_{j}}-\alpha_{ij}.$$ (6)

When two species compete, the species with the lowest $\gamma_{i}$ perceives stronger competition, resulting in a hierarchical structure where the most efficient consumer dominates [17].

We study the EC and intransitivity properties of communities from the GLV model with a triangular matrix *T*, a row- and column-correlated matrix $\tilde{A}$, and a competition and cross-feeding matrix *B*. We find that the EC regime and fraction of excluding pairs in these structured matrices are qualitatively similar to those of the random GLV model studied above (Fig D and Fig G in S1 Text), yet intransitivity is much lower than the random expectation due to different forms of competitive hierarchies (Fig 4). This result is also consistent with the absence of intransitivity in models with competition-colonization trade-offs recently shown in [21], and confirms that intransitive motifs are not required for EC in models with pairwise interactions. Our results indicate that experimental observations of common and pervasive EC without intransitivity could be related to communities governed by disordered yet hierarchical species interaction matrices with strong indirect effects [11,21].

## Discussion

Species-rich microbial communities assembled in laboratory conditions frequently harbor species that fail to coexist in isolated pairs due to strong competition [4,11,12]. This observation of emergent coexistence (EC) reveals a decoupling between pairwise species interactions and community-level coexistence, challenging the validity of reductionist approaches to community assembly. Mechanisms which have typically been put forward to explain such phenomena are intransitive competition as well as non-pairwise, higher-order interactions [1]. Our research shows that EC is a common outcome in species-rich models with only pairwise interactions, with our analytical and numerical predictions aligning with experimental observations [11] and recent theoretical analyses [21]. Within the useful framework of *structural stability* [74], the explanation for these results is that, across a broad parameter domain, the coexistence volume of many communities (i.e., the space of parameters that leads to species coexistence) differs from the sum of coexistence volumes of their species pairs.

Our work shows that indirect effects can allow the coexistence of a set of strong pairwise competitors, but that these effects become increasingly complex as species diversity increases: Beyond a predictable threshold in interaction strength and species diversity, indirect effects explode in complexity, and outcomes of pairwise experiments alone no longer inform about community composition. This finding provides a mathematical foundation for EC, and imposes an explicit limit to assembling multispecies consortia based on their interactions by pairs. Measuring these pairwise interactions in an empirical community is an extremely complex task [12,42,44,45]. Our findings indicate that, even if one could know the underlying model and measure the species interactions of a community up to a certain precision, predicting collective properties based on pairwise interactions can be limited by strong indirect effects and high collectivity. Taken together, these insights emphasize the need for coarse-grained models that can capture global community properties without relying on precise species-level information [47,75,76].

Our results confirm that EC is intrinsically an emergent phenomenon [6]: in the presence of strong indirect effects, community composition is shaped by pairwise interactions, but cannot be analytically predicted from them alone. This unpredictability arises not from noise or stochasticity, but from the combinatorial explosion of indirect interactions in species-rich systems. While not chaotic in the formal dynamical sense, where small differences in initial conditions lead to diverging temporal trajectories, EC echoes the conceptual lesson of deterministic chaos: that simple, local rules can give rise to globally complex and unpredictable outcomes [77]. In this way, EC exemplifies a broader theme in complex systems science: the fundamental limits to predictability in high-dimensional, nonlinear systems [78]. In ecology, this challenge is often approached through the lens of forecasting temporal responses to perturbations [61,66,79]. Our work extends this perspective by identifying structural limits to reductionist or additive approaches to equilibrium community composition, even in the absence of dynamic fluctuations or stochasticity. These insights have practical implications for the rational design and bottom-up assembly of microbial consortia, with direct applications in biomedicine [80] and environmental restoration [81].

The present study is based on the limit-case assumption that the dynamics of a multispecies community can be effectively described by a GLV-like model of randomly interacting species. Ecological communities are undoubtedly shaped by many intricate processes beyond random pairwise interactions, including consumer-resource dynamics [34], the production of metabolites and toxins [35,82], higher-order multispecies effects [2], demographic stochasticity [83], non-random interactions [20] and complex niche configurations [18]. The coexistence of many species in their natural environments is therefore mediated by a multitude of mechanisms beyond simple indirect effects between competitors. Our work does not claim that indirect effects alone explain the staggering biodiversity observed in nature, nor that the GLV model necessarily provides an accurate coarse-graining of realistic ecological communities. Rather, our goal is to provide a formalism to understand how, even in the simplest possible setting where complex multispecies dynamics could be approximated by a model with random pairwise interactions, the complexity of interaction networks and indirect effects can already generate unexpected patterns of multispecies coexistence. We hypothesize that in more mechanistic models that explicitly account for processes such as resource consumption, metabolite exchange or demographic noise, similar or even stronger irreducible complexity may emerge from the intricate coupling of indirect effects across mechanisms and scales. By bridging empirical observations of EC with minimal models of species coexistence and collectivity, our research provides a step towards understanding the crucial role of indirect effects and ecological complexity in molding emergent community behavior.

## Materials and methods

All mathematical and numerical methods are discussed below and with additional detail in S1 Text. All codes for simulations are found at https://github.com/GuimAguade/EmergentCoexistence.

### Rescaling and parametrizing the GLV model

We study the GLV model of *S* interacting species [9,33,54], with species labeled as *i* = 1,2,...*S* and abundance dynamics following


$$\frac{dN_{i}}{dt}=r_{i}N_{i}\left(1-\frac{N_{i}+\sum_{j\neq i}^{S}a_{ij}N_{j}}{K_{i}}\right).$$ (7)

To focus on the role played by interspecies interactions, we divide species abundances by carrying capacities ($x_{i}\equiv N_{i}/K_{i}$) and rescale interaction strengths relative to self regulation ($A_{ij}=a_{ij}K_{j}/K_{i}$). We refer the reader to, e.g., [38,59] for a discussion on the role of different choices of the GLV model and how different rescalings relate to matrix randomizations. In any case, the normalization allows us to reach equation (2) where *r*_*i*_ and *A*_*ij*_ are the only controlling parameters of the dynamics. To focus on interspecies interactions, in the main text we apply the typical assumption (see, e.g., [32,54]) of homogeneous growth rates *r*_*i*_ = *r* and rescale time as t→rt, so that only interactions encoded in *A*_*ij*_ control the system dynamics. *A*_*ij*_ in the main text are sampled from a Gaussian distribution with μ∈(−2,0.5) and σ∈(0,1) (Fig 2A, 2B and 2C), and we discuss below the impacts of considering heterogeneous carrying capacities. To study the role of network connectivity, we define the probability *p* = *C* at which a given interaction link *A*_*ij*_ is realized, yielding an Erdős-Rényi graph. We present results with *C* = 1 in the main text, and explore the effects of lowering *C* in S1 Text II.A.6. To study the role of growth rate heterogeneity, we sample *r*_*i*_ from a Gaussian distribution $𝒩(1,\sigma_{r})$. We present in S1 Text II.J the results for fixed $\sigma_{r}$ and for the case where $\sigma_{r}=\sigma$, so that growth rates and species interactions have the same standard deviation. We also explore in S1 Text II.A.4 alternative parameterizations for the GLV model with nested, symmetric, antisymmetric, skewed and resource competition and cross-feeding matrices, and in S1 Text II.A.5 the study of EC in the GLV model with migration, the GLV model with saturating interactions, a model with multilayer interactions and a model with sublinear growth dynamics.

### Simulating GLV dynamics

A simulation starts by defining a random matrix of S = 80 interacting species following the parameterizations and models proposed above. For all models, we set random initial conditions $x_{i}(t=0)\in 𝒰[0,1]$, integrate the system for $\Delta t_{1}=3\cdot 10^{3}$ timesteps and check if abundances are the same after $\Delta t_{2}=10^{2}$ additional timesteps to test for stationarity [53,54]. Once a final state is reached, we identify those $S^{*}\leq S$ species that have survived with positive abundance and capture their interactions in A^*^. To draw Fig 2A, for each μ,σ pair we sample a random interaction matrix *A* and random initial conditions, and measure the number of stable states that harbor at least one exclusionary interaction against the number of stable states that harbor at least 3 coexisting species, with or without exclusion interactions. A state has EC if $S^{*}\geq 3$, and there is at least one exclusionary interaction $A_{ij}^{*}<-1$. The fraction of excluding pairs (Fig 2C) is measured as the number of pairs for which either $A_{ij}^{*}$ or $A_{ji}^{*}$ are smaller than -1, divided by the total number of pairs of surviving species, $S^{*}(S^{*}-1)/2$. The GLV equations are integrated numerically using scipy.integrate.solve_ivp with an adaptive Runge-Kutta (RK45) scheme; species whose abundance falls below a fixed extinction threshold are removed by setting their abundance and growth rate to zero during integration.

### Sampling stable subsets of A^*^

To find stable states from Eq. (1) without the need to run the complete dynamics, we use the following complementary technique from [54] that allows to efficiently generate Figs 3 and 4. Given a matrix of interactions *A* defined as above, we select a random subset of species $S^{*}\leq S$ and their interactions A^*^. We first ask if this set of species is feasible by solving Eq. (3) and studying if all final abundances are positive (all species in the subset survive). We then obtain the Jacobian matrix for this species subset from $J_{ij}^{*}=-r_{i}x_{i}^{*}A_{ij}^{*}$, and check if all eigenvalues of the Jacobian are negative for linear stability. The subsets of A^*^ that fulfill these two conditions are stable and feasible solutions of equation (2) in the absence of migration [54]. We then use the same methods as above to evaluate if these subsets harbor EC. Previous work [54] analyzed the differences between stable states found by simulating the complete dynamics and those found by sampling subsets and evaluating their stability and feasibility. The latter method sometimes finds smaller and slightly more competitive communities, that could be invaded by weaker competitors from the pool when simulating the complete dynamics with all initial conditions positive. However, the two methods generate EC communities that fulfill feasibility and stability constraints and hence are valid solutions for Eq. (2) given certain initial conditions. To ensure that enough stable communities are found with different degrees of EC (Fig 3) and intransitivity (Fig 4) to observe accurate trends, we sample 10^7^ species subsets for each diversity $S^{*}\in [3,12]$ and different (μ,σ) values, and test them for feasibility, stability and EC.

### The boundaries of the EC regime

We use simple statistical arguments to find a first estimate for the boundaries of the EC regime in the (μ,σ) parameter space (Fig 2A and 2B, red lines). We propose two *necessary*, albeit not *sufficient* conditions, that provide good estimates for the EC regime in the case of large *S* and random and uncorrelated interactions presented in the main text. These conditions are based on the following heuristic argument: for EC to be present in the final surviving community ($A_{ij}^{*}<-1$), it is necessary that at least one strong competition term was already present in the initial species pool ($A_{ij}<-1$), and that at least two species coexist in a pair so that they can sustain the excluded one (*A*_*kl*_,$A_{lk}>-1$, see S1 Text II.A for a detailed analysis). We therefore search for the minimal (μ,σ) combination for which at least one element within S(S−1) elements of *A* will be exclusionary ($A_{ij}<-1$, S1 Text II.A). The probability of $A_{ij}<-1$ is $P=1-{\left(1-\Phi \left(-(1+\mu )/\sigma \right)\right)}^{S(S-1)}$, where Φ(z) is the cumulative distribution function (CDF) of the standard normal distribution. If *S* is large, this function predicts a sharp transition from *P* = 0 to *P* = 1 for given (μ,σ) values ($\sigma_{1}^{c}$ in Fig 2). Using a similar argument for the case where most competitive terms are exclusionary and dynamics fall within the competitive exclusion regime, we assume that to find at least two species that coexist and can sustain a third one in the surviving interaction matrix A^*^, we need at least a pair of coexisting species in the original pool (*A*_*kl*_,$A_{lk}>-1$). Using similar probabilistic methods, we therefore search for the maximal (μ,σ) combination for which at least one species pair is not exclusionary (*A*_*kl*_,$A_{lk}>-1$) and can rescue a third excluded species. Note that these boundaries define necessary, but not sufficient conditions: for example, for the case of purely symmetric interactions, one finds that even if the initial pool can contain exclusionary elements ($A_{ij}<-1$, $\sigma >\sigma_{1}^{c}$), this will not ensure that EC is present in the final community, as symmetric *A*_*ij*_ elements cannot yield EC (see S1 Text II.A for a mathematical proof of the absence of EC in symmetric interaction matrices).

### The maximum fraction of excluding pairs

Given a stable community that harbors EC (at least one exclusionary element, $A_{ij}^{*}<-1$), we measure the fraction of excluding pairs as the fraction of pairs of species (*i*,*j*) in that community for which at least one of the two interactions $A_{ij}^{*}$ or $A_{ji}^{*}$ is exclusionary. In Fig 2C, we plot in purple these values for all communities harboring EC found in the simulations of Fig 2A. As described in full detail in S1 Text II.B, to obtain a first analytical estimate for the maximum fraction of pairs that a community of a given size can contain, we merge two results: given a (μ,σ) pair of values that generate a matrix *A*, we want to know (i) the maximum possible species richness S^*^, and (ii) the maximum fraction of $A_{ij}^{*}<-1$ elements of a stable community found for that interaction matrix. (i) Previous work on the GLV model provided an analytical estimate for the maximum species richness S^*^ that is possible to observe given (μ,σ) inside the multistability domain [54]. This method is a few-species approximation of classical results in community ecology, for which a community becomes unstable beyond a given size S^*^ and interaction strength and heterogeneity (μ,σ) [33,46]. Using this method, we provide a first estimate for the largest possible community S^*^ that can be stable given an original pool of interactions *A* sampled from a (μ,σ) pair. (ii) We use probabilistic arguments to estimate the maximum fraction of $A_{ij}^{*}<-1$ elements given (μ,σ). Following the observation that most stable states in the multistability domain of the GLV model tend to harbor similar or lower competition than the original pool [54], we assume that the maximum fraction of $A_{ij}^{*}<-1$ elements is, at most, the fraction of $A_{ij}<-1$ elements in the pool, so that stable states that contain more competitive interactions than the original pool are assumed to be rare. The problem then is simplified to estimating the fraction of $A_{ij}<-1$ elements in a Gaussian sample 𝒩(μ,σ) of size S(S−1). Taken together, for the whole (μ,σ) range of Fig 2A, we provide a statistical bound for both the largest possible community (S^*^, x-axis) and the maximum fraction of excluding pairs it can contain (y-axis) that together make the red dashed line of Fig 2C (see S1 Text II.B for a step-by-step development). Finally, the minimum fraction of excluding pairs in a community of S^*^ species is simply 1 over the number of pairs $S^{*}(S^{*}-1)/2$, because a community with zero excluding pairs will not harbor EC, as we show with a dark dashed line in Fig 2C.

### Analytical bounds on collectivity ϕ

In Fig 3A we sample communities with and without EC from the GLV model within the parameter range (μ,σ) of Fig 2A, and measure their diversity S^*^ and the spectral radius ϕ, the modulus of the largest eigenvalue of A^*^ obtained with numpy.linalg.eigvals. To obtain an estimate for the analytical minimum and maximum collectivity ϕ for each value of S^*^, we use an estimate for the spectral radius of a random matrix, which for uncorrelated and fully connected matrices simplifies to $\phi =\text{max}\{(S^{*}-1)|\mu^{*}|,\sqrt{\left(S-1\right)}\sigma^{*}\}$ [32,56]. This simplifies finding min$\phi (S^{*})$ and max$\phi (S^{*})$ (Fig 3A) to finding min and max of $|\mu^{*}|,\sigma^{*}$ for each S^*^, given a (μ,σ) range as explained above and in S1 Text II.E and [54] in more detail.

### Analytical bounds on condition number κ

In Fig 3D we follow the same sampling method and parameter range as for ϕ in Fig 3A described above, and we measure the diversity S^*^ and the condition number κ of the interaction matrix A^*^ of stable communities with (purple) and without (gray) EC. The condition number is estimated numerically in python using the numpy.linalg.cond method from numpy. To provide an analytical estimate for the smallest condition number of a given matrix A^*^, we recall that $\kappa (A^{*})=s_{M}(A^{*})/s_{m}(A^{*})$, where *s*_*i*_ are the largest (*M*) and smallest (*m*) singular values of the matrix A^*^. The smallest condition number is found from min $\kappa =\text{min}(s_{M}(A^{*}))/\text{max}(s_{m}(A^{*}))$, and the largest and smallest singular values have an analytical estimate for random matrices given $S^{*}$ and $\mu^{*},\sigma^{*}$, as we describe in detail in S1 Text II.F and in [64].

### Stability and feedback loops

An equilibrium community, defined by Eq. (3), is linearly stable if it recovers from infinitesimal perturbations, which requires that all eigenvalues of the Jacobian matrix evaluated at that equilibrium point are negative [56]. A classical result for large random matrices finds that a Jacobian with random interactions becomes unstable once *S*, μ or σ overcome a predictable threshold [33,46], yet we find that this threshold does not apply for communities with a moderate number of species and strong interactions. To understand what conditions ensure stability in these communities given that the classical May threshold is not fulfilled, we describe in detail in S1 Text II.H.3 the Routh-Hurwitz criteria, which provide strict necessary conditions for the stability of a Jacobian independently of its size, and allow for interesting ecological interpretations as better discussed in [28,69,84]. The Routh-Hurwitz criteria link linear stability to conditions on the coefficients *C*_*i*_ of the characteristic polynomial of the Jacobian matrix, found by solving det(J−λI)=0 with $J_{ij}=r_{i}x_{i}^{*}A_{ij}^{*}$ (see S1 Text I.K for the step-by-step proof, [28,69]). For each community sampled from the GLV model in Fig 3A, we observe that it fulfills the Routh-Hurwitz criteria for stability by finding *C*_*i*_ numerically with numpy.poly and measuring the second Routh-Hurwitz stability condition, which requires $\Lambda_{2}=C_{1}C_{2}-C_{3}>0$ as previously done in [84] (Fig M and Fig N in S1 Text).

### Intransitive competition motifs

We measure intransitivity by the fraction of LRE and RPS in comparison with null expectations, and discuss other intransitivity metrics yielding similar results in S1 Text II.I.3. Following, e.g., [11,13], we define the rank of each species in a community as the number of species it excludes, minus the number of species it is excluded by. A low rank exclusion happens when a species with lower rank excludes a species with higher rank, which requires different motifs with at least 4 exclusions. We correct the LRE metric in Fig 4A for communities with at least four exclusionary interactions, to avoid underestimating LRE by studying many communities that have less than 4 excluding interactions (in Fig F in S1 Text we plot the uncorrected metric which yields very low LRE fractions for all values of S^*^). We define a triplet as a set of three species connected by competitive exclusion ($A_{ij}^{*}<-1$). A rock-paper-scissors triplet has a non-dominant architecture (A excludes B, B excludes C, C excludes A), and given a random triplet, 2 out of 8 configurations are RPS, making the null expectation for the fraction of triplets in a community *RPS* = 0.25. To visualize the probability distribution of rock-paper-scissors and low rank exclusion fractions (Fig 4), we sample stable communities with EC as described for Fig 3, measure the mean and standard deviation of observed intransitivity metrics across them and plot kernel density estimates (KDE) for these distributions as detailed in S1 Text II.I.4. We show similar results with other intransitivity metrics in S1 Text II.I.7 and Fig Q in S1 Text, by which intransitivity becomes rare in large communities with EC.

### Hierarchy emerging from heterogeneous carrying capacities

In the main text, we study a model in which interactions *A*_*ij*_ are sampled following different randomization schemes. As recently proposed in [38], one possible mechanism by which interaction hierarchies can emerge is that carrying capacities are heterogeneous, and that this in turn impacts the structure of *A*. For example, one could assume that the original interactions *a*_*ij*_ are random, implying that the normalization $A_{ij}=a_{ij}K_{j}/K_{i}$ (Eq. 3) is no longer random, but carries some row and column structure induced by the carrying capacities *K*_*i*_. Following [38], we generate *K*_*i*_ uniformly from 𝒰[0.5,1.5], *a*_*ij*_ from 𝒩(μ,σ) as typically done for *A*, and define a new correlated interaction matrix $\tilde{A}$ (to differentiate from *A* in the main text) as ${\tilde{A}}_{ij}=a_{ij}K_{j}/K_{i}$. If *a*_*ij*_ are random interactions, ${\tilde{A}}_{ij}$ carry row and column correlations, by which the species with largest *K*_*i*_ is the most impactful to others and the less impacted by others, and so on. We refer the reader to [59] for an insightful discussion on whether *a* (the matrix for the interactions between abundances *N*_*i*_) or *A* (the matrix for the interactions between relative yields *x*_*i*_) are the matrices that one should consider as random within the perspective of *disordered* interactions in community ecology.

### Nested and cross-feeding hierarchical matrices

To study if EC is still possible in other highly hierarchical (and hence, transitive) architectures, we build an idealized triangular matrix and a more realistic resource competition and cross-feeding matrix. The nested or triangular matrix *T* is generated from *A* within the same parameter range of the whole main text, but imposing that all elements in the lower triangle of *A* are zero ($T_{ij}=A_{ij}$ if *i* < *j*, *T*_*ij*_ = 0 if *i* > *j*). This generates a hierarchy in the adjacency matrix (who impacts who), but not necessary in the strength of interactions, meaning that LRE could still be possible a priori. For *B*, positive γ values are sampled from 𝒰[0.3,0.7] and sorted by decreasing order to build a hierarchical matrix; $\alpha_{ij}$ are sampled from 𝒩(0.1,0.01); *C*_*ij*_ are sampled from 𝒰[0.0,1.0] (see S1 Text I.D for a detailed explanation). We do not have empirical support for these parameters and use them only as a proof of concept to explain how hierarchical competition and random cross-feeding can lead to transitive communities with EC. If the strength or heterogeneity of $\alpha_{ij}$ increases, the model becomes similar to the random interactions model. If the strength or heterogeneity of *C*_*ij*_ increases, cooperative interactions dominate and the model transitions towards the outgrowth regime of Fig 2A. Once these matrices are defined, we sample stable communities from the GLV model with different iterations of these matrices and *S*=80 as explained above.

## Supporting information

S1 Text The Supplementary Material is found in the S1 Text, which contains information on the methods, simulations and models, the implementation and results of the project, as well as supplementary figures A-S. (PDF)

## References

[1] Levine JM, Bascompte J, Adler PB, Allesina S. Beyond pairwise mechanisms of species coexistence in complex communities. Nature. 2017;546(7656):56–64. doi: 10.1038/nature22898 28569813

[2] Billick I, Case TJ. Higher Order Interactions in Ecological Communities: What Are They and How Can They be Detected?. Ecology. 1994;75(6):1529–43. doi: 10.2307/1939614

[3] Widder S, Allen RJ, Pfeiffer T, Curtis TP, Wiuf C, Sloan WT, et al. Challenges in microbial ecology: building predictive understanding of community function and dynamics. ISME J. 2016;10(11):2557–68. doi: 10.1038/ismej.2016.45 27022995 PMC5113837

[4] Friedman J, Higgins LM, Gore J. Community structure follows simple assembly rules in microbial microcosms. Nat Ecol Evol. 2017;1(5):109. doi: 10.1038/s41559-017-0109 28812687

[5] Anderson PW. More is different: broken symmetry and the nature of the hierarchical structure of science. Science. 1972;177(4047):393–6. 17796623 10.1126/science.177.4047.393

[6] Artime O, De Domenico M. From the origin of life to pandemics: emergent phenomena in complex systems. Philos Trans A Math Phys Eng Sci. 2022;380(2227):20200410. doi: 10.1098/rsta.2020.0410 35599559 PMC9125231

[7] Coyte KZ, Rao C, Rakoff-Nahoum S, Foster KR. Ecological rules for the assembly of microbiome communities. PLoS Biol. 2021;19(2):e3001116. doi: 10.1371/journal.pbio.3001116 33606675 PMC7946185

[8] Arya S, George AB, O’Dwyer J. The architecture of theory and data in microbiome design: towards an S-matrix for microbiomes. Curr Opin Microbiol. 2025;83:102580. doi: 10.1016/j.mib.2025.102580 39848217

[9] van den Berg NI, Machado D, Santos S, Rocha I, Chacón J, Harcombe W, et al. Ecological modelling approaches for predicting emergent properties in microbial communities. Nat Ecol Evol. 2022;6(7):855–65. doi: 10.1038/s41559-022-01746-7 35577982 PMC7613029

[10] Hu J, Amor DR, Barbier M, Bunin G, Gore J. Emergent phases of ecological diversity and dynamics mapped in microcosms. Science. 2022;378(6615):85–9. doi: 10.1126/science.abm7841 36201585

[11] Chang C-Y, Bajić D, Vila JCC, Estrela S, Sanchez A. Emergent coexistence in multispecies microbial communities. Science. 2023;381(6655):343–8. doi: 10.1126/science.adg0727 37471535

[12] Venturelli OS, Carr AC, Fisher G, Hsu RH, Lau R, Bowen BP, et al. Deciphering microbial interactions in synthetic human gut microbiome communities. Mol Syst Biol. 2018;14(6):e8157. doi: 10.15252/msb.20178157 29930200 PMC6011841

[13] Higgins LM, Friedman J, Shen H, Gore J. Co-occurring soil bacteria exhibit a robust competitive hierarchy and lack of non-transitive interactions. BioRxiv. 2017;:175737.

[14] Kehe J, Ortiz A, Kulesa A, Gore J, Blainey PC, Friedman J. Positive interactions are common among culturable bacteria. Sci Adv. 2021;7(45):eabi7159. doi: 10.1126/sciadv.abi7159 34739314 PMC8570599

[15] Kraft NJB, Godoy O, Levine JM. Plant functional traits and the multidimensional nature of species coexistence. Proc Natl Acad Sci U S A. 2015;112(3):797–802. doi: 10.1073/pnas.1413650112 25561561 PMC4311865

[16] Buche L, Spaak JW, Jarillo J, De Laender F. Niche differences, not fitness differences, explain predicted coexistence across ecological groups. Journal of Ecology. 2022;110(11):2785–96. doi: 10.1111/1365-2745.13992

[17] Tilman D. Resource competition and community structure. Princeton University Press. 1982. 7162524

[18] Chesson P. Mechanisms of Maintenance of Species Diversity. Annu Rev Ecol Syst. 2000;31(1):343–66. doi: 10.1146/annurev.ecolsys.31.1.343

[19] Hart SP. How does facilitation influence the outcome of species interactions?. Journal of Ecology. 2023;111(10):2094–104. doi: 10.1111/1365-2745.14189

[20] de Ruiter PC, Neutel AM, Moore JC. Energetics, patterns of interaction strengths, and stability in real ecosystems. Science. 1995;269(5228):1257–60. doi: 10.1126/science.269.5228.1257 17732112

[21] Miller ZR, Max D. Multispecies Coexistence Emerges From Pairwise Exclusions in Communities With Competitive Hierarchy. Ecol Lett. 2025;28(9):e70206. doi: 10.1111/ele.70206 40961254 PMC12443417

[22] Levine JM. Indirect facilitation: evidence and predictions from a riparian community. Ecology. 1999;80(5):1762–9. doi: 10.1890/0012-9658(1999)080[1762:ifeapf]2.0.co;2

[23] Gallien L, Zimmermann NE, Levine JM, Adler PB. The effects of intransitive competition on coexistence. Ecol Lett. 2017;20(7):791–800. doi: 10.1111/ele.12775 28547799

[24] Kerr B, Riley MA, Feldman MW, Bohannan BJM. Local dispersal promotes biodiversity in a real-life game of rock-paper-scissors. Nature. 2002;418(6894):171–4. doi: 10.1038/nature00823 12110887

[25] Godoy O, Stouffer DB, Kraft NJ, Levine JM. Intransitivity is infrequent and fails to promote annual plant coexistence without pairwise niche differences. 2017. 10.1002/ecy.1782 28241383

[26] Koch F, Neutel A-M, Barnes DKA, Tielbӧrger K, Zarfl C, Allhoff KT. Competitive hierarchies in bryozoan assemblages mitigate network instability by keeping short and long feedback loops weak. Commun Biol. 2023;6(1):690. doi: 10.1038/s42003-023-05060-1 37402788 PMC10319822

[27] Wootton JT. The nature and consequences of indirect effects in ecological communities. Annu Rev Ecol Syst. 1994;25(1):443–66. doi: 10.1146/annurev.es.25.110194.002303

[28] Levins R. Discussion paper: the qualitative analysis of partially specified systems. Ann N Y Acad Sci. 1974;231(1):123–38. doi: 10.1111/j.1749-6632.1974.tb20562.x 4522890

[29] Levine SH. Competitive Interactions in Ecosystems. The American Naturalist. 1976;110(976):903–10. doi: 10.1086/283116

[30] Dambacher JM, Li HW, Rossignol PA. Relevance of community structure in assessing indeterminacy of ecological predictions. Ecology. 2002;83(5):1372–85. doi: 10.1890/0012-9658(2002)083[1372:rocsia]2.0.co;2

[31] Montoya JM, Woodward G, Emmerson MC, Solé RV. Press perturbations and indirect effects in real food webs. Ecology. 2009;90(9):2426–33. doi: 10.1890/08-0657.1 19769121

[32] Zelnik YR, Galiana N, Barbier M, Loreau M, Galbraith E, Arnoldi JF. How collectively integrated are ecological communities? Ecology Letters. 2024;27(1):e14358. 10.1111/ele.14358 38288867

[33] Bunin G. Ecological communities with Lotka-Volterra dynamics. Phys Rev E. 2017;95(4–1):042414. doi: 10.1103/PhysRevE.95.042414 28505745

[34] Estrela S, Vila JCC, Lu N, Bajić D, Rebolleda-Gómez M, Chang C-Y, et al. Functional attractors in microbial community assembly. Cell Syst. 2022;13(1):29-42.e7. doi: 10.1016/j.cels.2021.09.011 34653368 PMC8800145

[35] Pfeiffer T, Bonhoeffer S. Evolution of cross-feeding in microbial populations. Am Nat. 2004;163(6):E126-35. doi: 10.1086/383593 15266392

[36] Lajaaiti I, Kéfi S, Arnoldi J-F. How biotic interactions structure species’ responses to perturbations. Proc Biol Sci. 2024;291(2032):20240930. doi: 10.1098/rspb.2024.0930 39378997 PMC11461057

[37] Lajaaiti I, Kéfi S, Loreau M, Ardichvili A, Arnoldi JF. Revealing the organization of species stability in ecological communities. bioRxiv. 2025. 2025–03.

[38] Mizrahi SP, Lee H, Goyal A, Owen E, Gore J. Structured interactions explain the absence of keystone species in synthetic microcosms. bioRxiv. 2025. 2025–03. 10.1093/ismejo/wraf211 PMC12510465 40981661

[39] Mallmin E, Traulsen A, De Monte S. Chaotic turnover of rare and abundant species in a strongly interacting model community. Proc Natl Acad Sci U S A. 2024;121(11):e2312822121. doi: 10.1073/pnas.2312822121 38437535 PMC10945849

[40] Rosenbaum B, Fronhofer EA. Confronting population models with experimental microcosm data: from trajectory matching to state‐space models. Ecosphere. 2023;14(4). doi: 10.1002/ecs2.4503

[41] Arya S, George AB, O’Dwyer JP. Sparsity of higher-order landscape interactions enables learning and prediction for microbiomes. Proc Natl Acad Sci U S A. 2023;120(48):e2307313120. doi: 10.1073/pnas.2307313120 37991947 PMC10691334

[42] Momeni B, Xie L, Shou W. Lotka-Volterra pairwise modeling fails to capture diverse pairwise microbial interactions. Elife. 2017;6:e25051. doi: 10.7554/eLife.25051 28350295 PMC5469619

[43] Remien CH, Eckwright MJ, Ridenhour BJ. Structural identifiability of the generalized Lotka-Volterra model for microbiome studies. R Soc Open Sci. 2021;8(7):201378. doi: 10.1098/rsos.201378 34295510 PMC8292772

[44] Ram Y, Dellus-Gur E, Bibi M, Karkare K, Obolski U, Feldman MW, et al. Predicting microbial growth in a mixed culture from growth curve data. Proc Natl Acad Sci U S A. 2019;116(29):14698–707. doi: 10.1073/pnas.1902217116 31253703 PMC6642348

[45] Lubiana Botelho L, Jeynes-Smith C, Vollert SA, Bode M. Calibrated Ecosystem Models Cannot Predict the Consequences of Conservation Management Decisions. Ecol Lett. 2025;28(1):e70034. doi: 10.1111/ele.70034 39737694

[46] May RM. Will a large complex system be stable?. Nature. 1972;238(5364):413–4. doi: 10.1038/238413a0 4559589

[47] Barbier M, Arnoldi J-F, Bunin G, Loreau M. Generic assembly patterns in complex ecological communities. Proc Natl Acad Sci U S A. 2018;115(9):2156–61. doi: 10.1073/pnas.1710352115 29440487 PMC5834670

[48] Barbier M, de Mazancourt C, Loreau M, Bunin G. Fingerprints of High-Dimensional Coexistence in Complex Ecosystems. Phys Rev X. 2021;11(1). doi: 10.1103/physrevx.11.011009

[49] Camacho-Mateu J, Lampo A, Sireci M, Muñoz MA, Cuesta JA. Sparse species interactions reproduce abundance correlation patterns in microbial communities. Proc Natl Acad Sci U S A. 2024;121(5):e2309575121. doi: 10.1073/pnas.2309575121 38266051 PMC10853627

[50] Pasqualini J, Maritan A, Rinaldo A, Facchin S, Savarino E, Altieri A. Microbiomes Through The Looking Glass. In: 2024. https://doi.org/arXiv:240607465

[51] Stein RR, Bucci V, Toussaint NC, Buffie CG, Rätsch G, Pamer EG, et al. Ecological modeling from time-series inference: insight into dynamics and stability of intestinal microbiota. PLoS Comput Biol. 2013;9(12):e1003388. doi: 10.1371/journal.pcbi.1003388 24348232 PMC3861043

[52] Dal Bello M, Lee H, Goyal A, Gore J. Resource-diversity relationships in bacterial communities reflect the network structure of microbial metabolism. Nat Ecol Evol. 2021;5(10):1424–34. doi: 10.1038/s41559-021-01535-8 34413507

[53] Aguadé-Gorgorió G, Arnoldi J-F, Barbier M, Kéfi S. A taxonomy of multiple stable states in complex ecological communities. Ecol Lett. 2024;27(4):e14413. doi: 10.1111/ele.14413 38584579

[54] Aguadé-Gorgorió G, Kéfi S. Alternative cliques of coexisting species in complex ecosystems. J Phys Complex. 2024;5(2):025022. doi: 10.1088/2632-072x/ad506a

[55] Goldford JE, Lu N, Bajić D, Estrela S, Tikhonov M, Sanchez-Gorostiaga A, et al. Emergent simplicity in microbial community assembly. Science. 2018;361(6401):469–74. doi: 10.1126/science.aat1168 30072533 PMC6405290

[56] Allesina S, Tang S. Stability criteria for complex ecosystems. Nature. 2012;483(7388):205–8. doi: 10.1038/nature10832 22343894

[57] Koch F, Neutel AM, Barnes DK, Allhoff KT. Skewness enables stabilising effect of hierarchy in complex competition networks. bioRxiv. 2024. 2024–01.

[58] Aguadé‐Gorgorió G, Lajaaiti I, Arnoldi J, Kéfi S. Unpacking sublinear growth: diversity, stability and coexistence. Oikos. 2024;2025(1). doi: 10.1111/oik.10980

[59] Barbier M. Disordered systems in community ecology: a tutorial. 2025.

[60] Yodzis P. The Indeterminacy of Ecological Interactions as Perceived Through Perturbation Experiments. Ecology. 1988;69(2):508–15. doi: 10.2307/1940449

[61] Bender EA, Case TJ, Gilpin ME. Perturbation Experiments in Community Ecology: Theory and Practice. Ecology. 1984;65(1):1–13. doi: 10.2307/1939452

[62] Gómez-Ambrosi C, Calleja-Solanas V. Measuring net effects in signed ecological and social network. In: 2025. https://arxiv.org/abs/250109190 10.1038/s41598-026-62819-2 42481621

[63] Zelnik. The collectivity limit is not equivalent to May’s classical limit on stability (May, 1972). A community can be stable within May’s bounds, but have strong indirect effects lea ding to EC. 2024.

[64] Edelman A. Eigenvalues and Condition Numbers of Random Matrices. SIAM J Matrix Anal & Appl. 1988;9(4):543–60. doi: 10.1137/0609045

[65] Trefethen LN, Bau D. Numerical linear algebra. SIAM. 2022.

[66] Gilpin W. Optimization hardness constrains ecological transients. PLoS Comput Biol. 2025;21(5):e1013051. doi: 10.1371/journal.pcbi.1013051 40324147 PMC12074658

[67] Lee H, Bloxham B, Gore J. Resource competition can explain simplicity in microbial community assembly. Proc Natl Acad Sci U S A. 2023;120(35):e2212113120. doi: 10.1073/pnas.2212113120 37603734 PMC10469513

[68] Bodson M. Explaining the Routh&ndash;Hurwitz Criterion: A Tutorial Presentation [Focus on Education]. IEEE Control Syst. 2020;40(1):45–51. doi: 10.1109/mcs.2019.2949974

[69] Dambacher JM, Luh H-K, Li HW, Rossignol PA. Qualitative stability and ambiguity in model ecosystems. Am Nat. 2003;161(6):876–88. doi: 10.1086/367590 12858273

[70] Allesina S, Levine JM. A competitive network theory of species diversity. Proc Natl Acad Sci U S A. 2011;108(14):5638–42. doi: 10.1073/pnas.1014428108 21415368 PMC3078357

[71] Laird RA, Schamp BS. Competitive intransitivity promotes species coexistence. Am Nat. 2006;168(2):182–93. doi: 10.1086/506259 16874628

[72] Soliveres S, Maestre FT, Ulrich W, Manning P, Boch S, Bowker MA, et al. Intransitive competition is widespread in plant communities and maintains their species richness. Ecol Lett. 2015;18(8):790–8. doi: 10.1111/ele.12456 26032242 PMC5321564

[73] Ghoul M, Mitri S. The Ecology and Evolution of Microbial Competition. Trends Microbiol. 2016;24(10):833–45. doi: 10.1016/j.tim.2016.06.011 27546832

[74] Saavedra S, Rohr RP, Bascompte J, Godoy O, Kraft NJB, Levine JM. A structural approach for understanding multispecies coexistence. Ecological Monographs. 2017;87(3):470–86. doi: 10.1002/ecm.1263

[75] Moran J, Tikhonov M. Defining Coarse-Grainability in a Model of Structured Microbial Ecosystems. Phys Rev X. 2022;12(2). doi: 10.1103/physrevx.12.021038

[76] Marcus S, Bunin G. When can few-species models describe dynamics within a complex community?. Ecological Modelling. 2025;506:111137.

[77] May RM. Simple mathematical models with very complicated dynamics. Nature. 1976;261(5560):459–67. doi: 10.1038/261459a0 934280

[78] Boffetta G. Predictability: a way to characterize complexity. Physics Reports. 2002;356(6):367–474. doi: 10.1016/s0370-1573(01)00025-4

[79] Kawatsu K. Unraveling emergent network indeterminacy in complex ecosystems: A random matrix approach. Proc Natl Acad Sci U S A. 2024;121(27):e2322939121. doi: 10.1073/pnas.2322939121 38935564 PMC11228516

[80] Eng A, Borenstein E. Microbial community design: methods, applications, and opportunities. Curr Opin Biotechnol. 2019;58:117–28. doi: 10.1016/j.copbio.2019.03.002 30952088 PMC6710113

[81] Solé R, Maull V, Amor DR, Mauri JP, Núria C-P. Synthetic Ecosystems: From the Test Tube to the Biosphere. ACS Synth Biol. 2024;13(12):3812–26. doi: 10.1021/acssynbio.4c00384 39570594 PMC11669164

[82] Granato ET, Meiller-Legrand TA, Foster KR. The Evolution and Ecology of Bacterial Warfare. Curr Biol. 2019;29(11):R521–37. doi: 10.1016/j.cub.2019.04.024 31163166

[83] Zhou J, Ning D. Stochastic community assembly: does it matter in microbial ecology?. Microbiology and Molecular Biology Reviews. 2017;81(4):10–1128. 10.1128/MMBR.00002-17 PMC5706748 29021219

[84] Neutel A-M, Thorne MAS. Interaction strengths in balanced carbon cycles and the absence of a relation between ecosystem complexity and stability. Ecol Lett. 2014;17(6):651–61. doi: 10.1111/ele.12266 24636521 PMC4285907

