# Supplementary material for: Emergent coexistence and the limits of reductionism in ecological communities
Source: PLoS Comput Biol. 2026 Mar 27;22(3):e1014116. doi: 10.1371/journal.pcbi.1014116 (PMC13048502; doi:10.1371/journal.pcbi.1014116)
Supplement: S1 Text — (PDF) [file pcbi.1014116.s001.pdf]

Supplementary Material to: Emergent coexistence and the limits of reductionism in ecological communities

Guim Aguadé-Gorgorió[1,*] and Sonia Kéfi[1,2]

[1] *ISEM, University of Montpellier, CNRS, IRD, EPHE, Montpellier, France*

[2] *Santa Fe Institute, 1399 Hyde Park Road, Santa Fe, NM 87501, USA*

(Dated: March 17, 2026)

The structure of the following Supplementary Material is as follows: First, we present and define the fundamental problem under study and describe the numerical simulations and mathematical methods to explore it. Second, we present a detailed analysis of the methods and results following the structure of figures and results of the main text, together with additional mathematical developments, figures and considerations.

All codes discussed in the present Supplementary Material are available at:

https://github.com/GuimAguade/EmergentCoexistence.

CONTENTS

I. METHODS, SIMULATIONS AND MODELS 4

A. Description of the problem and the GLV model 4

B. Pairwise exclusions, equilibria, net interactions and indirect effects 5

1. Pairwise exclusions 5

2. Indirect effects in three-species toy models 6

3. Equilibria, net interactions and the Jacobian matrix 7

C. Finding stable communities 8

1. Simulating ecological dynamics 9

2. Sampling and testing random species subsets 9

D. Species interactions from random distributions and disordered models in ecology 10

E. Network structure and connectivity in microbial communities 11

* Corresponding author:guimaguade@gmail.com

F. The GLV model with other interaction matrices 11

G. Other dynamical models with pairwise interactions 13

1. Saturating interactions with a Holling response 13

2. Multilayer interactions with Allee Effects 14

3. Sublinear growth dynamics coupled to linear interactions 14

II. IMPLEMENTATION AND RESULTS 15

A. Emergent coexistence across the parameter space (Figure 2A) 15

1. The minimal competition necessary for emergent coexistence 15

2. The EC boundary and multistability 17

3. Maximum competition allowed for emergent coexistence 18

4. Emergent coexistence under alternative interaction structures 19

5. Emergent coexistence in other dynamical models with pairwise interactions 20

6. Connectivity and its effects on coexistence and phase space structure 21

B. The fraction of excluding pairs (Figure 2C) 22

1. Gathering data on the fraction of excluding pairs 23

2. Maximum fraction of excluding pairs a community can sustain 25

3. Fraction of pairs in sparse and hierarchical matrices 26

C. Interaction patterns in A^* and coexistence 26

D. All-to-all competition but positive net effects 28

E. Collectivity and net effects (Figure 3A-C) 28

1. Computing the Spectral Radius ϕ 29

2. Data plotted in Figures 3A,D 29

3. Estimates for minimal and maximal collectivity 29

4. Collectivity and irreducible interaction chains 31

F. Condition number (Figure 3D,E) 31

1. Computing the Condition Number κ 31

2. Estimating the minimal condition number κ 32

G. Applicability of ϕ and κ in Empirical Tests 33

1. Test 1: Assembly from Pairwise-Coexisting Species 33

2. Test 2: Predictability Under Measurement Error 34

H. Stability and feedback loops 35

1. Random-Matrix Stability Bounds 35
2. Distance to the Stability Boundary 35
3. Routh–Hurwitz Stability Analysis 36
4. Stability Conditions for Small Communities 37
I. Measuring competitive hierarchies and intransitivity (Figure 4) 38
1. Competitive ranks and Low Rank Exclusion 38
2. Competitive triplets and Rock-Paper-Scissors 39
3. Other metrics of intransitivity 39
4. Building Figure 4 39
5. Low-rank exclusions require four exclusions and remain rare 40
6. Rock–paper–scissors loops converge to the random expectation 41
7. Equivalent results using the Laird–Schamp index 41
J. On the role of heterogeneous growth rates 42
K. On the role of migration and reinvasions 44

Figures 45

References 64

Legend of Figures 67

I. METHODS, SIMULATIONS AND MODELS

A. Description of the problem and the GLV model

The central question of this work is: *Can a species-rich community model with pairwise interactions generate stable states in which some species pairs do not coexist in co-culture?*

This question is motivated by recent findings in multispecies microbial communities showing that stable communities can include species pairs that exclude each other in isolation due to strong competition (Chang *et al.*, 2023; Friedman *et al.*, 2017; Higgins *et al.*, 2017; Lele *et al.*, 2024; Venturelli *et al.*, 2018). Because community coexistence would then not be expected from pairwise observations, this phenomenon has been termed *Emergent Coexistence* (EC) (Chang *et al.*, 2023).

From this, two additional questions arise: *(i)* If a pairwise interaction model can generate EC, what mechanisms allow these excluding pairs to coexist within a community? and *(ii)* Can we reliably infer coexistence and community composition from pairwise data alone?

To address these questions, we study a family of community models with pairwise interactions, and focus our analysis on the generalized Lotka–Volterra (GLV) model, the simplest framework for multispecies pairwise interactions, which has recently received considerable attention (Aguadé-Gorgorió and Kefi, 2024; Altieri *et al.*, 2021; Barbier *et al.*, 2018; Bunin, 2017; Galla, 2018; Kessler and Shnerb, 2015; Mallmin *et al.*, 2024; Serván *et al.*, 2018; Zelnik *et al.*, 2024). In one of several formulations of the GLV model, the abundance of species i, denoted N_i, evolves in time as

$$\frac{dN_i}{dt} = r_i N_i \left(1 - \frac{N_i + \sum_{j \neq i}^{S} a_{ij} N_j}{K_i}\right), \tag{1}$$

where r_i is the intrinsic growth rate, K_i the carrying capacity, and a_{ij} an interaction term describing the effect of species j on species i.

Following (Aguadé-Gorgorió and Kefi, 2024; Leibold and Barbier, 2025; Mallmin *et al.*, 2024; Zelnik *et al.*, 2024), it is useful to rescale parameters to focus on the role of interactions. We define the relative yield $x_i = N_i/K_i$ and a normalized interaction strength $A_{ij} = a_{ij}K_j/K_i$, which quantifies interspecific effects relative to self-regulation strengths. The distinction and comparison of inter- and intra-species interaction strengths is a central topic in theoretical ecology (Barabás *et al.*, 2017; Hatton *et al.*, 2024).

In our notation following (Zelnik *et al.*, 2024), the matrix A contains only off-diagonal (interspecific) terms, while diagonal elements are zero. The complete interaction matrix including self-regulation is therefore $-I + A$, where I is the identity matrix. This convention has no direct implications but simplifies later analysis when inverting $(I - A)^{-1}$ and using the Neumann series.

The GLV model in terms of relative yields becomes

$$\frac{dx_i}{dt} = r_i x_i \left(1 - x_i + \sum_{j \neq i}^{S} A_{ij} x_j\right). \tag{2}$$

The key question is whether this model can produce stable multispecies communities where some pairs fail to coexist in isolation. If not, higher-order effects (Billick and Case, 1994; Gallien *et al.*, 2017; Grilli *et al.*, 2017b) may be required to explain EC.

To further isolate the role of interspecific interactions, we assume here homogeneous growth rates ($r_i = r$), allowing rescaling of time ($t \to rt$) (Mallmin *et al.*, 2024; Zelnik *et al.*, 2024). The model then simplifies to

$$\frac{dx_i}{dt} = x_i \left(1 - x_i + \sum_{j \neq i}^{S} A_{ij} x_j\right). \tag{3}$$

In the main text we present results for homogeneous growth rates to emphasize the role played by interactions, while we discuss in Section II.J the effects of non-homogeneous growth ($r_i \neq r$) as well as in Section I.F, II.A the impacts that heterogeneous carrying capacities can have on matrix structure given the normalization $A_{ij} = a_{ij} K_j / K_i$.

B. Pairwise exclusions, equilibria, net interactions and indirect effects

1. Pairwise exclusions

A classical problem in theoretical ecology is determining when two interacting species coexist or exclude each other in isolation (Chesson, 2000; Strogatz, 2018).

Consider the system:

$$\frac{dx_1}{dt} = x_1(1 - x_1 + A_{12} x_2), \tag{4}$$

$$\frac{dx_2}{dt} = x_2(1 - x_2 + A_{21} x_1). \tag{5}$$

At equilibrium (x_1^*, x_2^*), the Jacobian is

$$J = \begin{pmatrix} 1 - 2x_1^* + A_{12} x_2^* & x_1^* A_{12} \\ x_2^* A_{21} & 1 - 2x_2^* + A_{21} x_1^* \end{pmatrix}. \tag{6}$$

The exclusion fixed points are $(1, 0)$ and $(0, 1)$. For $(1, 0)$, eigenvalues solve

$$\det(J - \lambda I) = -(1 + \lambda)(1 + A_{21} - \lambda) = 0, \tag{7}$$

giving $\lambda_1 = -1$ and $\lambda_2 = 1 + A_{21}$. Stability requires

$$A_{21} < -1, \tag{8}$$

meaning species 1 excludes species 2 if interspecies competition exceeds self-regulation.

Similarly, species 2 excludes species 1 if

$$A_{12} < -1. \tag{9}$$

Coexistence occurs if both

$$A_{12} > -1, \quad A_{21} > -1, \tag{10}$$

while bistable exclusion happens if both are less than -1 (Aguadé-Gorgorió *et al.*, 2024; Strogatz, 2018). This frames the question: can we find stable coexistence matrices A^* in the GLV model, even if some species pairs, called *excluding pairs*, fail to coexist in isolation due to strong competition ($A^*_{ij} < -1$)?

2. Indirect effects in three-species toy models

As argued in the main text, strong indirect effects alone can generate stable communities with excluding pairs and thus emergent coexistence (EC). Intransitivity or higher-order effects may occur but are not necessary to recover the empirical patterns of (Chang *et al.*, 2023).

Simple few-species Lotka-Volterra models help illustrate this link (Bender *et al.*, 1984; Dambacher *et al.*, 2003; Levine, 1999, 1976; Levins, 1974; Strauss, 1991; Vandermeer, 1980; Wootton, 1994; Yodzis, 1988), with a comprehensive discussion of indirect effects, intransitivity, and higher-order interactions in (Levine *et al.*, 2017). Consider a minimal three-species case: species 2 excludes 1 ($A_{12} < -1$), species 1 does not affect 2, and species 3 competes with both but is unaffected by them. This yields a perfectly nested, triangular interaction matrix A, as a toy model of triangular matrices T explored in the main text. The dynamics follow:

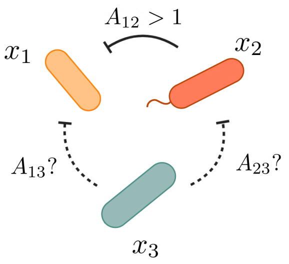

$$A = \begin{pmatrix} 0 & A_{12} & A_{13} \\ 0 & 0 & A_{23} \\ 0 & 0 & 0 \end{pmatrix}$$

$$\frac{dx_1}{dt} = x_1\left(1 - x_1 - A_{12}x_2 - A_{13}x_3\right)$$

$$\frac{dx_2}{dt} = x_2\left(1 - x_2 - A_{23}x_3\right)$$

$$\frac{dx_3}{dt} = x_3\left(1 - x_3\right)$$

Here, A_{ij} are positive for competition (note the reversed sign convention for clarity). The equilibrium abundances are:

$$x_1^* = 1 - A_{13} - A_{12} + A_{12}A_{23}, \quad x_2^* = 1 - A_{23}, \quad x_3^* = 1. \tag{11}$$

From where the positive indirect effect between competitors can be easily seen, $+A_{12}A_{23}$. Species 3 rescues species 1 if all abundances are positive, requiring

$$A_{23} < 1, \quad A_{13} < 1 - A_{12}(1 - A_{23}). \tag{12}$$

Feasibility (Grilli *et al.*, 2017a; Marcus *et al.*, 2024) thus demands $A_{23} \lesssim 1$ (to limit species 2) and $A_{13} \gtrsim 0$ (to avoid excluding species 1). Stronger A_{12} increases pressure on species 1, forcing more heterogeneity between A_{13} and A_{23}. Assuming $A_{13}, A_{23} \sim \mathcal{U}(c-w, c+w)$, it is easy to prove that coexistence requires

$$w > (1 - c)\frac{A_{12} + 1}{A_{12} - 1}. \tag{13}$$

Hence, interaction heterogeneity w, the width of the uniform distribution in this example, is essential for emergent coexistence, as it enables the right weight of positive indirect effects to counteract direct competition. This minimal example has no intransitivity or complex motifs, only a triangular matrix and linear chains of direct and indirect effects.

Weak competitive links, often dismissed as negligible (Chang *et al.*, 2023), are crucial as they form the backbone of indirect effect chains stabilizing communities (Neutel *et al.*, 2002). This also explains why previous models of binary tournament matrices miss the nuances shown in this toy model (Allesina and Levine, 2011; Laird and Schamp, 2006).

Although half of the interaction network is sparse here, increasing species number and connectivity quickly expands indirect interaction pathways. Recent frameworks now enable characterization of indirect effects in such high-dimensional systems (Zelnik *et al.*, 2024).

3. Equilibria, net interactions and the Jacobian matrix

The GLV model offers a simple description of communities based on pairwise interactions. Its bilinear terms, $x_i A_{ij} x_j$, reduce equilibrium finding to a linear problem:

$$\frac{dx_i}{dt} = r_i x_i \left(1 - x_i + \sum_{j \neq i} A_{ij} x_j\right). \tag{14}$$

At equilibrium, some species have abundance $x_i^* = 0$ (extinction), while other species might fulfill

$$1 - x_i + \sum_{j \neq i} A_{ij} x_j = 0, \tag{15}$$

which in vector form is

$$(I - A)\mathbf{x} = \mathbf{1}, \tag{16}$$

where A has zero diagonal (self-regulation is in I). Growth rates r_i do not affect coexistence conditions, as we review in Section II.J.

Not all S species survive; stable states exist for subsets $S^* \leq S$ with interactions A^*, where A^* is the reduced matrix containing only the interactions between surviving species, with abundances

$$\mathbf{x}^* = (I - A^*)^{-1}\mathbf{1}, \tag{17}$$

and for each species

$$x_i^* = \sum_j (I - A^*)^{-1}_{ij}. \tag{18}$$

Coexistence requires all these to be positive (Bunin, 2017). This framework easily captures how extinction and coexistence emerge (Liautaud *et al.*, 2019; Mallmin *et al.*, 2024). The matrix $(I - A^*)^{-1}$ represents net effects, i.e., long-term abundance changes in species i due to changes in species j's growth rate, and therefore each element of $(I - A^*)^{-1}$ encodes the *true* or *complete* effect of j on i in the context of the full community (Arnoldi *et al.*, 2022; Levine, 1976; Liautaud *et al.*, 2019; Strauss, 1991; Wootton, 1994; Zelnik *et al.*, 2024). In light of the results of (Chang *et al.*, 2023), the central question is to study the relation between A^*_{ij}, the direct pairwise effects, and $(I - A^*)^{-1}_{ij}$, the effects in the context of the full community. These two elements are not necessarily equivalent nor, as we shown, always correlated.

Empirically, A^* can be estimated by pairwise growth comparisons (Pennekamp *et al.*, 2018; Venturelli *et al.*, 2018). However, community equilibrium depends on net effects, not just direct interactions. Using Neumann's series,

$$(I - A^*)^{-1} = I + A^* + (A^*)^2 + (A^*)^3 + \cdots, \tag{19}$$

which implies

$$x_i^* = 1 + \sum_j A^*_{ij} + \sum_{j,k} A^*_{ik} A^*_{kj} + \sum_{j,k,l} A^*_{ik} A^*_{kl} A^*_{lj} + \cdots. \tag{20}$$

Thus, x_i^* depends not only on direct neighbors but on all indirect interaction chains, including feedback loops, capturing the essence of indirect effects in complex communities. More importantly, we have no a priori evidence that A^* is more important than, for example, $(A^*)^3$, in determining the final state of the community.

The Jacobian matrix at equilibrium has entries

$$J_{ij} = r_i x_i^* A_{ij} \quad (i \neq j), \quad J_{ii} = -r_i x_i^*, \tag{21}$$

or compactly

$$J = \text{diag}(r_i x_i^*)(-I + A^*). \tag{22}$$

Stability is assessed by eigenvalues of J; all negative eigenvalues indicate recovery from perturbations (Allesina and Tang, 2012; May, 1972). We focus on this linear stability measure and refer to more detailed discussions of ecological stability and resilience in (Domínguez-García *et al.*, 2019; Donohue *et al.*, 2016; Kéfi *et al.*, 2019; Lajaaiti *et al.*, 2024).

C. Finding stable communities

Building on the mathematical framework described above, we use two complementary numerical methods to find stable equilibrium states in GLV communities, based solely on the mean and heterogeneity of interaction strengths (μ, σ). These approaches were developed and detailed in our previous studies (Aguadé-Gorgorió *et al.*, 2024; Aguadé-Gorgorió and Kefi, 2024) and are common methods throughout the literature. Different matrices and parametrizations are described in following sections.

1. Simulating ecological dynamics

To find equilibrium states of the GLV model (Eq. 2), we numerically integrate the system for different values of μ and σ, following different matrix randomizations as proposed below. For Fig. 2A in the main text, these parameters are varied gradually within the mentioned range. The interaction matrix A in the main text is generated such that each element A_{ij} is drawn from $\mathcal{N}(\mu, \sigma)$ with probability C, or set to zero with probability $1 - C$, and discuss below a large set of variations to this uncorrelated matrix method. Initial conditions are randomly chosen as $x_i(t = 0) \in \mathcal{U}[0, 1)$. Following (Aguadé-Gorgorió *et al.*, 2024; Aguadé-Gorgorió and Kefi, 2024), the equations are integrated up to $t = 3000$ using a fifth-order Runge-Kutta method (Dormand and Prince, 1980), ensuring the system reaches beyond typical relaxation times.

Negative abundances, which have no ecological meaning, are treated differently depending on whether the community is open or closed. In open systems, extinction can be avoided by including migration or a nonzero persistence threshold (see Section II.K and (Aguadé-Gorgorió and Kefi, 2024; Roy *et al.*, 2020)). Here, however, we focus on closed systems such as laboratory microbial consortia (Chang *et al.*, 2023; Friedman *et al.*, 2017), where extinct species cannot reinvade. We therefore impose a minimal abundance threshold of 10^{-20}: any species falling below it is set to $x_i = 0$ and removed from further dynamics (Aguadé-Gorgorió *et al.*, 2025).

To assess whether the system reaches a stationary state, we test whether abundances remain constant over an additional time window $\Delta t = 100$. If any species changes by more than $|x_i(t = 3100) - x_i(t = 3000)| > 10^{-3}$, the state is considered unstable (Aguadé-Gorgorió *et al.*, 2024; Aguadé-Gorgorió and Kefi, 2024). Otherwise, the system is deemed stationary, and we record the surviving species' abundances $\boldsymbol{x}^*$ and their corresponding interaction submatrix A^*. Extinct species are removed from both $\boldsymbol{x}^*$ and A, and we record a reduced community representation $(\boldsymbol{x}^*, A^*)$ used for subsequent analyses of stationary coexistence and interaction structure.

2. Sampling and testing random species subsets

The numerical integration method described above becomes computationally expensive when repeated extensively, such as in the analysis of intransitivity statistics for which we require finding many communities with EC. To address this, we also apply an analytical approach developed in (Aguadé-Gorgorió and Kefi, 2024), which directly computes the stationary abundances $\boldsymbol{x}^*$ and interaction matrices A^* by exploiting the linearity and simplicity of the GLV model. We refer the reader to (Aguadé-Gorgorió and Kefi, 2024) for a detailed comparison of the sampling method with the communities arising from integrating the complete dynamical equations.

Given an interaction matrix $A \in \mathcal{N}(\mu, \sigma)$, we select a subset of S^* species (randomly or stepwise between 3 and S, depending on the analysis). Because emergent coexistence requires at least three species, pairs are excluded from the search, whereas we know from figure 2C in the main text that communities with many more species and EC become rarer and harder to find. The subcommunity matrix A^* is obtained by keeping only the rows and columns of A corresponding to the selected species (Friedman *et al.*, 2017).

Stationary abundances are computed analytically as $\boldsymbol{x}^* = (I - A^*)^{-1}\mathbf{1}$. Subsets are considered

feasible when all these abundances are positive, or are discarded if not. Local stability for the feasible subsets is then tested by evaluating the Jacobian matrix, $J_{ij} = x_i^* A_{ij}^*$ for $i \neq j$ and $J_{ii} = -x_i^*$, and verifying that all eigenvalues have negative real parts (`numpy.linalg.eigvals`). Subsets satisfying both criteria are stored as stable equilibria defined by $\boldsymbol{x}^*$ and A^*.

This analytical sampling method and the dynamic integration approach are mathematically equivalent in the absence of migrations and provided that some initial conditions are set to zero, meaning that some species are not present initially. Instead, the dynamic method starts from all species presents and allows extinctions to occur naturally ("random zoo" scenario (Chang *et al.*, 2023; Serván *et al.*, 2018)), while the subset method tests predefined species combinations, akin to microbial co-culture experiments (Friedman *et al.*, 2017; Venturelli *et al.*, 2018). Both approaches converge when the initial conditions in the dynamic method include only a subset of species and extinct species cannot reinvade. If not, the sampling method sometimes finds small communities that fulfill the coexistence and stability criteria, yet would not be found in the dynamics if starting from all possible species, because some species could be able to invade the subset. As shown in (Aguadé-Gorgorió and Kefi, 2024), this implies that communities sampled with the subset method sometimes harbor slightly stronger competition than those sampled from the dynamics, yet the overall properties of communities sampled from both methods remain the same. This is discussed mathematically in Section II.A.5.

D. Species interactions from random distributions and disordered models in ecology

In our formulation, GLV dynamics depend on the off-diagonal elements of the interaction matrix A and on species growth rates r_i and carrying capacities K_i. Estimating all A_{ij} from time-series data is challenging, as it requires fitting $S(S-1)$ parameters (Picot *et al.*, 2023; Rosenbaum and Fronhofer, 2023), and multiple parameter sets can reproduce the same dynamics (Lubiana Botelho *et al.*, 2025). Consequently, quantitative inference is often unreliable, yet qualitative predictions can be obtained by assuming that interactions are random variables with known aggregate statistics. This disordered systems approach focuses on the mean and variance of A, which characterize community-level properties without specifying individual interactions (Aguadé-Gorgorió *et al.*, 2024; Barbier *et al.*, 2018, 2021; Bunin, 2017; May, 1972). We refer the reader to recent works discussing the meaning and implications of the disordered method in community ecology (Barbier, 2025; Leibold and Barbier, 2025).

Because most EC experiments lack empirical estimates of interaction strengths (Chang *et al.*, 2023; Friedman *et al.*, 2017) (see (Venturelli *et al.*, 2018) for the exception in our work), random interactions provide a natural baseline for exploring coexistence. Rather than reconstructing detailed ecological networks such as food webs (Dunne, 2006) or mutualisms (Bascompte, 2009; Payrató-Borras *et al.*, 2019; Suweis *et al.*, 2013), we ask whether a minimal community model can reproduce emergent coexistence without imposing structure on A, or else if specific non-random features are required (Barbier *et al.*, 2021; Kessler and Shnerb, 2025). This approach also allows testing whether recurrent patterns, such as Rock-Paper-Scissors motifs, arise spontaneously from random interactions or reflect ecological selection (Hutchinson, 1953).

Following (Altieri *et al.*, 2021; Bunin, 2017), interspecies interactions A_{ij} are sampled from a Gaussian distribution with mean μ and standard deviation σ, yet now without rescaling by

community size (Aguadé-Gorgorió *et al.*, 2024; Aguadé-Gorgorió and Kefi, 2024; Mallmin *et al.*, 2024). This two-parameter model defines a phase space (μ, σ) capturing different dynamical regimes (Aguadé-Gorgorió and Kefi, 2024). In Fig. 2A, we explore $\mu \in [-2.0, 0.5]$ and $\sigma \in [0, 1]$, spanning from strong competition to weak cooperation. Self-regulation is implemented as an identity matrix, ensuring that randomness acts only through interspecific terms (Zelnik *et al.*, 2024).

E. Network structure and connectivity in microbial communities

Interaction strengths can be separated from network structure by expressing them as the element-wise product of an adjacency matrix M and a strength matrix A (Grilli *et al.*, 2016; Poley *et al.*, 2025). We denote this product $A \circ M$ simply as A, since M plays a secondary role in our work. Ecological networks display diverse architectures: mutualistic networks are typically nested, while food webs tend to be modular (Bascompte, 2010; Fortuna *et al.*, 2010; Guimaraes Jr, 2020). Such structures may promote robustness (Landi *et al.*, 2018) or emerge passively from assembly or heterogeneity (Payrató-Borras *et al.*, 2019; Valverde *et al.*, 2018), and their ecological significance remains debated (Barbier *et al.*, 2018).

In contrast, microbial interaction networks are often sparse and lack clear large-scale structure (Arya *et al.*, 2023; Camacho-Mateu *et al.*, 2024; Venturelli *et al.*, 2018), although specific features may depend on microbiome type and resource context (Dal Bello *et al.*, 2021; Estrela *et al.*, 2022). Following recent evidence, we model sparsity as the only defining property of microbial networks (Arya *et al.*, 2023; Camacho-Mateu *et al.*, 2024). Sparsity corresponds to many zero entries in M, representing unrealized interactions and simplifying both computation and interpretation (Giral Martínez *et al.*, 2024; Hutchinson, 1953).

We model M as an Erdős-Rényi graph with connection probability $p = C$ (Newman, 2018; Van Der Hofstad, 2024). Interaction strengths are sampled from a Gaussian distribution and masked by M. Unless otherwise stated, we assume full connectivity ($C = 1$), and study lower C values in Section II.A,B. Empirical estimates range from dense ($C \approx 0.77 - 0.9$) (Koch *et al.*, 2023; Venturelli *et al.*, 2018) to very sparse networks (Arya *et al.*, 2023; Camacho-Mateu *et al.*, 2024). Laboratory systems may be typically dense due to limited niche diversity (Chang *et al.*, 2023; Friedman *et al.*, 2017), whereas natural environments may support sparser, weaker interactions. Reducing connectivity decreases effective competition and alters heterogeneity, influencing coexistence patterns (Aguadé-Gorgorió and Kefi, 2024). However, qualitative results on emergent coexistence and indirect effects remain robust across connectivity values (Sections II.A.6 and II.B.3).

F. The GLV model with other interaction matrices

We extend the Gaussian random interaction matrices A_{ij} of the main text by testing additional structures commonly studied in ecological theory (Allesina and Tang, 2012; Bunin, 2017; Grilli *et al.*, 2017a, 2016). We construct **(1)** symmetric ($A_{ij} = A_{ji}$) and **(2)** antisymmetric ($A_{ij} = -A_{ji}$) matrices by sampling the upper triangle from a normal distribution and filling the lower triangle accordingly. We introduce two additional modifications: **(3)** *nestedness* and **(4)** *connectivity.*

Nestedness is set by a parameter p, the probability that $A_{ij} = 0$ for $i > j$, producing triangular (hierarchical) matrices at high p. Unless stated otherwise, we study $p = 1$, a fully triangular matrix, in the main text, and weaker versions of $p = 0.75$ when specified in the figure. Connectivity is tuned by setting each off-diagonal element to zero *independently* with probability $1 - C$ following the method of Erdős-Rényi graphs. We explore **(5)** skewed competition generated by sampling A_{ij} from a Gamma distribution instead of a Gaussian distribution (Koch *et al.*, 2024) by using `numpy.random.gamma`, which creates a typical pattern of many weak and few strong competitors.

We also study **(6)** how our regularization method ($A_{ij} = a_{ij}K_j/K_i$) could introduce correlations between interaction strength and carrying capacities K_i. Such correlations have been shown to affect extinction cascades and increase competitive hierarchy (Pearl Mizrahi *et al.*, 2025), and we study how heterogeneous carrying capacities affect the presence of EC and competitive hierarchies. We generate K_i uniformly from $\mathcal{U}[0.5, 1.5]$, a_{ij} from $\mathcal{N}(\mu, \sigma)$ as done for A in the main text, and define a new correlated interaction matrix $\tilde{A}$ (to differentiate from A in the main text) as $\tilde{A}_{ij} = a_{ij}K_j/K_i$.

We finally explore **(7)** a structured matrix B that includes strong single-resource competition, additional weak random competition, and cross-feeding. To mimic single-resource competition, we assign each species a trait γ_i representing uptake rate (Lee *et al.*, 2023). Interactions are first defined as

$$B_{ij} = -\frac{2\gamma_j}{\gamma_i + \gamma_j}. \tag{23}$$

When $\gamma_i \approx \gamma_j$, competition is symmetric ($B_{ij} \approx -1$), while large trait differences create asymmetric exclusion. We draw $\gamma_i \in \mathcal{U}[0.3, 0.7]$ and order species by γ_i, producing a nested, hierarchical structure.

To relax this perfect hierarchy, we add weak random effects $a_{ij} \sim \mathcal{N}(0.1, 0.01)$:

$$B_{ij} = -\frac{2\gamma_j}{\gamma_i + \gamma_j} - a_{ij}. \tag{24}$$

If $a_{ij} = 0$, the matrix is fully transitive with no RPS loops. Increasing randomness introduces intransitivity and coexistence variability, in a way that is similar to the $A_{ij} = a_{ij}K_j/K_i$ method, that holds some hierarchy from K_i and some randomness from a_{ij}.

We finally include *cross-feeding*, where species benefit from byproducts of stronger competitors (Estrela *et al.*, 2022; Goldford *et al.*, 2018). These positive interactions are introduced via a matrix $C_{ij} \sim \mathcal{U}[0, 1]$, giving

$$B = C_{ij} - \frac{2\gamma_j}{\gamma_i + \gamma_j} - a_{ij}. \tag{25}$$

This matrix balances hierarchical competition, weak randomness, and cooperative effects. Positive terms remain small to prevent unbounded growth in the GLV framework. Although parameters are heuristic, this formulation captures how hierarchical competition combined with weak cross-feeding can yield emergent coexistence under transitive structures (Aguadé-Gorgorió *et al.*, 2024; van den Berg *et al.*, 2022; Mehta and Marsland III, 2021; Qian and Akçay, 2020), in a way that is similar to row and column correlations emerging from carrying capacities (Pearl Mizrahi *et al.*, 2025).

These different random matrices serve as a null model when real interaction data are unavailable (Barbier *et al.*, 2018; Giral Martínez *et al.*, 2024; Martínez *et al.*, 2024). Our goal is not to

reproduce empirical matrices but to identify general qualitative trends. Since inferring A_{ij} experimentally remains challenging (Lubiana Botelho *et al.*, 2025; Picot *et al.*, 2023; Rosenbaum and Fronhofer, 2023), applying this framework to real data is left for future work. Relevant microbial datasets include (Arya *et al.*, 2023; Camacho-Mateu *et al.*, 2024; Castledine *et al.*, 2024; Ortiz *et al.*, 2021; Pennekamp *et al.*, 2018; Rao *et al.*, 2021; Schmitz *et al.*, 2024; Venturelli *et al.*, 2018). Similar questions have been explored in plant communities (Dormann, 2007; Dormann and Roxburgh, 2005; Engel and Weltzin, 2008; Roxburgh and Wilson, 2000), though weaker interactions and slower dynamics likely limit their collective effects.

G. Other dynamical models with pairwise interactions

A central goal of this work is to show that Emergent Coexistence (EC) can arise from simple models of pairwise interactions. We use the Generalized Lotka–Volterra (GLV) framework with different matrix randomizations as a minimal, well-characterized system for analyzing community dynamics under strong competition and dense indirect effects (Altieri *et al.*, 2021; Bunin, 2017; Galla, 2018; Hu *et al.*, 2022; Kessler and Shnerb, 2015; Mallmin *et al.*, 2024; Pasqualini *et al.*, 2024). Its simplicity enables analytical measures such as the collectivity metric (Zelnik *et al.*, 2024) and exclusion tests via $A_{ij} < -1$.

We extend this analysis to other pairwise models lacking such simple analytical criteria. For each model, we numerically identify EC by constructing a numerically-inferred tournament matrix as if replicating the experimental setup of (Chang *et al.*, 2023). Starting from S species with random interactions, we simulate dynamics until a stable state with S^* survivors is reached, then perform pairwise simulations among the S^* coexisting species. Each pair starts at $x_i = 1$, and exclusion in any pair indicates EC. Because this procedure is computationally demanding, we use a pool of size $S = 30$ instead of $S = 80$ as in the GLV analysis, focusing on qualitative patterns of stable diversity. These complementary models test whether EC persists under more complex interaction forms and growth laws.

Section II.A.5 presents EC analyses for all models.

1. Saturating interactions with a Holling response

The GLV model allows unbounded growth when $\mu > 0$. To include saturating effects and avoid extreme cooperation or competition, we apply a Holling type II response (Castillo-Alvino and Marvá, 2020; Holling, 1959; Kvrivan and Eisner, 2006; Yu *et al.*, 2019):

$$\frac{dx_i}{dt} = x_i \left(1 + \sum_{j=1}^{S} A_{ij} \frac{x_j}{1 + x_j} \right), \tag{26}$$

where A_{ij} are generated as in the GLV case with mean μ and variance σ. Self-regulation is included in the sum, which does not change qualitative outcomes. Saturation prevents divergence

of cooperative terms and caps competitive effects, limiting dominance by a single species. Section II.A.5 reports the fraction of communities displaying EC across (μ, σ) space with this model.

2. Multilayer interactions with Allee Effects

To represent multiple interaction types, we extend the model by separating facilitation and competition into distinct matrices A and B (Aguadé-Gorgorió *et al.*, 2024; Pilosof *et al.*, 2017), and introduce an Allee effect through a minimal abundance threshold (Courchamp *et al.*, 2008). The dynamics are:

$$\frac{dx_i}{dt} = x_i \left(\sum_{j=1}^{S} A_{ij} \frac{x_j}{1 + x_j} - d_i - \sum_{j=1}^{S} B_{ij} x_j \right), \tag{27}$$

where A and B are positive matrices representing saturating facilitation and linear competition, and d_i is a positive death rate generating an Allee effect in isolation. Following the in-depth analysis of (Aguadé-Gorgorió *et al.*, 2024), matrices are drawn from log-normal distributions (`numpy.random.lognormal`) with $\mu_d = 0.1$, $\sigma_d = \log(1.1)$, and $\sigma_A = \sigma_B = \log(1.1)$; the means μ_A and μ_B are varied to explore the phase space. This framework captures richer coexistence mechanisms while maintaining pairwise structure (see (Aguadé-Gorgorió *et al.*, 2024) for an analysis of the multistability landscape of this model).

3. Sublinear growth dynamics coupled to linear interactions

Finally, we test whether EC persists when growth itself deviates from linearity. Sublinear replication has been proposed for prebiotic and ecological systems (Czárán and Szathmáry, 2000; Hatton *et al.*, 2024; Piñero and Solé, 2018; Szathmáry and Smith, 1997). The model is

$$\frac{dN_i}{dt} = r_i N_i^k - d_i N_i - N_i \sum_{j \neq i}^{S} A_{ij} N_j, \tag{28}$$

where $0 < k < 1$. Following (Aguadé-Gorgorió *et al.*, 2025; Hatton *et al.*, 2024), we simulate absolute abundances N_i rather than relative yields x_i. In this regime, species cannot reach zero abundance and thus never go extinct (Aguadé-Gorgorió *et al.*, 2025; Szathmáry and Gladkih, 1989). To restore realistic exclusion, we define extinction at $N_i < 1$ following the analysis of (Aguadé-Gorgorió *et al.*, 2025).

We fix $r_i = 1$, $d_i = 0.05$, and generate A_{ij} as in previous models. Section II.A.5 presents EC results for this model across (μ, σ) parameter space. Together, these analyses test the robustness of emergent coexistence across interaction types and nonlinear growth dynamics.

II. IMPLEMENTATION AND RESULTS

A. Emergent coexistence across the parameter space (Figure 2A)

After obtaining a stable state with positive abundances $\boldsymbol{x}^*$ and its associated interaction matrix A^* following the numerical methods described above, we test whether the state exhibits emergent coexistence (EC). A state is classified as showing EC when at least one pairwise interaction would lead to competitive exclusion in co-culture. Specifically, for each species pair $i \neq j$, we identified whether $A^*_{ij} < -1$ or $A^*_{ji} < -1$. Any such inequality indicates that, in isolation from the rest of the community, species j would exclude i, species i would exclude j, or the pair would exhibit bistable exclusion. For now we do not distinguish among these cases; we only record whether the pair fails to coexist in co-culture following (Chang *et al.*, 2023), and leave for future work the detailed analysis of the role of bistable exclusion.

We then quantified how frequently EC occurs across parameter values. For each (μ, σ) pair, we generated 100 systems, each defined with random initial conditions and randomly sampled elements of the interaction matrix $A \sim \mathcal{N}(\mu, \sigma)$. Among all stable states obtained that contain at least three coexisting species (count n), we counted how many contained at least one excluding pair (count n_{EC}). The requirement of three coexisting species is essential to capture the possibility of observing EC states. This is because EC states require at least three coexisting species: stable states with one survivor do not harbor interactions, and stable states with two survivors require that interactions are not exclusionary and hence EC is impossible. If we just compare the number of EC states against all possible stable states, we see very low numbers, indicating not that EC is very rare, but that we are sampling μ, σ regimes where species coexistence is very rare (see Fig. A). Because we want to focus on the former (the ubiquity of EC states provided coexistence is achieved), we include the “minimum three species” requirement. Figure 2A in the main text reports the ratio n_{EC}/n across a broad parameter range. EC is absent in three regions of the GLV parameter space (growth, exclusion and unique fixed point phases, see (Aguadé-Gorgorió *et al.*, 2024; Bunin, 2017)), but becomes frequent in an “Emergent Coexistence Regime,” where roughly 80% of stable states contain at least one excluding pair (Fig. 2B of the main text).

1. The minimal competition necessary for emergent coexistence

Emergent Coexistence (EC) requires that at least one interaction among surviving species is exclusionary, i.e. $A^*_{ij} < -1$ for some $i \neq j$. A necessary condition is therefore that the full interaction pool A already contains at least one exclusionary element. This is of course not a sufficient condition, and serves only as a first step: if all $A_{ij} \geq -1$, EC is impossible, yet if some $A_{ij} < -1$, this does not automatically imply that these excluding elements will carry from the pool A to the final stable community A^*, only that EC is possible.

Consider a pool of S species with $S(S-1)$ interspecific interactions, each independently drawn from a normal distribution:

$$A_{ij} \sim \mathcal{N}(\mu, \sigma), \qquad i \neq j. \tag{29}$$

We compute the probability that at least one interaction is exclusionary:

$$P(\exists\, A_{ij} < -1) = 1 - P(A_{ij} \geq -1 \text{ for all } i \neq j). \tag{30}$$

Independence between all interaction elements yields

$$P(\exists\, A_{ij} < -1) = 1 - (1 - P(A_{ij} < -1))^{S(S-1)}\,, \tag{31}$$

meaning that observing at least one exclusionary element is the complement of all interaction coefficients being larger than -1. For a single element,

$$P(A_{ij} < -1) = \Phi\left(\frac{-1-\mu}{\sigma}\right), \tag{32}$$

where Φ is the standard normal cumulative distribution function. Thus,

$$P(\exists\, A_{ij} < -1) = 1 - \left[1 - \Phi\left(\frac{-1-\mu}{\sigma}\right)\right]^{S(S-1)}. \tag{33}$$

For large S, this probability becomes an increasingly sharp step function in σ for fixed μ, with the transition occurring at progressively smaller values of σ. For example, with $S = 100$ ($S(S-1) = 9900$), a critical value σ_1^c separates a region where exclusionary interactions are almost surely absent from one where they are almost surely present. This defines the lower boundary of the EC region in the (μ, σ) phase space: below σ_1^c, all interactions are pairwise coexisting, whereas above this boundary exclusionary elements exist in the pool. However, not all feasible and stable submatrices A^* necessarily inherit these exclusionary interactions, so the fraction of EC states increases gradually rather than abruptly beyond σ_1^c (Fig. 2B).

This condition is necessary but not sufficient. In structured interaction matrices, such as symmetric A_{ij}, exclusionary entries may be present in the interaction pool while no feasible and stable multispecies communities survive; in such cases EC does not occur (see *Emergent coexistence under other interaction matrices*).

To estimate σ_1^c for each μ and draw the boundary, we solve numerically

$$P(\exists\, A_{ij} < -1) = 0.5 \tag{34}$$

using a bisection root-finding method. We use `scipy.optimize.bisect` over $\sigma \in [0.01, 1]$, which ensures convergence and numerical stability.

To make explicit how this boundary depends on system size, one may define σ_1^c implicitly by fixing a reference probability, for instance

$$P(\exists\, A_{ij} < -1) = \tfrac{1}{2}. \tag{35}$$

This yields

$$\Phi\left(\frac{-1-\mu}{\sigma_1^c}\right) = 1 - 2^{-1/[S(S-1)]}. \tag{36}$$

For large S, the right-hand side satisfies

$$1 - 2^{-1/[S(S-1)]} \simeq \frac{\ln 2}{S(S-1)}, \tag{37}$$

implying

$$\frac{-1-\mu}{\sigma_1^c} \sim -\sqrt{2\ln[S(S-1)]}, \qquad \sigma_1^c \sim \frac{1+\mu}{\sqrt{2\ln[S(S-1)]}}. \tag{38}$$

Thus, although the location of the boundary continues to shift with increasing S, it does so only logarithmically slowly. As a result, over the finite range of system sizes explored numerically this boundary appears only weakly dependent on S, even though it does not converge to a finite limit for asymptotically large systems. This slow scaling contrasts with the much faster displacement of the multistability boundary with S, explaining why EC is most frequently observed within the multistability domain (Fig. S1).

2. The EC boundary and multistability

The EC boundary found above differs from the classical transition between monostability and multistability in random GLV systems (Bunin, 2017; May, 1972). Our line σ_1^c captures the probability that A has *at least* one exclusionary interaction, whereas the multistability boundary (dashed line in Fig. 2A) is derived from dynamical mean-field theory (Aguadé-Gorgorió *et al.*, 2024; Bunin, 2017). The dashed line in Fig. 2A of the main text is computed using the formalism in (Aguadé-Gorgorió and Kefi, 2024; Mallmin *et al.*, 2024).

It is interesting to understand if the EC region lies entirely within the multistable regime, and how does this change if S^* changes. The multistability regime is crossed if interaction heterogeneity σ overcomes (see (Bunin, 2017))

$$\sigma > \sigma_c = \sqrt{\frac{2}{S}}(\mu+1). \tag{39}$$

As S increases, this boundary decreases, and it becomes harder to find communities inside the unique fixed point regime (too many strong competitors lead to instability) (Bunin, 2017).

The EC regime, instead, is possible only if there are $A_{ij} < -1$ elements as described above. When S increases, the EC boundary does not lower as the multistability boundary does. Instead, it simply becomes sharper as S increases the size of the matrix and hence the likelihood of finding excluding elements, to the point where a small increase in σ *ensures* that $A_{ij} < -1$ elements will be found.

We show in figure B that the multistability line can only go above the EC boundary for very low $S \approx 10$, which is in fact a diversity where the approximations used for both lines are no longer accurate (Bunin, 2017). For larger communities, the typical expectation is that the multistability boundary is crossed before $A_{ij} < -1$ elements and EC have been observed: strong competition, even if not yet exclusionary competition, already leads to instability of the unique fixed point, fluctuations and multistability (Barbier, 2025). Related analyses of alternative stable states in GLV models appear in (Aguadé-Gorgorió and Kefi, 2024; Biroli *et al.*, 2018). Empirical EC experiments (Goldford *et al.*, 2018) also display multistability, where different initial conditions lead to distinct stable community compositions, consistent with this theoretical prediction.

Interestingly, this result is valid for uncorrelated matrices, meaning that A_{ij} and A_{ji} hold no correlation. In the extreme of strongly correlated (symmetric) matrices, we show in Section II.A.4

that EC is not possible and the EC boundary does not have meaning: even if excluding pairs exist in A, they never carry into A^* due to symmetry. In the extreme of strongly anticorrelated (antisymmetric, predator-prey) matrices, it has been shown that multistability is not possible and the multistability boundary vanishes (Bunin, 2017), so that EC happens for unique stable states.

3. Maximum competition allowed for emergent coexistence

The upper (competitive) bound of the EC regime is set by the onset of competitive exclusion, where interactions are sufficiently strong or homogeneous that only a single species persists (Aguadé-Gorgorió *et al.*, 2024). To identify this transition, we compute the probability that a stable two-species community can exist. This requires at least one pair (A_{ij}, A_{ji}) with both elements greater than -1, ensuring mutual non-exclusion.

Since A_{ij} and A_{ji} are independently sampled from $\mathcal{N}(\mu, \sigma)$,

$$P(A_{ij} > -1) = 1 - \Phi\left(\frac{-1-\mu}{\sigma}\right),$$

and therefore

$$P(A_{ij} > -1 \text{ and } A_{ji} > -1) = \left[1 - \Phi\left(\frac{-1-\mu}{\sigma}\right)\right]^2.$$

Let C denote this coexistence event. There are $S(S-1)/2$ unordered pairs. The probability that at least one pair satisfies C is

$$P(\text{at least one } C) = 1 - \left(1 - \left[1 - \Phi\left(\tfrac{-1-\mu}{\sigma}\right)\right]^2\right)^{S(S-1)/2}. \tag{40}$$

This probability again sharply transitions from 0 to 1 for large S, allowing us to estimate the critical line $\sigma_2^c(\mu)$ separating competitive exclusion from multistability in the GLV model numerically as done for σ_1^c. As in the minimal-competition calculation, we determine σ_2^c by solving $P(\text{at least one } C) = 0.5$ using the bisection method (SciPy `bisect`), yielding the bold red transition line in Fig. 2A. This line corresponds to the appearance of at least one potentially coexisting pair, complementing the dynamical mean-field estimates of the mono- to multi-stability boundary (Aguadé-Gorgorió and Kefi, 2024; Mallmin *et al.*, 2024).

A two-species coexistence event is necessary but not sufficient for EC, which also requires an exclusionary interaction. EC therefore requires either (i) a coexisting pair together with a third species that is excluded by one coexisting species and rescued by the other, or (ii) a three-species structure analogous to a Rock-Paper-Scissors (RPS) loop (Gilpin, 1975), where at least three elements of the corresponding A^* are greater than -1.

The probability of forming an RPS triplet cannot be computed analytically as easily as Eq. (40). Instead, we estimate it numerically by sampling random matrices with fixed μ (e.g., $\mu = -1.5$ in Fig. 2B), scanning over σ, and measuring the fraction of matrices containing: (i) at least one coexisting pair, and (ii) at least one triplet with all three required interactions > -1. Although not all such triplets lead to stable coexistence, the numerically observed probability of finding a candidate RPS triplet closely follows the analytical probability of finding a coexisting pair (Fig. C). This supports the use of Eq. (40) as a practical estimate of the upper EC boundary.

4. Emergent coexistence under alternative interaction structures

Figure D summarizes the occurrence of EC in GLV systems with alternative interaction matrices. Across all except one of the cases considered (antisymmetric, triangular, sparse, carrying-capacity-correlated, skewed Gamma distributions, and interaction matrices incorporating single-resource competition or cross-feeding) EC consistently emerges. This extends the results from the main text, where A_{ij} were Gaussian and uncorrelated, and highlights that EC is a consistent and quite generic outcome. In contrast, fully symmetric interactions ($A_{ij} = A_{ji}$) do not exhibit EC. This is consistent with the indirect-effects example in Section I.B.2: stable three-species loops require directional exclusion, i.e., if $A_{ij}, A_{jk}, A_{ki} < -1$, then the opposite elements must satisfy $A_{ji}, A_{kj}, A_{ik} > -1$. Asymmetry is therefore necessary to maintain a weak hierarchical structure (Koch *et al.*, 2024, 2023).

For symmetric interactions, we can prove mathematically that the absence of EC follows directly from stability constraints. The Jacobian at equilibrium is

$$J = \text{diag}(x_i^*)(-I + A^*),$$

and since $\text{diag}(x_i^*)$ is positive definite, the eigenvalues of J have negative real parts only if all eigenvalues of A^* are strictly below 1. Equivalently, $I - A^*$ must be positive definite. By Sylvester's criterion (Gilbert, 1991), this requires all leading principal minors of $(I - A^*)$ to be positive. The 2×2 minor already implies $A^*_{ij} = A^*_{ji} > -1$ for every coexisting pair, prohibiting strong competitive interactions. Thus symmetry enforces uniformly weak interactions and precludes the heterogeneous competitive structure required for EC.

Phase spaces for nested, sparse, carrying-capacity-structured ($\tilde{A}$) and antisymmetric matrices resemble the canonical GLV phase space (Fig. 2A of the main text; see Fig. D). Symmetric interactions produce a similar phase structure but lack EC, as expected from the analysis above and from (Bunin, 2017; Miller and Max, 2025). Gamma-distributed interactions generate a distinct phase space because the axes correspond to the shape α and scale θ parameters rather than (μ, σ) (Fig. DE). Here $\mu = \alpha\theta$ and $\sigma = \theta\sqrt{\alpha}$, consistent with (Hu *et al.*, 2022). Because the Gamma distribution is strictly positive, we apply a global sign change to ensure competition; this yields the symmetry at $\alpha = 0$.

For the single-resource model with cross-feeding, we vary the mean and standard deviation of C_{ij} while keeping competition terms fixed (Fig. DF). Unlike the main-text simulations, where $C_{ij} > 0$, here C_{ij} are Gaussian without sign constraints to isolate the effect of facilitation heterogeneity. Strongly negative mean C_{ij} produces excessive competition and suppresses species-rich EC states. EC reappears when C_{ij} have near-zero or positive mean and sufficient variability, allowing facilitation to offset single-resource competition. This supports the use of strictly positive cross-feeding in the main text. Because these systems still include an exclusionary single-resource component, the resulting multispecies states typically contain many exclusionary pairs (Fig. DF).

Overall, these results show that EC is robust across a wide range of interaction structures. EC requires heterogeneous and sufficiently competitive interactions, and so far we find that it only disappears under strong symmetry.

5. Emergent coexistence in other dynamical models with pairwise interactions

EC arises within the strong-interaction, multistable regime of the GLV model (Fig. 2A, main text), consistent with previous work identifying multiple stable species combinations driven by strong indirect effects (Aguadé-Gorgorió and Kefi, 2024; Altieri *et al.*, 2021; Biroli *et al.*, 2018; Bunin, 2017).

Multistability is not specific to GLV dynamics: similar "clique" regimes appear in systems with saturating cooperation, Allee effects, gene regulation, neural assemblies, or cancer-immune interactions (see (Aguadé-Gorgorió *et al.*, 2024) for the discovery of cliques across different dynamical models). In these models, strong heterogeneous interactions generate diverse indirect effects and multiple locally stable compositions. This suggests that EC should also appear broadly whenever pairwise interactions are strong enough to generate such indirect effects, independent of GLV linearity.

To test this expectation, we analyzed the models introduced in Section I.G, as well as a GLV model with external migration. Because simulations of high-dimensional systems with migration or nonlinear responses are computationally costly due to the fact that we have to test all pairwise tournaments numerically, we focused so far on whether stable states contain at least one excluding pair, following the approach of Fig. 2A.

Migration destabilizes many equilibria and produces long transients and switching among states (Aguadé-Gorgorió and Kefi, 2024; Gilpin, 2024; Mallmin *et al.*, 2024; Roy *et al.*, 2020). Nevertheless, the stable equilibria that remain frequently include excluding pairs (Fig. EA), at levels comparable to the standard GLV model. This indicates that migration does not disrupt the indirect positive effects responsible for EC.

Replacing linear interactions with saturating functional responses prevents runaway growth under strong cooperation and ensures coexistence at positive μ. With A_{ij} sampled as in the GLV model, the cooperation-dominated regime contains high-diversity equilibria in which exclusionary elements are almost surely present (Fig. EB). Once (μ, σ) exceed the threshold required for exclusionary interactions, EC is present regardless of saturation. Saturation limits dominance but does not suppress accumulation of indirect effects, allowing excluding pairs to persist in larger communities.

In the model separating competition (B) and cooperation (A) matrices (Aguadé-Gorgorió *et al.*, 2024), EC appears primarily within the multistability regime (Fig. EC). With relatively low heterogeneity ($\sigma_{A,B} \approx 0.1$), EC is less pervasive but follows the same pattern: strong, heterogeneous competitive effects are required. Outside this regime, excessive cooperation yields coexistence without exclusion, whereas excessive competition produces collapse to a single species.

For sublinear growth dynamics (Hatton *et al.*, 2024; Mazzarisi and Smerlak, 2024), the phase space differs from GLV but EC is very common (Fig. ED). This model stabilizes equilibria with increasing diversity; without extinction thresholds, no species go extinct. High diversity makes excluding pairs almost guaranteed. This parallels the high-cooperation regime of the saturating model. Introducing larger extinction thresholds would likely recover a GLV-like phase space (Aguadé-Gorgorió *et al.*, 2025).

Overall, across models (including migration, saturating responses, multilayer interactions, and

sublinear growth) EC reliably appears whenever interactions are strong and heterogeneous enough to generate indirect facilitation. These results suggest that EC is not a GLV-specific phenomenon but a generic outcome in species-rich systems governed by dense pairwise interactions.

Extended Figure C caption. *Purple shading indicates the fraction of stable states containing at least one excluding pair. EC is common across models and parameter regimes. Dashed lines denote regime boundaries lacking analytical expressions; red lines denote analytically defined boundaries. (A) GLV with migration: although migration reduces the number of stable states and increases persistent fluctuations, the remaining equilibria continue to exhibit EC. (B) GLV with saturating interactions: saturation removes the unbounded-growth regime and allows high-cooperation states in which EC is almost always present once* σ_1^c *is crossed. (C) Multilayer model: EC occurs primarily in the multistability regime, where heterogeneous competition supports coexistence of excluding pairs. (D) Sublinear growth model: EC is widespread due to large community sizes sustained at low abundances.*

6. Connectivity and its effects on coexistence and phase space structure

Throughout the main text we used fully connected interaction matrices ($C = 1$). This choice isolates the effects of the mean interaction strength μ and variability σ, simplifies analysis, and aligns with common GLV studies (Aguadé-Gorgorió and Kefi, 2024; Bunin, 2017; Mallmin *et al.*, 2024). Given that many empirical systems have $C < 1$, we show here that EC appears in the GLV model across both high and low connectivity values. Here we summarize preliminary results for $C < 1$.

Figure 2C in the main text shows that the GLV model with $C = 1$ typically yields equilibria with at most tens of surviving species (Aguadé-Gorgorió and Kefi, 2024), far below the richness of many natural communities. This agrees with the classical diversity-stability relationship: increasing diversity generally destabilizes equilibria. The diversity levels in Fig. 2C match many synthetic laboratory communities, which tend to be more densely connected (fewer niche dimensions, shared limiting resources, fewer survivors) than natural ecosystems (Chang *et al.*, 2023; Friedman *et al.*, 2017; Venturelli *et al.*, 2018). Our goal here is not to revisit the diversity-stability paradox; see (Bunin, 2017; Grilli *et al.*, 2017a; Marcus *et al.*, 2024; Serván *et al.*, 2018). Instead, we assess how changing C affects coexistence and EC.

Relaxing full connectivity produces a consistent trend: reducing C increases the number of coexisting species. Figure F illustrates how stable diversity grows as C decreases. However, the shape of the phase space also changes. For $C \simeq 1$ (Fig. 2A and Fig. FA), coexistence is common at low σ due to weak, nearly absent competition; increasing σ then drives extinctions. At intermediate C, effective heterogeneity increases and the effect on coexistence becomes non-monotonic. At very low C, many more species survive (Fig. FC).

These patterns follow from the properties of the sparsified matrix A_C:

$$A_{C,ij} = \begin{cases} 0 & \text{with probability } 1 - C, \\ A_{ij} & \text{with probability } C, \end{cases} \qquad A_{ij} \sim \mathcal{N}(\mu, \sigma). \tag{41}$$

The mean interaction becomes

$$\mathbb{E}[A_{C,ij}] = C\mu,$$

and the standard deviation is (Aguadé-Gorgorió and Kefi, 2024)

$$\mathrm{std}(A_{C,ij}) = \sqrt{C\sigma^2 + C(1-C)\mu^2}. \tag{42}$$

Slightly reducing C increases heterogeneity because strong interactions coexist with many zeros, which can reduce coexistence. Large reductions in C suppress heterogeneity because most interactions vanish, favoring coexistence. At very low C, coexistence becomes relatively insensitive to σ: increasing σ adds potential strong competitors, but most are "zeroed out" before affecting dynamics.

Despite these changes, the GLV model retains a broad parameter region where EC is frequent. Lowering connectivity generally expands the coexistence regime (Fig. F), but does not alter the main conclusion: EC emerges wherever strong, heterogeneous interactions generate positive indirect effects, regardless of C.

Again, we do not argue that sparse connectivity alone explains high diversity in natural ecosystems; this has been the focus of extensive theoretical and empirical research (Calleja-Solanas *et al.*, 2022; Chesson, 2000; Grilli *et al.*, 2017b; Hatton *et al.*, 2024; Ives and Carpenter, 2007; Jacquet *et al.*, 2016; May, 2019; Mazzarisi and Smerlak, 2024; McCann, 2000; Tilman *et al.*, 1998). Nonetheless, recent studies suggest that lower connectivity may help explain empirical patterns in both microbial and macroorganism communities (Arya *et al.*, 2023; Camacho-Mateu *et al.*, 2024; Marcus *et al.*, 2022). In this context, laboratory microbial communities may show lower diversity because they are more densely connected (few shared resources), whereas natural ecosystems contain many functional groups and therefore fewer direct interactions.

At first sight, the role of connectivity is secondary for EC: changing C alters coexistence levels and phase space structure but does not modify the basic mechanism by which strong, heterogeneous interactions generate emergent coexistence. We study how $C < 1$ modulates the fraction of excluding pairs in the next section, and leave for future work more in-depth quantitative analysis of how connectivity modulates EC through reducing the length of indirect effects.

B. The fraction of excluding pairs (Figure 2C)

Following (Chang *et al.*, 2023), we quantified not only whether a state contains excluding pairs but also the *fraction of excluding pairs.* For a community with S^* surviving species, there are $S^*(S^*-1)/2$ species pairs. We counted how many of these pairs are excluding, defined as cases where $A^*_{ij} < -1$, $A^*_{ji} < -1$, or both.

In Fig. 2C (main text), we study the communities with EC found in Fig. 2A. For each stable community exhibiting at least one excluding pair, we computed the fraction of excluding pairs; communities without EC have a value of zero and were omitted from the plot. For example, for $S^* = 3$, possible structures include a Rock–Paper–Scissors cycle (3 excluding pairs), two excluding pairs, or a single excluding pair, corresponding to fractions of 1, 0.66, and 0.33, respectively (see Section I.B.2). The minimal possible fraction is therefore 1 over all $S^*(S^*-1)/2$ species pairs,

so $2/[S^*(S^*-1)]$, shown as a dashed line in Fig. 2C. Here we describe the experimental datasets studied, the mathematical methods to estimate the maximum fraction of excluding pairs, and the fraction of excluding pairs in different matrix randomizations.

1. Gathering data on the fraction of excluding pairs

We aimed to quantify empirical signatures of EC, focusing on the fraction of species pairs that coexist in a stable community but exclude one another in pairwise co-culture. We compared these empirical values with our theoretical upper bound on the number of excluding pairs as a function of diversity (Fig. 2C, main text). To ensure reliable pairwise data, we only used studies in which co-culture outcomes were directly measured, excluding datasets that infer interactions solely from time series but do not test the pairwise interaction in isolation. We also required that communities be assembled and observed in the laboratory under stable or stationary conditions.

Below we summarize the four datasets used in Fig. 2C, as well as additional datasets examined but not plotted because they displayed no excluding pairs.

Dataset 1: Soil- and Plant-Derived Communities ((Chang *et al.*, 2023)). Chang *et al.* assembled communities in glucose-limited M9 minimal medium using microbiomes from 12 soil and plant sources (Goldford *et al.*, 2018). Initial species pools contained 110–1290 exact sequence variants (ESVs). After 12 sequential transfers (∼84 generations), communities stabilized at fewer than 25 coexisting ESVs, dominated by Enterobacteriaceae and Pseudomonadaceae. Stationarity was assessed through invasion fitness measurements: ESVs present at the final passage were able to reinvade from low frequency. From these 12 communities, 62 isolates were obtained, and 144 pairwise co-cultures produced conclusive outcomes. Because not all species could be isolated, the effective diversity analyzed in Fig. 3A of (Chang *et al.*, 2023) ranges from 3 to 10 species. We extracted the number of excluding pairs for each community from their tournament matrices and computed the empirical fraction of excluding pairs as

$$\frac{\text{number of excluding pairs}}{S^*(S^*-1)/2}.$$

Pairs classified as “competitive exclusion” were counted as excluding. Pairs categorized as “on the path to exclusion” were excluded from our counts to avoid ambiguity regarding true extinction versus strong competitive asymmetry without extinction. Including those pairs would push several communities above our theoretical upper bound (red dashed lines in Fig. 2C of the main text), which is expected as the limit is only a statistical bound. Among the 12 communities, 66% contained at least one clear excluding pair, and 100% contained either an excluding pair or at least one interaction on the “path to exclusion”. From Fig. 3A of (Chang *et al.*, 2023), we extracted fractions for communities with $S^* \in \{3, 4, 5, 7, 9, 10\}$. Repeated values (identical S^* and identical excluding-pair counts) were retained without special marking. Chang *et al.* also report absence or near-absence of rock–paper–scissors triplets and low-rank exclusion motifs, indicating predominantly transitive competition.

Dataset 2: Synthetic Soil-Derived Communities ((Friedman *et al.*, 2017)). Friedman *et al.* studied 8 heterotrophic soil bacterial species grown in M9 minimal medium for five growth–dilution cycles (∼53 generations). They measured outcomes for all 28 pairs, all 56 three-species combinations, the full 8-species pool, and all 7-species initial combinations. From their Fig. 2d,

we reconstructed the pairwise tournament matrix. Using this matrix, we assessed EC in three-species communities (Fig. 3 of their main text) and in four-species communities observed within the larger assemblies (Fig. 5a). Only 8.7% of the three-species assemblages exhibited EC. Among the communities assembled from 7 or 8 initial species, two communities exhibited EC and one did not, yielding a 66% EC incidence across these few observations. From these data, we extracted the number of excluding pairs for communities with $S^* = 3$ and $S^* = 4$ surviving species. As before, repeated values are retained without special marking.

Dataset 3: Human Gut Bacterial Community ((Venturelli *et al.*, 2018)). Venturelli *et al.* assembled a 12-species synthetic community and all 11-species (one dropped) communities using intestinal bacterial strains. All 12 monocultures and all 66 pairwise co-cultures were performed, enabling direct estimation of growth parameters and GLV interaction coefficients. Pairwise outcomes again showed frequent competitive exclusion (approximately 50% of pairs). After parameterizing a GLV model, the authors assembled one 12-species community and 12 communities starting with 11 species. Using their pairwise outcomes (Fig. 2A) and community time series (Dataset EV3), we estimated which species persisted to the final time point. Species with relative abundances $\leq 10^{-4}$ were treated as extinct (consistent with the survival threshold used in our simulations). For each community, we computed the fraction of excluding pairs among the S^* surviving species. The resulting values correspond to communities with $S^* \in \{6, 7, 8, 9, 10\}$ (no community retained all 11 or 12 species). This dataset yielded noticeably higher fractions of excluding pairs than the other empirical datasets. We do not propose a specific mechanism for this difference; it may reflect system-specific interaction structure, resource conditions, or initial abundance effects. We hypothesize that it could be related to a skewed distribution of interaction strengths constistent with (Koch *et al.*, 2023). Interestingly, the data provided in (Venturelli *et al.*, 2018) also contains inferred mesurements of A^* between all constituent species in these different stable communities, which we later use to measure ϕ and κ values for these empirical matrices in Fig. 3 of the main text.

Classical Rock–Paper–Scissors Community ((Kerr *et al.*, 2002)). For reference, Fig. 2C includes a rock–paper–scissors (RPS) example in *E. coli* (Kerr *et al.*, 2002). The community consists of three strains: a colicin-producing strain (C), a sensitive strain (S), and a resistant strain (R). The producer (C) kills the sensitive strain (S) via colicin; the sensitive strain (S) grows faster than the resistant strain (R) because it avoids the cost of resistance; and the resistant strain (R) outcompetes the producer (C) by avoiding the metabolic cost of toxin production. This cyclic dominance stabilizes coexistence of all three strains. In the GLV framework, these strains correspond to three distinct "species" with different interaction strengths, giving $S^* = 3$. Because each pairwise interaction contains exactly one excluding direction, the exclusion fraction for this community is 1.

Additional Datasets Without Excluding Pairs. Several studies confronting pairwise and multispecies outcomes reported no excluding pairs, and therefore do not contribute information to Fig. 2C. For example: (Lele *et al.*, 2024) identified 3 excluding pairs among 36 pairwise tests in sourdough-derived species, but none of these pairs coexisted in multispecies assemblies (diversity ~ 6). (Castledine *et al.*, 2024) assembled a 5-species soil microbial community in which every species invaded every subcombination, indicating full coexistence. Similar results were found in protist communities of varying sizes (Pennekamp *et al.*, 2018). Some communities in (Friedman *et al.*, 2017) also displayed no excluding pairs. As these cases provide zero-valued fractions, we excluded them from Fig. 2C for clarity.

2. Maximum fraction of excluding pairs a community can sustain

Figure 2C summarizes the fraction of exclusionary pairs observed in stable communities of different diversities across GLV simulations with interaction matrices $A \sim \mathcal{N}(\mu, \sigma)$. Here we outline the analytical procedure used to estimate the maximum fraction of exclusionary pairs that a stable community of size S^* can sustain.

a. Step 1: Estimating the largest feasible community size S^*. For a given (μ, σ) inside the multistability domain (Fig. 2A), we estimate the largest possible stable community that we can find following (Aguadé-Gorgorió and Kefi, 2024). The least competitive stable subset given an original pool with (μ, σ) was found to satisfy:

$$\max(\mu^*) \approx \mu + \frac{4\sigma}{\sqrt{S^*(S^*-1)}}, \tag{43}$$

This implies that subsets can be less competitive than the original pool, yet the statistics end up ressembling those of the original pool as S^* increases.

The most competitive stable subset has a lower bound dictated by the dynamics (Bunin, 2017; Mallmin *et al.*, 2024):

$$\min(\mu^*) \approx \sqrt{\frac{S^*}{2}}\,\sigma^* - 1, \tag{44}$$

with the finite-size numerical correction (Aguadé-Gorgorió and Kefi, 2024):

$$\min(\mu^*) \approx \frac{(S^*)^{1.08}}{14.118}\,\sigma^* - 1. \tag{45}$$

The term σ^* depends on the subset size. The minimum possible standard deviation of a subset of $S^*(S^*-1)$ elements drawn from a Gaussian is (Aguadé-Gorgorió and Kefi, 2024):

$$\min(\sigma^*) = 1 - \sqrt{1 - \frac{2}{S^*(S^*-1)-1}\left(\frac{\Gamma_1}{\Gamma_2}\right)^2}, \tag{46}$$

where

$$\Gamma_1 = \Gamma\left(\frac{S^*(S^*-1)}{2}\right), \qquad \Gamma_2 = \Gamma\left(\frac{S^*(S^*-1)-1}{2}\right).$$

Combining Eqs. (43)-(45), we determine S^* by solving $\max(\mu^*) = \min(\mu^*)$ for $S^* \in [2, 50]$. We use a brute-force search to find the S^* that minimizes the mismatch $\epsilon = \max(\mu^*) - \min(\mu^*)$. This gives the predicted maximum diversity attainable for each (μ, σ) that we sample across the parameter space of Fig. 2A in the main text.

b. Step 2: Fraction of exclusionary pairs in the pool. We next estimate the maximum fraction of exclusionary pairs that any stable state can inherit from the pool. Because coexistence disfavors strong competition, stable communities very rarely contain more exclusionary interactions than

the pool A. We refer the reader to (Aguadé-Gorgorió and Kefi, 2024) for the detailed analysis. The starting assumption is, therefore, that the maximum fraction of excluding pairs in A^* given (μ, σ) is equivalent to the maximum fraction of excluding pairs in A.

For a pair (i, j), at least one of A_{ij} or A_{ji} is exclusionary if it is < -1. Since entries are independent,

$$P(A_{ij} < -1 \text{ or } A_{ji} < -1) = 1 - P(A_{ij} \geq -1)P(A_{ji} \geq -1),$$

where

$$P(A_{ij} \geq -1) = 1 - \Phi\left(\frac{-1-\mu}{\sigma}\right).$$

Thus the probability that one pair is an excluding pair is

$$f(\mu, \sigma) = 1 - \left(1 - \Phi\left(\frac{-1-\mu}{\sigma}\right)\right)^2. \tag{47}$$

The probability that a pair is an excluding pair is equivalent to the fraction of excluding pairs in a subset.

c. Combining both steps. For each sampled (μ, σ), Step 1 yields a statistical bound for the maximum predicted diversity S^*, and Eq. (47) provides the corresponding fraction of exclusionary pairs in the pool. Because we are sampling many (μ, σ) values, we need to find the largest fraction found across those values. Recording all (S^*, f) pairs across many (μ, σ) samples and keeping the largest f associated with each S^* yields a numerical upper envelope for the fraction of exclusionary pairs that a community of size S^* can sustain. This curve appears as the red dashed line in Fig. 2C.

3. Fraction of pairs in sparse and hierarchical matrices

In Fig. GA-C we show and describe how the fraction of excluding pairs found in stable communities with EC changes as connectivity decreases. We repeat the analysis of Fig. 2C from the main text but vary the connectivity C. As expected from the results shown in Figure S5 (where lower connectivity leads to higher equilibrium richness) we find that reducing C makes small communities with EC increasingly rare, while communities with more EC appear more frequently thanks to C reducing overall competition and allowing higher diversity. Nevertheless, all observed community sizes remain consistent with the analytical boundaries predicted in the main text.

Figure GD-F shows the fraction of excluding pairs in the EC communities identified in hierarchical matrices T, B and $\tilde{A}$, comparable to Fig. 2A in the main text. The qualitative dependence of exclusion fraction on diversity is similar to that observed in the random GLV model. A more detailed quantitative comparison across matrix classes is left for future work.

C. Interaction patterns in A^* and coexistence

After identifying EC as a common outcome of the GLV model without higher-order interactions, we examined which microscopic interaction patterns might underlie it. We show that intransitivity

is not required: intransitive loops may occur but EC also arises in triangular or loop-free structures. This motivated a systematic search for other small-scale interaction signatures, similar to the patterns drawn in Fig. 1B of the main text.

Work such as (Barbier *et al.*, 2021) provides relevant context. Given a random species pool, they asked whether coexisting species exhibit non-random interaction signatures. Two patterns appeared in both models and plant community data: (i) strongly abundant competitors avoid directly competing with one another, and (ii) they target distinct species. These patterns imply that competitive pressure is diffuse rather than concentrated. Similar features occur in GLV cliques, the alternative stable states associated with EC (Aguadé-Gorgorió and Kefi, 2024), and related ideas appear in (Poley *et al.*, 2025).

To test whether specific microscopic motifs explain EC, we sampled random species subsets, constructed their interaction matrices A^*, and computed a large set of statistics. We classified subsets as unfeasible (row sums of $(I - A^*)^{-1}$ not positive) or feasible, and among feasible cases distinguished linearly unstable from linearly stable communities. We asked whether any metric clearly differentiated matrix motifs in these categories.

Because EC requires at least feasibility (positive abundances), our main focus was identifying signatures associated with feasibility rather than stability. Contrary to the intuition that strong negative interactions $A_{ij} < -1$ might require compensatory indirect effects $A_{ij}A_{jk} > 0$, feasible and unfeasible subsets differed only weakly across many metrics. Visual inspection of violin plots likewise revealed no distinct EC-specific signatures beyond the diffuse coexistence fingerprints already reported in (Aguadé-Gorgorió and Kefi, 2024; Barbier *et al.*, 2021). Metrics that showed only mild differences (not shown) include: (i) effective means and variances (μ^*, σ^*) of A^*; (ii) fraction of positive net effects; (iii) skewness and kurtosis, with feasible states slightly more negatively kurtotic; (iv) heterogeneity of column sums in A^*; (v) collectivity and condition number; and (vi) short-chain motifs ($A^*_{ij} < -1$ with $A^*_{ij}A^*_{jk} > 0$) and correlations between A^* and $(A^*)^2$.

Metrics of type (vi) indicated the absence of systematic short rescue chains. Although such motifs can produce coexistence in three-species systems (Section I.B), they do not generalize to communities with four or more species. This suggested that short-chain analysis misses the influence of long interaction chains. High collectivity amplifies these long chains, making short or second-order motifs poor predictors of feasibility. When the spectral radius is large, abundances are not well explained by A^* or $(A^*)^2$, which become uncorrelated with the row sums of $(I-A^*)^{-1}$. Classic motifs such as "enemy of my enemy" or rock-paper-scissors effectively vanish.

Beyond the diffuse patterns described in (Barbier *et al.*, 2021) and the overall lack of few-species motifs, one clear signature did emerge. The row sums of A^*, $\sum_j A^*_{ij}$, were significantly more homogeneous than expected under randomization, unlike row sums in unfeasible subsets or column sums in feasible ones (Fig. H). We quantified this by comparing the standard deviation of row sums to the average standard deviation from 100 randomized versions of A^*. Ratios below 1 indicate increased homogeneity. Feasible EC communities consistently showed such reductions, implying that coexisting species experience similar total competitive pressure. In contrast, column sums $\sum_i A^*_{ij}$, representing a species' impact on others, showed no such homogenization (not shown). Thus, coexistence requires species to perceive comparable competition, but does not constrain how strongly they affect others.

This pattern follows directly from feasibility conditions. If all abundances were equal, $x_i =$

$1+\sum_j A_{ij}x_j$ would imply that $\sum_j A_{ij}$ is the same for all species, so identical abundances require identical row sums. Actual equilibrium abundances are not necessarily equal, but feasibility still requires that they are all positive. This enforces a partial concentration of row sums, which are less random than if x_i^* values could be either positive or negative.

D. All-to-all competition but positive net effects

To complement the analysis of A^*, we examined properties of the net-effect matrix $(I - A^*)^{-1}$. Using the Neumann series,

$$(I - A)^{-1} = I + A + A^2 + A^3 + \dots, \tag{48}$$

the equilibrium abundance of species i can be written as

$$x_i^* = 1 + \sum_j A_{ij}^* + \sum_{j,k} A_{ik}^* A_{kj}^* + \sum_{j,k,l} A_{ik}^* A_{kl}^* A_{lj}^* + \dots. \tag{49}$$

Thus, even when all direct interactions are competitive, indirect effects can generate positive net effects.

We quantified this using the "Positive Feedback Index" of (Liautaud *et al.*, 2019), defined as the fraction of positive entries in $(I-A^*)^{-1}$. Because these entries need not correspond to closed loops, we simply refer to them as the fraction of positive net effects without refering to *feedback*. Under the symmetric-interaction assumptions in (Liautaud *et al.*, 2019), this fraction reaches at most $\sim 15\%$. In contrast, in our GLV model with random, asymmetric interactions, once interaction strengths are sufficiently large and communities enter the EC regime, the fraction of positive net effects approaches 50% (Fig. I), which we hypothesize is related to the lack of correlation between direct and net effects discussed below. Even at weaker competition, coexistence states already contain a substantial proportion of positive net effects before EC appears.

This behavior is also evident in Fig. 3C of the main text: although most A_{ij}^* are negative, roughly half of the corresponding net effects $(I-A^*)_{ij}^{-1}$ are positive. The discrepancy with (Liautaud *et al.*, 2019) may stem from their assumptions of symmetry and absence of extinctions, both of which restrict the range of stable equilibria with strong interactions as discussed above.

The emergence of $\sim 50\%$ positive net effects links EC to the dominance of long interaction chains when interactions are strong. For weak interactions, higher powers of A vanish quickly, making abundances largely determined by self-regulation or direct interactions so that most net effects are negative as species compete. As interaction strengths increase, long chains contribute substantially and often outweigh direct competition, producing many positive net effects. These indirect effects enable coexistence of species pairs that would not survive in isolation, providing a mechanistic basis for EC.

E. Collectivity and net effects (Figure 3A-C)

This section describes the numerical procedures and analytical developments used to compute the collectivity parameter ϕ from stable communities with EC.

1. Computing the Spectral Radius ϕ

For each stable community, we obtain its interaction matrix A^* (with zero diagonal entries; self-regulation is represented separately as an identity matrix, Section I.B.3). Following (Zelnik *et al.*, 2024), the collectivity parameter is defined as the spectral radius of A^*, i.e.,

$$\phi = \rho(A^*) = \max_i |\lambda_i|,$$

where λ_i are the eigenvalues of A^*. This quantity measures the amplification of indirect effects through the network and the convergence of the Neumann series. We compute ϕ numerically using `numpy` via:

```
phi = max(abs(numpy.linalg.eigvals(A_star)))
```

2. Data plotted in Figures 3A,D

To construct Fig. 3, we compute ϕ and κ for stable communities with and without EC. Communities with EC (purple points) are obtained by simulating the GLV model with (μ, σ) sampled from the domain of Fig. 2A and selecting final stable states that contain excluding pairs, as in Fig. 2C.

To obtain stable communities *without* EC across a broad range of sizes, direct GLV integration is insufficient: with all species initially present, weak interactions place the system in a unique fixed-point regime where most species survive (Aguadé-Gorgorió and Kefi, 2024; Bunin, 2017). To access smaller communities without EC and with lower ϕ or κ, we randomly sample subsets of species and evaluate feasibility and stability directly from the sampled submatrices of A. This method is computationally efficient and is used for the non-EC points in Figs. 3 and 4, as well as in the subsequent stability analyses.

We also measure ϕ and κ for the empirically inferred interaction matrices A^* published in (Venturelli *et al.*, 2018) (see the description of the dataset above). Other datasets containing observations of EC did not infer the underlying interaction matrices A^*.

3. Estimates for minimal and maximal collectivity

To complement the numerical results in Fig. 3A (main text), we provide analytical estimates for how collectivity grows with diversity and interaction strength. Collectivity quantifies the extent to which indirect interaction chains contribute to net effects and therefore indicates when direct interactions become insufficient to explain equilibrium structure.

Following (Allesina and Tang, 2012) and the derivations summarized in (Zelnik *et al.*, 2024), the spectral radius of a random interaction matrix with mean μ^*, standard deviation σ^*, connectivity C, reciprocity γ^*, and S^* species is approximated by

$$\phi_{\text{rmt}} = \max\left\{(S^* - 1)C|\mu^*|,\ (1 + |\gamma^*|)\sigma^* \sqrt{C(S^* - 1)}\right\}, \tag{50}$$

with $C = 1$ in the main text. Although originally derived for Jacobians, these results also approximate the spectral radius of A^* and thus the expected magnitude of indirect effects.

Minimal collectivity in the GLV model is zero when $\mu^*, \sigma^* \approx 0$, but such cases cannot generate EC because they lack exclusionary interactions. To characterize collectivity specifically for EC states, we use the known constraints on feasible (μ^*, σ^*) for a given S^* used in Step 1 of the calculation of the maximum fraction of excluding pairs and in (Aguadé-Gorgorió and Kefi, 2024). Larger species sets admit weaker mean competition, so, even in S^* increases, $|\mu^*|$ decreases with increasing S^*. Consequently, even the maximal collectivity is not exactly linear in S^*: increasing diversity increases the number and length of indirect chains but reduces the magnitude of competitive interactions. Numerically, the latter effect is small, and we find that the upper envelope of collectivity is well approximated by

$$\max(\phi_{\mathrm{EC}}) \approx (S^* - 1)\max(|\mu^*|) \approx (S^* - 1)\left(1 - \frac{(S^*)^{1.08}}{14.118}\min(\sigma^*)\right), \tag{51}$$

where $\min(\sigma^*)$ is the smallest feasible standard deviation for an S^*-species community arising from the original pool (Aguadé-Gorgorió and Kefi, 2024). This estimate holds for moderate diversity; for small systems ($S^* \leq 4$), collectivity can exceed this bound because finite-size effects modify the feasible region. Near the neutral point $\mu = -1$ (Kessler and Shnerb, 2015), EC occurs as $\sigma^* \to 0$ (main text Fig. 2A), implying ϕ can grow linearly with S^*.

We also estimate the minimal collectivity compatible with EC. For small S^*, μ^* can be near zero even if EC does not occur at $\mu = 0$ for the full species pool. For larger communities, the statistics of A^* converge to those of A (Aguadé-Gorgorió and Kefi, 2024), so the minimal $|\mu|$ supporting EC determines the smallest possible collectivity. From numerical estimation using the phase space of figure 2A, we find that the weakest competition consistent with EC is $\min(|\mu|) \approx 0.071$, meaning that some communities can harbor slightly positive mean interactions as well as excluding pairs due to heterogeneity. This gives

$$\min(\phi_{\mathrm{EC}}) \approx (S^* - 1) \times 0.071. \tag{52}$$

Because the unbounded-growth boundary (main text Fig. 2A, vertical dashed line) lacks a closed analytical form to our knowledge, the value 0.071 is based on numerical inspection. It nevertheless provides a reliable lower bound on collectivity for EC states (main text Fig. 3A, lower red dashed line).

Finally, we show that collectivity provides key intuitions regarding the correlation between direct and net effects. As shown in Fig. 3B,C (main text) and Fig. J, communities with weak interactions exhibit strong correlation between A^* and $(I - A^*)^{-1}$ because indirect effects are small. In contrast, EC states, because they necessarily hold strong interactions, show weak or no correlation, and in very small species sets even anticorrelation: species that directly compete may indirectly facilitate each other through the rest of the community, a clear signature of RPS motifs. As S^* decreases, the dominance of long indirect chains drives A^* and $(I - A^*)^{-1}$ toward zero correlation, underscoring that direct effects alone become uninformative when collectivity is high.

4. Collectivity and irreducible interaction chains

Indirect effects are often illustrated through simple three-species chains (Section I.B.2), where species 3 affects species 2, species 2 affects species 1, and thus species 3 indirectly affects species 1, corresponding to the product $A_{12}A_{23}$. While such motifs motivate the idea of collectivity, the spectral radius ϕ measures a broader property of the interaction matrix and is not restricted to such irreducible three-species chains. As EC requires at least three species, our analysis focuses on $S^* \geq 3$, but ϕ is defined for all community sizes.

This broader meaning is clearer when examining the Neumann series for a two-species system. For example,

$$A^2 = \begin{pmatrix} A_{11}A_{11} + A_{12}A_{21} & A_{11}A_{12} + A_{12}A_{22} \\ A_{21}A_{11} + A_{22}A_{21} & A_{21}A_{12} + A_{22}A_{22} \end{pmatrix}, \tag{53}$$

showing that second-order indirect effects can arise even without three-species paths. Terms such as $A_{21}A_{11}$ and $A_{22}A_{21}$ reflect self-mediated feedbacks, demonstrating that collectivity captures general amplification via matrix powers rather than only chain motifs of the form $A_{12}A_{23}$.

Related to this, $\phi > 1$ is not a strict requirement for EC. As observed in Fig. 3A (main text), EC can occur with $\phi < 1$ in small communities. The spectral radius reflects the magnitude of a single eigenvalue, whereas the cumulative influence of many indirect pathways may be substantial even when ϕ is modest. Consequently, communities with low ϕ may still contain a few exclusive pairs (Fig. J, purple points). However, the likelihood that strong direct–net correlations coexist with EC decreases as S^* increases. In larger communities, strong interactions and EC typically produce substantial indirect effects, weaker direct–net correlations, and correspondingly larger ϕ (main text Fig. 3A,C).

Overall, we use ϕ as a practical proxy for the weight of indirect effects in sufficiently large random communities. More detailed characterization of indirect effects in small and, more importantly, structured non-random matrices likely requires alternative metrics beyond the spectral radius.

F. Condition number (Figure 3D,E)

This section describes the numerical procedures and analytical developments used to compute the condition number κ from stable communities.

1. Computing the Condition Number κ

The condition number of $(I - A^*)$ quantifies the sensitivity of matrix inversion and determines error propagation. For a detailed mathematical discussion, see (Demmel, 1987; El Ghaoui, 2002; Gilpin, 2024). We compute

$$\kappa = \kappa(I - A^*)$$

using both `numpy.linalg.cond` directly, or by taking the ratio of the largest to smallest singular values obtained from `numpy.linalg.svd`.

2. Estimating the minimal condition number κ

When direct interactions do not reliably predict net effects, one must compute $(I - A^*)^{-1}$ to obtain equilibrium abundances. Numerical accuracy of this inversion is governed by the condition number κ, a standard measure of matrix invertibility and error amplification (Edelman, 1988; Trefethen and Bau, 2022). For the GLV equilibrium

$$(I - A)\boldsymbol{x} = 1, \qquad \boldsymbol{x}^* = (I - A^*)^{-1}1, \tag{54}$$

$\kappa(I - A^*)$ quantifies how small perturbations in A^* affect $(I - A^*)^{-1}$. A value near 1 indicates numerical stability; large values imply that small errors in A^* lead to large errors in predicted abundances.

For a general matrix, the (2-norm) condition number is

$$\kappa(I - A^*) = \frac{s_{\max}}{s_{\min}}, \tag{55}$$

where $s_{\max}$ and $s_{\min}$ are the largest and smallest singular values. For symmetric positive definite matrices these correspond to eigenvalues, but EC primarily occurs in asymmetric, uncorrelated interaction matrices.

We use known results on singular values of random matrices to estimate how κ scales with S^*, μ^*, and σ^*. For an i.i.d. Gaussian matrix with standard deviation σ^*,

$$s_{\max} \approx 2\sigma^* \sqrt{S^*}, \tag{56}$$

$$s_{\min} \approx \frac{\sigma^*}{\sqrt{S^*}}, \tag{57}$$

for large S^* (Edelman, 1988). Although $(I - A^*)$ is not fully random (its diagonal is fixed), these expressions provide a useful approximation.

Our goal is not an exact formula for κ, but an analytical bound for EC states. The largest singular values occur for EC states with maximal heterogeneity and weakest competition (largest μ^*, largest σ^*), whereas the smallest $s_{\max}$ values occur for the strongest competition (smallest μ^*) and low heterogeneity. Across EC states,

$$s_{\max} \in \left(|\min(\mu^*)| + 2\, \min(\sigma^*)\sqrt{S^*},\ |\max(\mu^*)| + 2\, \max(\sigma^*)\sqrt{S^*} \right), \tag{58}$$

with μ^* and σ^* estimated either analytically or recorded during simulation.

Estimating κ requires an upper estimate of $s_{\min}$. Small singular values are highly variable (Fig. KB,C), and thus many EC states exhibit extremely large κ. We restrict attention to the maximal possible value of $s_{\min}$ within EC states, i.e. the best-case scenario for predictability. Random matrix theory suggests

$$s_{\min} \in \left(0,\ \frac{\max(\sigma^*)}{\sqrt{S^*}} \right), \tag{59}$$

with the upper bound reached for states with high σ^*.

Combining these gives a lower bound for the condition number among EC states:

$$\min \kappa(I - A^*) \approx \frac{|\min(\mu^*)| + 2\, \min(\sigma^*)\sqrt{S^*}}{\max(\sigma^*)/\sqrt{S^*}}. \tag{60}$$

For small communities with nearly homogeneous interactions (very small σ^*),

$$\min \kappa(I - A^*) \approx \frac{|\min(\mu^*)|}{\max(\sigma^*)}\sqrt{S^*}, \tag{61}$$

whereas larger communities, which cannot maintain such homogeneity, recover the scaling

$$\min \kappa(I - A^*) \sim S^*. \tag{62}$$

Thus, even in the most favorable (low κ) EC states, the condition number increases at least linearly with diversity. Most observed EC states exceed this theoretical minimum (Fig. KD, purple points), whereas only weakly interacting, homogeneous systems approach $\kappa \approx 1$ (gray points).

(i) In some cases, direct numerical integration of the GLV dynamics (Section I.E.1) may propagate interaction-measurement errors less severely than matrix inversion (Sections I.E.2 and I.I). A systematic comparison lies beyond our scope; see (Gilpin, 2024) for recent work linking temporal unpredictability and condition numbers.

(ii) High κ suggests that EC communities may have low structural stability (Cenci and Saavedra, 2018; Rohr *et al.*, 2014; Saavedra *et al.*, 2017), meaning that small changes in A^* can strongly impact predicted abundances and potentially species persistence. Exploring how structural stability volumes change in EC communities, and whether they exceed those predicted from pairwise interactions, offers a promising direction for future work.

G. Applicability of ϕ and κ in Empirical Tests

We evaluate how two quantities—the collectivity parameter ϕ and the condition number κ—govern the reliability of predicting community coexistence from pairwise interaction data. These numerical tests parallel experimental workflows used in microbial community assembly (Arya *et al.*, 2025; Solé *et al.*, 2024). We consider two scenarios:

1. (Test 1) Upscaling pairwise coexistence to multispecies coexistence and its dependence on ϕ.
2. (Test 2) Predicting coexistence from noisy interaction measurements and the role of κ.

All tests use species drawn from a pool of size $S = 100$, with interaction matrices sampled from the domain of Fig. 2A. Interactions with $A_{ij} < -1$ are treated as excluding; all others permit coexistence. Community coexistence is defined by positivity of all row sums of $(I - A^*)^{-1}$.

1. Test 1: Assembly from Pairwise-Coexisting Species

We construct random subsets of size $S^* \in [2, 15]$ such that all pairwise interactions within the subset permit coexistence. For each subset:

1. We evaluate whether the full community coexists by checking positivity of the row sums of $(I - A^*)^{-1}$.

2. We compute the collectivity parameter ϕ of the corresponding A^*.

We repeat this procedure to obtain 10^5 samples. To estimate the probability of community coexistence as a function of ϕ, we apply a rolling-mean smoothing procedure: samples are sorted by ϕ, and sliding-window means (window size: 100; step: 1) yield smoothed coexistence probabilities. The central observation is that, as ϕ increases, the probability that assembling a coexisting community built on pairwise coexistors decreases. This establishes a mathematical foundation for the previous observations of (Friedman *et al.*, 2017).

Assembly success is strongly controlled by ϕ (Fig. LA). For $\phi \to 0$, indirect effects are weak and nearly all communities assembled from coexisting pairs also coexist. As ϕ increases, success declines: around $\phi \approx 1$, approximately half of communities fail, and for $\phi \gtrsim 3$ most assembled communities do not coexist, even if all pairs did.

These failures occur despite the absence of strongly excluding interactions ($A^*_{ij} \geq -1$): we are assembling only coexisting pairs. Thus, large ϕ alone indicates strong indirect effects that invalidate predictions based solely on pairwise coexistence. Conversely, communities formed from strong competitors may still coexist via emergent coexistence (main text), but high ϕ implies that it is not possible to identify such stabilizing combinations from pairwise data alone.

2. Test 2: Predictability Under Measurement Error

To assess error propagation, we reuse the same sampled subsets and true interaction matrices A^*. We introduce measurement noise by replacing each entry with

$$A^*_{ij} \;\to\; A^*_{ij} + \epsilon_{ij},$$

where $\epsilon_{ij} \sim \mathcal{N}(0, \sigma)$, with $\sigma = 0.1$ unless otherwise stated. For each perturbed matrix:

1. We predict coexistence testing if the row sums of $(I - A^* + \epsilon)^{-1}$ are all positive.
2. We compare this prediction to the true coexistence obtained from asserting if the row sums of $(I - A^*)^{-1}$ are all positive.

A prediction is counted as correct (1) if both true and noisy matrices yield coexistence; otherwise it is marked as a failure (0). We also compute the condition number $\kappa(I - A^*)$, which quantifies sensitivity of the inverse to perturbations. Rolling-mean smoothing (as in Test 1) is used to obtain coexistence probability as a function of κ.

Predictability is strongly governed by the condition number (Fig. LB). For $\kappa \approx 1$, predictions based on noisy matrices are almost always correct. Accuracy decreases sharply with κ: by $\kappa \approx 20$, nearly 90% of predictions fail for $\sigma = 0.1$, with communities predicted to coexist but not doing so under the true dynamics.

Predictability depends on measurement precision. For $\sigma = 0.01$, communities with $\kappa \approx 20$ achieve $\sim 50\%$ accuracy, compared to $\sim 10\%$ for $\sigma = 0.1$. Thus, κ provides a practical indicator of whether coexistence predictions are robust to measurement error, and should be reported when studying communities with strong or heterogeneous interactions.

Test 1 demonstrates that ϕ quantifies the breakdown of extrapolating from pairwise coexistence to community-level coexistence. Test 2 shows that κ determines the reliability of coexistence predictions under measurement noise. Together, these quantities help assess when empirical predictions based on pairwise interactions are likely to succeed or fail.

H. Stability and feedback loops

We summarize here the numerical procedures used to evaluate stability boundaries for the communities obtained through dynamical integration or stable-subset sampling (Aguadé-Gorgorió *et al.*, 2024; Aguadé-Gorgorió and Kefi, 2024). Our goal is to characterize how aggregate interaction statistics constrain stability and to compare numerical results with classical random matrix predictions.

1. Random-Matrix Stability Bounds

May's analysis (May, 1972) provides a statistical condition for linear stability of large random matrices. In the GLV framework, this yields a lower bound on the mean interaction strength μ^* for a fully connected community of size S^*:

$$\mu^* > \min(\mu^*) = \sqrt{\frac{S^*}{2}}\sigma^* - 1, \tag{63}$$

valid in the large-diversity regime ($S^* \gtrsim 50$) (Bunin, 2017; Mallmin *et al.*, 2024).

For smaller communities, a numerical correction was proposed in (Aguadé-Gorgorió and Kefi, 2024):

$$\mu^* > \min(\mu^*) \approx \frac{(S^*)^{1.08}}{14.118}\,\sigma^* - 1. \tag{64}$$

These expressions define statistical (not exact) thresholds for stability.

2. Distance to the Stability Boundary

To evaluate how real communities compare to these theoretical limits, we compute the distance

$$d = |\min(\mu^*)| - |\mu^*|.$$

Positive d indicates that the community lies within the stability domain predicted by random matrix theory (interactions weaker than the predicted threshold). Negative d indicates stronger competitive interactions than the boundary allows.

As shown in Fig. M and discussed below, many low- and moderate-diversity communities lie beyond the random-matrix boundary (i.e., $d < 0$), demonstrating that small systems frequently achieve stability despite interaction strengths exceeding the theoretical statistical limit.

To assess why small communities ($S^* \ll 50$) remain stable under strong competition, we apply the Routh–Hurwitz (RH) criteria to the Jacobian matrix J (Bodson, 2020; Routh, 1877; Toni, 2014). The RH criteria provide necessary and sufficient conditions for all eigenvalues of J to have negative real parts (Levins, 1974). For a community of size S^*, the characteristic polynomial is

$$\det(J - \lambda I) = (-1)^{S^*}\lambda^{S^*} + C_1\lambda^{S^*-1} + \cdots + C_{S^*} = 0, \tag{65}$$

where $J_{ij} = \partial(dx_i/dt)/\partial x_j|_{x^*}$ and, for the GLV system, $J_{ij} = r_i x_i^* A_{ij}$.

Each coefficient C_k is a sum of products of closed feedback loops of length k formed by Jacobian entries (Levins, 1974; Neutel *et al.*, 2002). For $S^* = 3$:

$$\begin{aligned}
C_1 &= J_{11} + J_{22} + J_{33}, && (66)\\
C_2 &= J_{12}J_{21} + J_{13}J_{31} + J_{23}J_{32} - J_{11}J_{22} - J_{11}J_{33} - J_{22}J_{33}, && (67)\\
C_3 &= J_{12}J_{23}J_{31} + J_{13}J_{32}J_{21} + J_{11}J_{22}J_{33} && (68)\\
&\quad - J_{11}J_{23}J_{32} - J_{22}J_{13}J_{31} - J_{33}J_{12}J_{21}. && (69)
\end{aligned}$$

The RH conditions require positivity of the Hurwitz determinants Δ_i constructed from the coefficients C_k (Clark, 1992):

$$\begin{aligned}
\Delta_1 &= C_1 < 0, && (70)\\
\Delta_2 &= C_1C_2 - C_3 > 0, && (71)\\
\Delta_3 &= \begin{vmatrix} C_1 & C_3 & C_5 \\ C_0 & C_2 & C_4 \\ 0 & C_1 & C_3 \end{vmatrix} < 0, && (72)
\end{aligned}$$

with $C_0 = (-1)^{S^*}$. These determinants quantify the relative contribution of negative (short) and positive (long) feedback loops (Neutel *et al.*, 2002, 2007), although for multispecies systems, the combination of weights is far from the trivial “long positive loops destabilize” or “short negative loops stabilize” concepts.

Applying these criteria to all sampled communities (Fig. M), we find that each stable community satisfies all RH determinants as expected from the fact that these are necessary conditions for stability. This demonstrates that small systems can stabilize through detailed feedback structure rather than bulk statistical properties, consistent with earlier analyses (Bodson, 2020; Dambacher *et al.*, 2003).

Because J depends on abundances ($J_{ij} = r_i x_i^* A_{ij}$), stability reflects abundance-weighted interaction pathways rather than A alone. These results highlight the importance of explicit RH-based analysis for understanding stability in moderate-sized ecological networks (Levins, 1974; Neutel *et al.*, 2002).

We evaluate how classical and small-matrix stability criteria described above apply to the GLV equilibria sampled in this study. Section I.J introduced May's complexity–stability limit (Aguadé-Gorgorió and Kefi, 2024; Bunin, 2017; Mallmin *et al.*, 2024; May, 1972), which sets the maximum combination of interaction strength, heterogeneity, diversity, and connectivity (the community's "complexity", (Zelnik *et al.*, 2024)) that ensures linear stability of large random communities. Because this limit is derived for large matrices ($S^* \approx 50$ for $C = 1$, (Aguadé-Gorgorió and Kefi, 2024)), we also considered a small-matrix correction that shifts the threshold upward for moderate richness ($S^* \approx 10$). In addition, we use the Routh–Hurwitz (RH) stability criteria, which provide an exact condition on the coefficients of the Jacobian's characteristic polynomial (Dambacher *et al.*, 2003; Levins, 1974; Neutel *et al.*, 2002).

We analyzed 10^7 stable GLV equilibria sampled within the domain of Fig. 2A. Communities without emergent coexistence (EC) are shown in gray; EC communities (i.e. at least one excluding pair with $A^*_{ij} < -1$ or $A^*_{ji} < -1$) are in purple. Stability was verified directly from the eigenvalues of the Jacobian at equilibrium. Our goal is to compare how May's limit, its small-matrix correction, and the second RH determinant behave for diversities relevant to microbial systems (Chang *et al.*, 2023; Friedman *et al.*, 2017; Venturelli *et al.*, 2018).

Figure MA shows that many small communities exceed May's predicted upper bound for competition strength. The observed interaction mean $|\mu^*|$ frequently lies above the theoretical threshold $|\mu^c|$, indicating that May's limit does not constrain stability for low richness. This is expected: the complexity–stability argument concerns very large ecosystems, whereas in small systems the realized interaction matrices A^* are not random draws from the full interaction pool. Microscopic structure-pair-level asymmetries, sparse subgraphs, and EC motifs-matters.

Figure MB shows that the small-matrix correction more closely matches the numerical results: very few small communities exceed this corrected threshold. However, because this correction relies on statistical averages, it remains approximate and does not fully capture the role of structured interactions in small systems.

Figure MC reports the sign of the second RH determinant (Section I.K), which corresponds to a constraint on third-order feedback loops in the Jacobian (Levins, 1974). A positive determinant indicates that these higher-order loops are weaker than the net balance of first- and second-order contributions. Consistent with earlier work (Neutel *et al.*, 2002), all stable communities-both with and without EC-satisfy this criterion. When this determinant is positive, the remaining RH conditions are typically satisfied as well (not shown), matching previous analyses of empirical and theoretical food webs (Dambacher *et al.*, 2003; Neutel *et al.*, 2002).

These results highlight that stability in small communities is governed not by global random-matrix thresholds but by structured interaction patterns that prevent long competitive loops from overwhelming self-regulation or low-order interactions. This parallels recent work identifying coexistence fingerprints in A^* (Barbier *et al.*, 2021). Testing whether empirical estimates of A^* and x^* (and thus J^*) satisfy RH conditions would be valuable, though such analysis lies beyond the scope of this study.

Figure N summarizes the relationship between May's threshold and the RH condition for com-

munities with $3 \leq S^* \leq 15$. Stable communities (purple), including those with EC, frequently exceed May's predicted bound on competition strength but always satisfy the RH feedback condition, whereas unstable communities (gray) appear below the RH criterion. This reinforces that small-system stability is better captured by feedback-structure constraints than by large-system random matrix theory.

I. Measuring competitive hierarchies and intransitivity (Figure 4)

Competitive intransitivity refers to multispecies competition structures where no single, consistent ranking of competitors exists (Allesina and Levine, 2011; Gallien *et al.*, 2017; Laird and Schamp, 2006; Levine *et al.*, 2017; Soliveres *et al.*, 2015). Early work linked competitive exclusion to strict hierarchies with inevitable single-species dominance (Hardin, 1960), motivating interest in interaction structures that weaken or break this hierarchy. Several approaches quantify intransitivity (Gallien *et al.*, 2017; Koch *et al.*, 2023); here we focus on metrics that use only binary competition outcomes (coexistence vs. exclusion), as these are robustly measurable and widely available (Chang *et al.*, 2023). Following (Chang *et al.*, 2023; Higgins *et al.*, 2017), we evaluate (i) Low Rank Exclusions (LRE) and (ii) Rock-Paper-Scissors (RPS) triplets, and briefly discuss an additional tournament-based metric (Laird and Schamp, 2006).

1. Competitive ranks and Low Rank Exclusion

We assign each species i a competitive rank

$$R_i = \frac{\text{wins} - \text{losses}}{\text{total interactions}}, \tag{73}$$

where wins and losses are determined from the tournament matrix (pairwise exclusion or coexistence). Positive ranks correspond to species that frequently exclude others; negative ranks correspond to species that are frequently excluded.

We then count all one-way exclusionary interactions, defined as pairs where exactly one of A_{ij} or A_{ji} is < -1. Cases of bistability—both A_{ij} and $A_{ji} < -1$—are excluded because the winner depends on initial conditions (Aguadé-Gorgorió *et al.*, 2024; Wright and Vetsigian, 2016). These bistable cases are rare and do not materially affect results.

Among all one-way exclusions, we compute the fraction in which the excluder has a *lower* rank than the excluded species (Low Rank Exclusions). Note that this fraction is taken relative to the number of one-way exclusions only, not all interactions. As discussed below, LRE events require a minimum number of exclusions to establish meaningful rank differences. After correcting for this constraint, LRE fractions remain very small in the random model, consistent with experimental observations (Chang *et al.*, 2023; Higgins *et al.*, 2017).

2. Competitive triplets and Rock-Paper-Scissors

RPS motifs represent the simplest intransitive structure: species A excludes B, B excludes C, and C excludes A (Allesina and Levine, 2011; Kerr *et al.*, 2002; Sinervo and Lively, 1996). Despite their prominence in theory, the frequency and ecological relevance of RPS cycles in species-rich systems remain unclear (Levine *et al.*, 2017).

To quantify RPS motifs, we identify all triplets in which *all* three species pairs exhibit one-way competitive exclusion (following (Chang *et al.*, 2023; Higgins *et al.*, 2017)). Within these "competitive triplets," RPS motifs are those where each species excludes one member and is excluded by another.

The null expectation for RPS frequency is 25%: each competitive triplet contains 3 exclusionary and 3 non-exclusionary directions, yielding 2 RPS configurations and 6 non-RPS configurations. As discussed in Section II.D.2, simulations confirm that stable coexistence in competitive triplets requires one-way exclusions. We compare empirical fractions to this null expectation in the main text.

3. Other metrics of intransitivity

Laird and Schamp (Laird and Schamp, 2006) introduced metrics based solely on tournament outcomes (binary exclusion or coexistence). Their approach assigns each species a number of "wins" (competitive exclusions), analogous to counting $A_{ij} < -1$ elements in each column of A^*. The standard deviation of this win vector reflects the strength of competitive hierarchy.

To contextualize σ_{obs}, they compare it to a homogeneous reference vector (σ_{min}) and a highly heterogeneous vector (σ_{max}), producing a relative transitivity index:

$$\frac{\sigma_{obs} - \sigma_{min}}{\sigma_{max} - \sigma_{min}}. \tag{74}$$

Values near 0 indicate highly intransitive tournaments; values near 1 indicate strong hierarchy.

Communities with few exclusions collapse the lower and upper reference bounds. To maintain interpretability, we also consider:

$$\frac{\sigma_{obs}}{\sigma_{max}}, \tag{75}$$

which equals 1 under perfect hierarchy. Below we analyze both metrics and shows that emergent coexistence does not require strong intransitivity. We do not analyze metrics requiring interaction strengths (Feng *et al.*, 2020), as such data are not consistently available.

4. Building Figure 4

To compute statistics for Figure 4, we sampled a large number of stable states using 10^7 random initial conditions across the parameter range shown in Figure 2A. For each stable community

exhibiting emergent coexistence (EC), we recorded its number of surviving species S^*, the fraction of RPS triplets, and the fraction of LRE events.

Panels A and C show, for each S^*, the mean and $\pm 0.25\sigma$ range of RPS and LRE fractions across all sampled communities. Only a small number of communities with high diversity contain many exclusionary interactions (see Fig. 2C), requiring large simulation effort. We plot $\pm 0.25\sigma$ instead of $\pm 1\sigma$ for clarity; substantial variability persists except at $S^* = 3$, indicating no strong selection for intransitive motifs.

Panels B and D estimate the empirical probability distributions of RPS and LRE fractions expected in an observational study. Because many communities contain very few competitive triplets or exclusions, averaging per-community fractions yields nearly binary distributions and is not informative. Instead, we pool information across communities.

For each diversity level (e.g., $S^* = 8$), we define an "experimental observation" as the mean RPS or LRE fraction across a random sample of 100 communities. We generate 100 such observations to approximate the distribution expected in studies with many recorded interactions (e.g., 77 triplets in (Chang *et al.*, 2023)). We estimate probability densities using kernel density estimation (KDE) via `seaborn.kdeplot` (Chen, 2017), providing continuous distributions without parametric assumptions.

5. Low-rank exclusions require four exclusions and remain rare

Figures 4A,B in the main text show that low-rank exclusions (LREs) represent only $\sim$ 2–3% of all exclusions in stable EC communities with $S^* \gtrsim$ 5–6, consistent with experimental reports (Chang *et al.*, 2023; Higgins *et al.*, 2017). This low frequency arises because a single LRE requires at least four exclusionary interactions (Fig. O). Figure P compares the uncorrected LRE metric with the corrected version used in the main text, which conditions on communities containing at least four exclusionary edges.

Our results show that lower-ranked species can occasionally exclude higher-ranked ones in moderate-size systems (Fig. PA). However, if the four-exclusion requirement is ignored, most communities contain too few exclusionary interactions to permit any LREs, giving a misleading impression that LREs are absent (Fig. PA). In reality, what is absent in such communities is the four-exclusions motif itself. For example, communities with $S^* = 3$ cannot display LREs by construction, even though they may be fully intransitive via an RPS loop. Thus, the LRE metric is not a reliable index of intransitivity in small communities.

Both the unconditioned and conditioned analyses (Fig. PA,B) show that LREs are extremely uncommon for larger S^*, in agreement with empirical observations (Chang *et al.*, 2023; Higgins *et al.*, 2017). Because experimental analyses did not impose the four-exclusion requirement, their results correspond more closely to the very low unconditioned LRE fractions in Fig. PA. The conditioned version shown in the main text therefore provides a clearer description of how intransitive signatures decay with diversity.

6. Rock–paper–scissors loops converge to the random expectation

Figure 4C shows the fraction of rock–paper–scissors (RPS) triplets among all triplets connected by exclusion. If RPS loops were necessary for coexistence, they should be prevalent, as in the $S^* = 3$ case where an RPS loop is the only mechanism preventing dominance when all pairs exclude each other (Fig. 4C in the main text, dotted circle) (Gilpin, 1975; May and Leonard, 1975). Instead, the frequency of RPS triplets declines with increasing S^*.

Because the metric is defined only on triplets connected by pairwise exclusion, it is not affected by the structural limitation identified for the LRE metric. The measured fraction approaches the null expectation obtained by shuffling A^* (gray points, Methods; (Chang *et al.*, 2023)), where 2 of the 8 possible oriented triplets are RPS, giving a baseline of 1/4. In simulations this limit is approached from above; at $S^* = 12$ the RPS frequency is still slightly higher than 25%, likely reflecting the continued relevance of small RPS motifs in moderately sized communities. This residual elevation is expected to vanish for larger S^*.

The null expectation clarifies a numerical error in (Chang *et al.*, 2023). The probability that a single connected triplet is *not* RPS is 0.75. Thus, the probability that none of 77 triplets is RPS is $0.75^{77} = 2.4 \times 10^{-10}$, not 0.25^{77}. Although still extremely small, this value is 37 orders of magnitude larger than previously reported in (Chang *et al.*, 2023). In any case, the persistent empirical observation of EC without RPS motifs requires an explanation, which we attribute in the main text to hierarchical competition.

7. Equivalent results using the Laird–Schamp index

Above we have discussed the Laird–Schamp index (Laird and Schamp, 2006), which quantifies intransitivity using the standard deviation of win counts in a tournament network. The original normalized form,

$$\frac{\sigma_{\rm obs} - \sigma_{\rm min}}{\sigma_{\rm max} - \sigma_{\rm min}},$$

is ill-defined in communities with very few exclusions, since $\sigma_{\rm max} = \sigma_{\rm min}$ when only a single competitive pair exists. In Fig. Q we compute the index when defined, and compare it to the simpler ratio $\sigma_{\rm obs}/\sigma_{\rm max}$, which lacks a well-defined zero baseline but remains computable across a wider domain.

In both cases, intransitivity declines with increasing diversity (Fig. Q), consistent with the results above and with the main-text interpretation: in small systems, intransitive motifs are often required to prevent dominance; in larger systems, numerous indirect pathways reduce this requirement, making intransitivity a secondary byproduct of interaction structure rather than a driver of coexistence. In highly structured limits (nested matrices, resource competition), intransitive signatures may disappear entirely.

J. On the role of heterogeneous growth rates

Throughout the main text and all analyses above we have focused on using homogeneous growth rates, following on equation (3). This has allowed us to understand how the statistics of species interactions allow and modulate the presence of EC, the fraction of excluding pairs in EC communities, their collectivity and the presence or absence of intransitive structures. This provided a framework to understand and discuss emergent coexistence in its simplest form. Here we show that assuming species growth rates to be heterogeneous does not change the main results and overall conclusions of our work. In particular, we want to study the same questions as above but for the equation

$$\frac{dx_i}{dt} = r_i x_i \left(1 - x_i + \sum_{j \neq i}^{S} A_{ij} x_j \right). \tag{76}$$

To maintain homogeneity with how we define A_{ij}, here growth rates r_i will also be random variables sorted from a gaussian distribution $\mathcal{N}(1, \sigma_r)$. Assuming $\mu_r = 1$ simply emerges from rescaling time correspondingly, so that we can focus on σ_r, the standard deviation or heterogeneity of growth rates across species, as a proxy for example of their adaptation to a given environment or resource irrespective of other species. Here we will study two scenarios: one in which we fix $\sigma_r = 0.5$, and another where we equate $\sigma_r = \sigma_A$, which corresponds to a possibly more realistic scenario where the heterogeneity of species in an environment is equivalent to the heterogeneity of their interactions. We refer the reader to (Pearl Mizrahi *et al.*, 2025) for interesting recent results discussing the role of considering heterogeneous carrying capacities instead of heterogeneous growth rates.

The first important point is that, throughout our text, coexistence of otherwise excluding species follows from solving the expression within parenthesis

$$\left(1 - x_i + \sum_{j \neq i}^{S} A^*_{ij} x_j \right) = 0 \quad \text{for a set of surviving species.} \tag{77}$$

This means that, regarding coexistence, growth rate heterogeneity will not change which species are able to counterbalance their interactions to survive at positive abundance. This also means that the results related to collectivity and condition number of A^* are also consistent when using $\sigma_r > 0$.

Instead, what changes is the stability of these coexisting states. It is easy to show that the elements of the Jacobian matrix considering the coexisting states is

$$J_{ij} = r^*_i x^*_i (-I + A^*)_{ij}. \tag{78}$$

Note again that writing $(-I + A^*)$ is simply a redundancy that stems from the fact that, in our notation and that of (Zelnik *et al.*, 2024), A does not contain self-regulation but zeros in the diagonal, so that self regulation normalized to -1 needs to be written separately. In any case, from (84) it is clear that heterogeneous growth rates can affect the stability of coexisting communities. Throughout this SM we have discussed how interaction heterogeneity imposes a boundary, predicted originally by May for linearized systems around an equilibrium, beyond which multispecies communities are no longer stable (Bunin, 2017; May, 1972). Given the off-diagonal elements of the Jacobian $r^*_i x^*_i A^*_{ij}$, it is easy to see that σ_r might result in an increase of the

overall standard deviation of the elements of the Jacobian. The variance of the elements $r_i^* A_{ij}^*$ that multiply x_i^* in the Jacobian results from the variance of the product between two Gaussian distributed variables, which is

$$\mathrm{var}(r_i^* A_{ij}^*) = (\sigma_A^*)^2(1 + (\sigma_r^*)^2) + (\mu_A^*)^2(\sigma_r^*)^2 \tag{79}$$

Increasing the heterogeneity of growth rates will therefore result in increased heterogeneity of the elements of the Jacobian, reducing the likelihood of multispecies coexistence for larger ensembles. We will study here the impacts of modulating σ_r with simulations, and leave for future work incorporating the maths into our analytical predictions.

The central message for the results shown below is that, for small matrices, the heterogeneity of r_i implies that subsets of species can coexist by selecting similar or lower r_i values, resulting in the possibility of sustaining higher competition. Yet, for larger communities, r^* and A^* approach the statistics of the original r and A ensembles (Aguadé-Gorgorió and Kefi, 2024). Lucky subsets will be rarer, and the overall statistics above will dominate, resulting in less stable communities with many species.

We first test how coexistence and stability are affected by increasing σ_r. To do so, we sample a large set of subsets of different species number from random matrices, and evaluate the fraction of subsets that lead to coexisting and stable+coexisting communities (Fig. R). As expected, we find that coexistence is not affected by growth rate heterogeneity, because it only requires that interactions are balanced. Yet, the likelihood of finding stable states decreases because of the form of the Jacobian and the impacts of σ_r on its variance. In this context, we refer the reader to (Gibbs *et al.*, 2018) for a detailed discussion on the decoupling between feasibility and stability conditions (Fig. R) within the framework of random matrix theory.

Next we explore the impacts of σ_r on the presence and fraction of excluding pairs in stable communities and their resulting collectivity, mirroring the central results of Figure 2A, 2C and 3A of the main text. We can see in figure S that, for both $\sigma_r = \sigma_A$ and $\sigma_r = 0.5$ schemes, the qualitative results highlighted in the main text are maintained: EC communities are very common within a high heterogeneity domain, the fraction of excluding pairs in these communities is consistent with analytical expectations and experimental results, and the collectivity metrics increase with diversity and are typically much above $\phi = 1$. The only clear qualitative difference imposed by σ_r can be better observed in figure SD-F in the domain of low σ_A but high $\sigma_r = 0.5$, corresponding to homogeneous interactions but heterogeneous growth rates (possibly an unrealistic scenario, yet still interesting to understand the subtle role of σ_r). In this domain, small communities with EC can survive at high competition even if the random model would predict competitive exclusion and a single dominant species. A possible explanation is that, despite strong competition, a few strong competitors can coexist thanks to having particularly low r_i. This ensures the possibility of a trade-off between competition and growth that later disappears for higher species diversity. This is best observed in figure SE,F: small communities can sustain stronger competition and hence stronger collectivity than $\sigma_r = 0$ case of the main text, thanks to selection for lower r_i values. Yet, as diversity increases, the statistics of the surviving subset approach those of the original r and A: communities have more heterogeneous Jacobians, and it becomes harder to observe communities with high diversity and strong competition. Despite this subtle variation in the shape of what communities survive and their properties, the central message remains consistent to variations in σ_r: communities with random r and A can have pervasive EC and high collectivity provided that interactions are heterogeneous enough.

K. On the role of migration and reinvasions

A key property of the GLV model here is the absence of species migration. We focus on closed experimental ecosystems where extinct species do not reinvade the community (Chang *et al.*, 2023; Friedman *et al.*, 2017; Venturelli *et al.*, 2018). However, as discussed in (Aguadé-Gorgorió and Kefi, 2024), allowing species to migrate back after extinction changes model properties. This is usually modeled by adding a small migration rate m:

$$\frac{dx_i}{dt} = x_i \left(1 - x_i + \sum_{j \neq i}^{S} A_{ij} x_j \right) + m. \tag{80}$$

In simulations, a similar effect is achieved by imposing a minimal abundance below which species cannot decrease, preventing extinction. We tested subsets for feasibility and stability, yet adding migration requires stable states that also resist invasion by any species from the original pool, even those extinct before. Without migration, states can be invadable by species lost during dynamics or absent initially.

Migration alters the multistability regime where EC occurs, reducing alternative stable states since some become invadable by absent species. As shown in (Mallmin *et al.*, 2024; Arnoulx de Pirey and Bunin, 2024; Roy *et al.*, 2020), this creates dynamics where the community moves between states as new species invade, causing ongoing fluctuations or "pinball" dynamics (Aguadé-Gorgorió and Kefi, 2024; Roy *et al.*, 2020). This regime allows small alternative stable states but makes large communities (e.g., 10-15 species) rarer than in the simulations without migration of our work.

While this may not apply directly to the studied experiments, permanent abundance fluctuations are important in ecosystems like plankton (Mallmin *et al.*, 2024; Roy *et al.*, 2020). The presence of EC with migration appears in Section II.A.5, and the decline of stable states in favor of persistent fluctuations is discussed in (Aguadé-Gorgorió and Kefi, 2024).

FIGURES

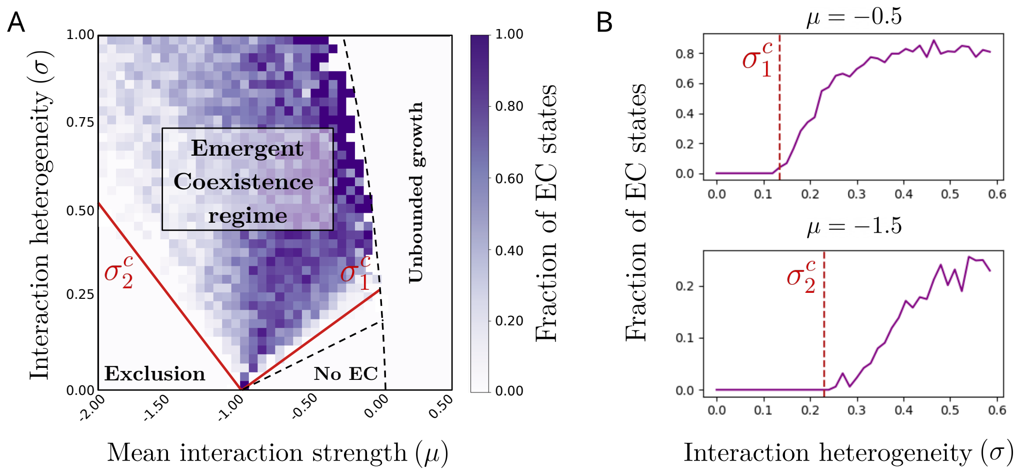

FIG. A **Fraction of EC states over all stable states.** In Fig. 2A,B of the main text, we compare the number of stable EC states over the number of stable states with more than 2 coexisting species. Here we compare the number of stable EC states over the number of stable states, without any restriction on the number of species. The regimes and transitions into an EC domain remain equivalent. However, there appears to be a region close to σ_2^c where EC is very rare at strong competition. Our results in Fig. 2A,B of the main text unveil that EC is not rare per se, but rather that communities with 3 or more species are rare at very strong competition.

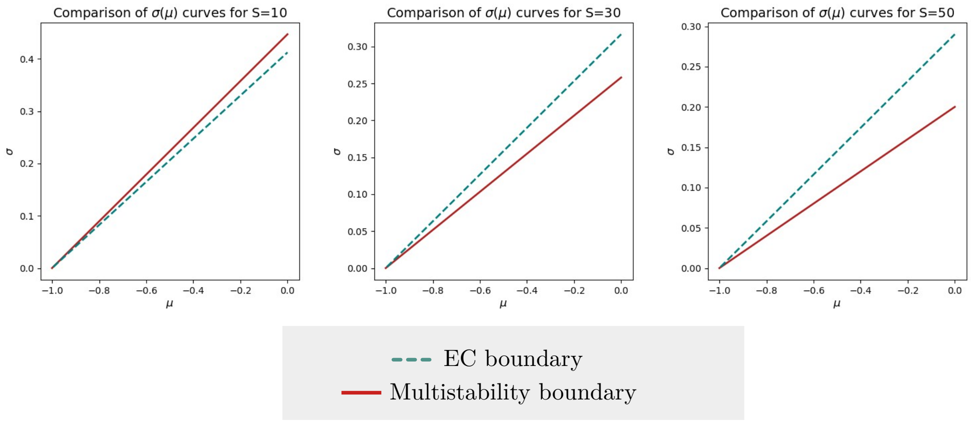

FIG. B **Comparing the EC and multistability boundaries in random uncorrelated matrices.** We compare the multistability boundary found in (Bunin, 2017), which decreases as pool diversity S increases, to the EC boundary found above, which gets sharper yet does not move as S increases. For very small communities that are below the range of validity of the approximations ($S \approx 10$), EC could happen before multistability. For larger communities, we find that the multistability boundary is lower, and hence is crossed at lower σ, than the EC boundary.

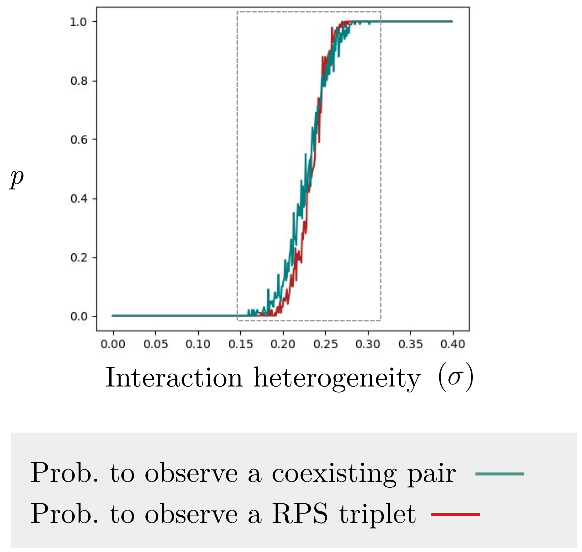

FIG. C **Probability of observing certain motifs in a random matrix.** We simulate 100 matrices with 80x80 elements sampled from $\mathcal{N}(\mu, \sigma)$, with $\mu = -1.5$ and σ increasing (x-axis). We measure across these matrices the fraction of times in which a matrix has at least one coexisting pair ($A_{ij} > -1$, $A_{ji} > -1$, in teal) and the fraction of times a matrix has at least one rock-paper-scissors triplet (in red). Both motifs provide a proxy for the minimal standard deviation σ by which a large matrix will transition from very rare coexistence ($p = 0$) to almost ensured coexistence ($p = 1$). This transition happens quite sharply for both motifs, between $\sigma = 0.2$ and $\sigma = 0.26$, and is consistent with the analytical estimate shown in figure 2B of the main text, bottom panel.

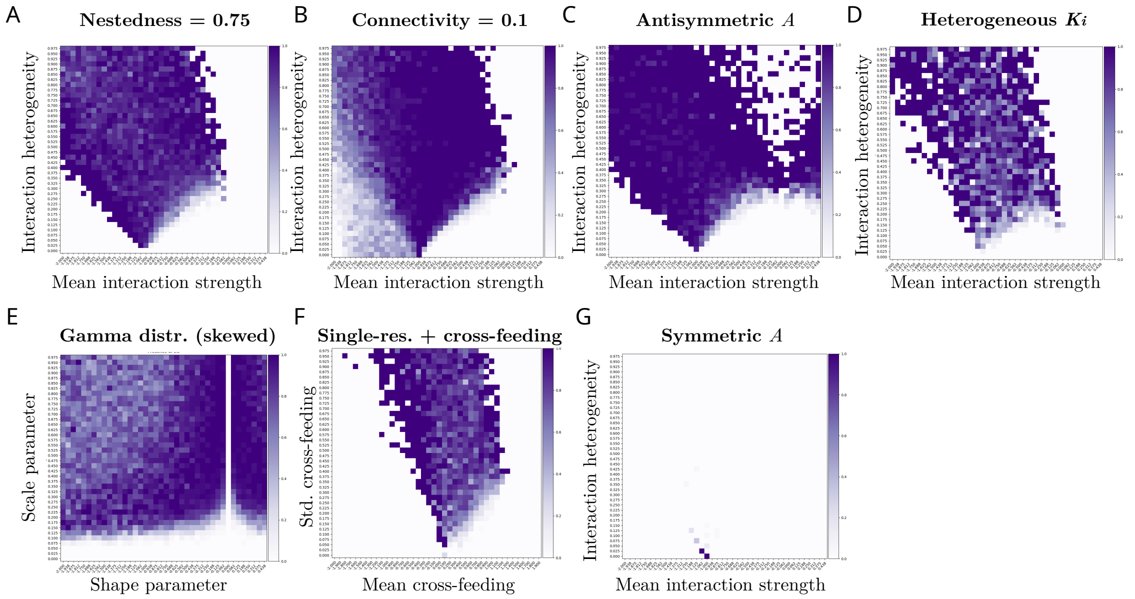

FIG. D **Emergent coexistence under different matrix randomizations.** In purple the fraction of stable states that contain at least one excluding pair, using the equivalent numerical procedure of figure 2A of the main text, but under different types of interaction strength randomizations that are not the fully random, uncorrelated $A_{ij} \in \mathcal{N}(\mu, \sigma)$ elements of the main text. We find that EC is a common event (dark purple, most or all states contain at least one excluding pair) across different matrix randomizations even if the shape of the phase spaces can change depending on the model under study. The only exception is in the limit-case scenario of fully symmetric interactions ($A_{ij} = A_{ji}$) where EC is very rare (panel F). In Section I.L we describe in detail the way each of the randomizations is build and the interpretation of the x- and y-axes and simulation outcomes.

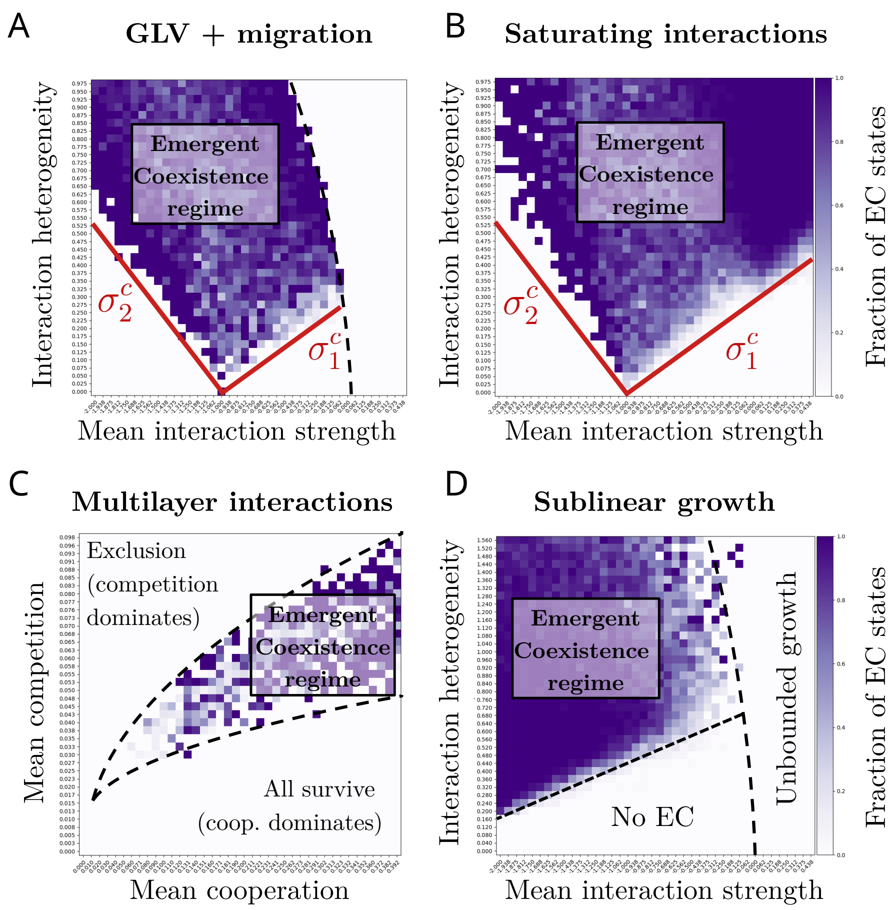

FIG. E **Emergent coexistence in different dynamical models.** See Section II.A.5 for the extended figure caption.

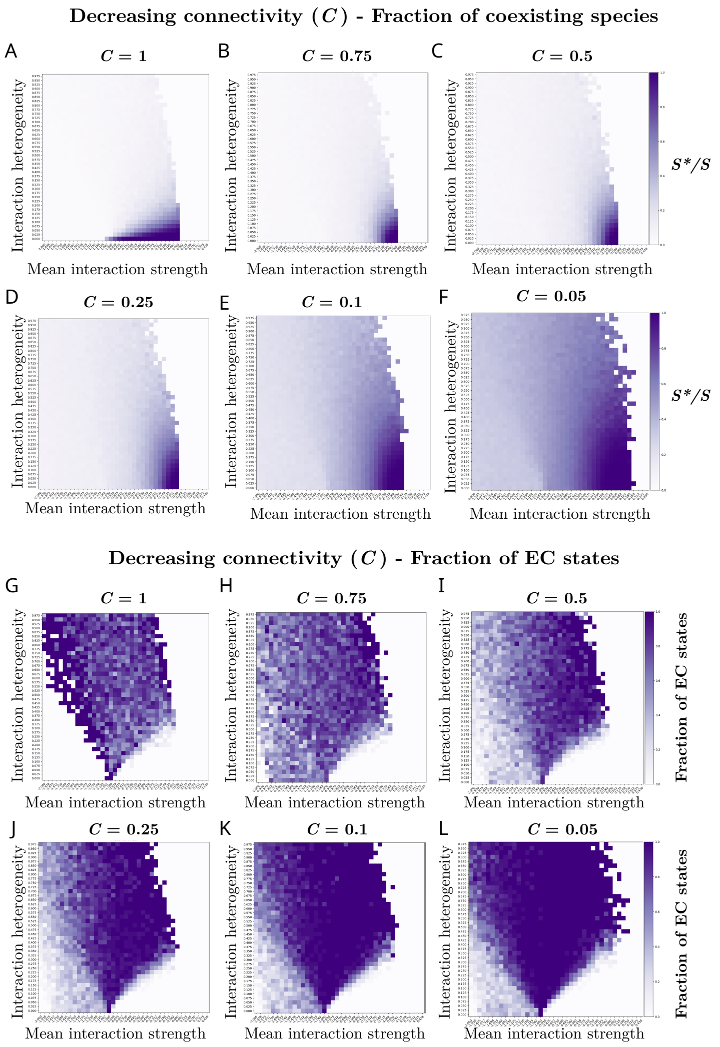

FIG. F **Fraction of surviving species and EC states under decreasing connectivity.** (A-F) Phase space of the GLV model under decreasing connectivity. Note that the μ and σ values do not refer to the mean and variance of the final matrix after the sparsity filter is introduced, but to the values of the original A matrix before it is filled with zeros. Reduced connectivity reduces μ linearly, while it first increases σ and later decreases it. Reductions of connectivity as low as $C = 0.5$ do not result in relevant changes in species abundances, whereas lower connectivity values result in a very homogeneous distribution of effective interactions (most A_{ij} become null), leading to an increase in the diversity of the system as many species do not interact. (G-L) Equivalent simulations as (A-F), studying the fraction of states that contain at least one excluding pair (EC states) out of 100 simulations for each (μ, σ) pair. As connectivity decreases, we can observe that (i) the competitive exclusion phase disappears so that more species can coexist and (ii) the fraction of stable states with EC inside the EC domain approaches 1, meaning that decreased connectivity increases the chances to observe emergent coexistence.

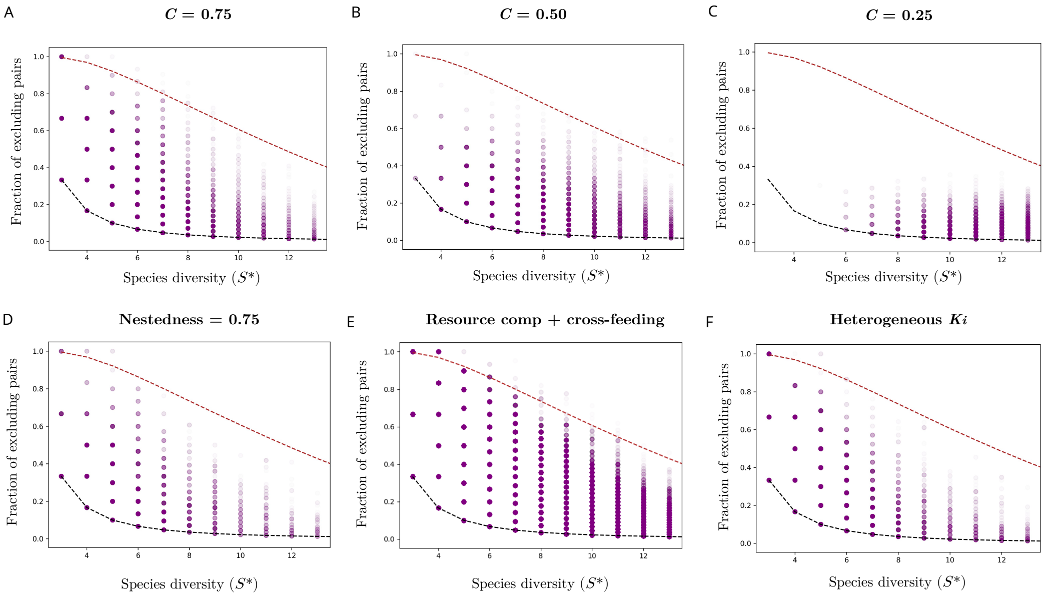

FIG. G **Fraction of excluding pairs under decreasing connectivity and hierarchical interactions**. We replicate the analysis of Fig. 2C in the main text, but for (A-C) decreasing connectivity and (D-F) hierarchical matrices. (A-C) Consistent with the figure above, in which we learn that decreasing connectivity increases the richness of stable communities, we observe that, as we decrease C, it becomes harder to observe small communities with EC, whereas larger communities become more common. In any case, the observed communities still fall within the domains of the analytical estimates predicted in the main text. (D-F) Fraction of excluding pairs for communities with EC (T, B and $\tilde{A}$ matrices respectively). The fraction of excluding pairs follows a qualitatively equivalent pattern to that of the GLV model with random interactions. Note that B has much more data points than A because we implemented an increased simulation span of 50.000 $\tilde{A}_{ij}$ matrices and 100 different initial conditions for each matrix. We leave for future research the detailed analysis of quantitative differences for the EC metrics across different random matrix structures.

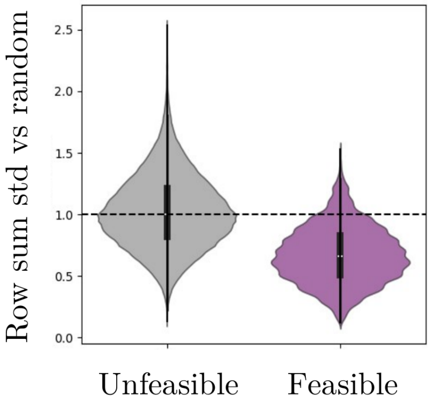

FIG. H **Row sums of coexisting species interaction matrices are homogeneous.** Here we plot the violin plots (using the seaborn.violinplot function in python) of the standard deviation of row sums of a 10^6 matrices of interactions A^* sampled from A with μ and σ sampled randomly within the domain of figure 2A in the main text. We divide the standard deviation by the average standard deviation of 100 randomizations of each matrix generated by shuffling all off-diagonal elements. A value of 1 indicates that the row sums of the interaction matrix are as heterogeneous as the row sums of the randomized versions, whereas a value smaller than 1 indicates that row sums are more homogeneous than the random expectation. Unfeasible communities, where the abundances of some species in a given subset are not positive and hence there is no coexistence, have row sum values similar to those of a random matrix. Feasible communities, instead, have more homogeneous row sums than the random expectation, meaning that there is a tendency or filter for which species can coexist if they all perceive competition $\sum_j A^*_{ij}$ in a similar way.

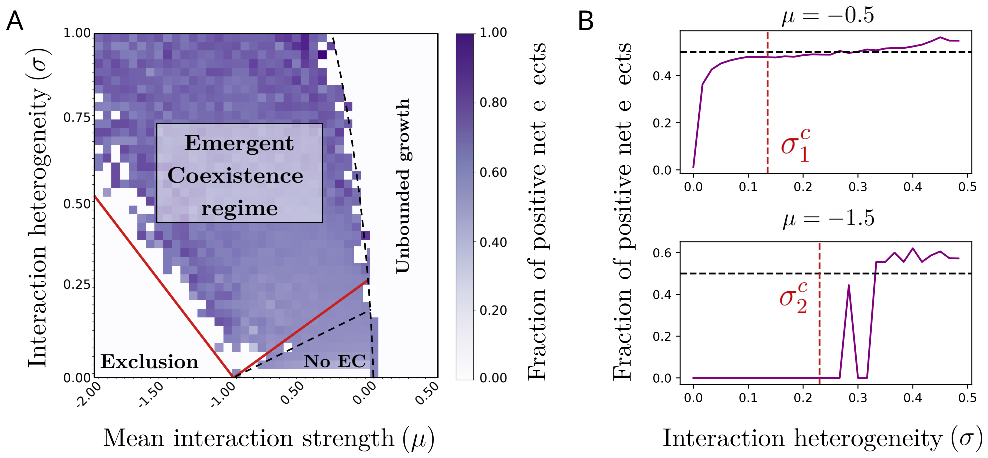

FIG. I **Fraction of positive net effects ($(I - A^*)^{-1}_{ij} > 0)$).** Equivalent plot to figure 2A of the main text but, instead of measuring the fraction of states that harbor emergent coexistence, we measure the fraction of positive elements present in $(I - A^*)^{-1}$ given a stable state with interactions encoded in A^* found after simulating the GLV model with pool interactions $A \sim \mathcal{N}(\mu, \sigma)$. In (A) we plot the phase space, to observe that much before EC appears, the fraction of positive net effects in a community rapidly approaches 0.5, meaning that half of the effects between species are in fact facilitative. Equivalent to figure 2A in the main text, in (B) we plot two vertical slices of (A) for $\mu = -0.5$ and $\mu = -1.5$ and increasing σ.

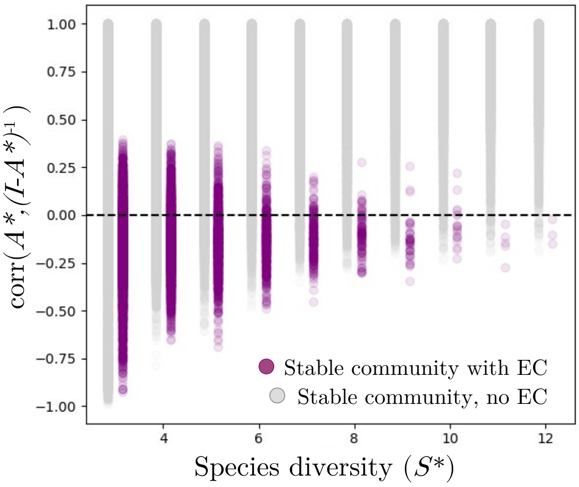

FIG. J **Correlation between direct and net effects.** Consistent with discussions in the main text and Section II.B.2, here we measure the Pearson correlation between the matrix of direct interactions and the matrix of net effects. As expected, we find that in communities without EC (gray) characterized by weak interactions the two matrices correlate quite well, meaning that direct interactions provide certain information on net effects, mainly because long indirect effects are negligible. Instead, for communities with EC (purple) that are characterized by the presence of strong interactions, the two matrices do not necessarily correlate: for small communities, they can be anti-correlated, meaning that direct competitors are in fact the species that are providing a rescue effect. For more diverse communities, the two matrices become uncorrelated, leading to the predictability limit proposed in the main text: direct interactions hold little information about net effects and species abundances, mostly because they are negligible when compared to much longer and heavier chains of indirect effects.

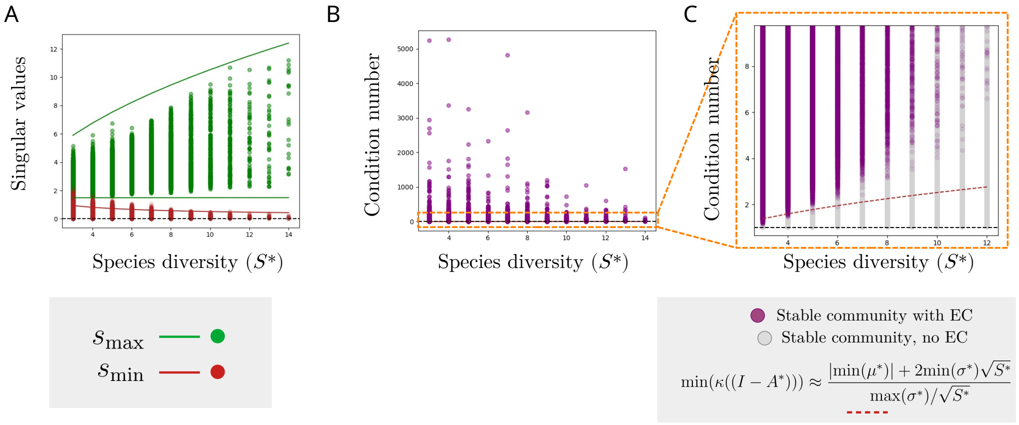

FIG. K **Numerical and analytical predictions for the condition number κ.** In (A) we plot the largest and smallest singular values found for a large number of communities with EC found after simulating 10^6 systems within the range of figure 2A in the main text. The circles are the values for each state computed with `numpy.linalg.svd`, whereas the lines refer to the maximum and minimum predictions for both $s_{\max}$ and $s_{\min}$ from Random Matrix Theory (Section II.B.4). In (B) we plot the condition number for those states that results from dividing $s_{\max}/s_{\min}$, and in (C) we zoom in and also add the values for communities that do not harbor EC, which allows us to both visualize the analytical prediction proposed in Section II.B.4 together with the observation that κ will only remain close to 1 for communities with very weak and homogeneous interaction strengths. As interactions become relatively strong and heterogeneous –as for communities with EC, most communities have a condition number (κ) much larger than 1. The best expectation is that the condition number increases as $\kappa \sim (S^*)$, but most communities fall much above this analytical estimate. The consequence is that small errors in the measurement of A^* can translate to large errors in the prediction of species abundances via matrix inversion.

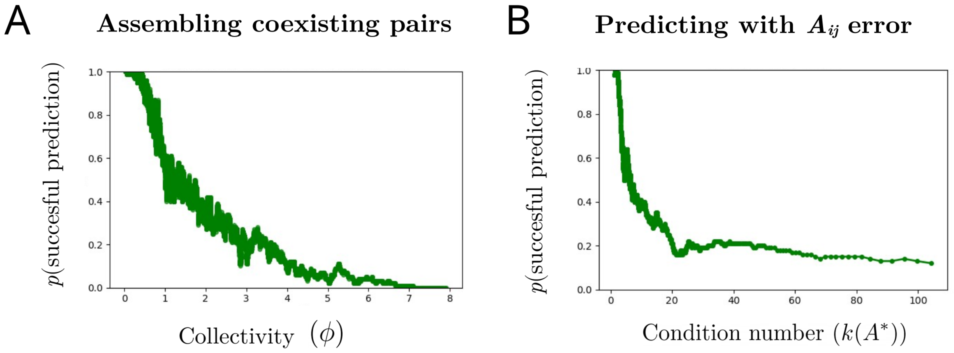

FIG. L **Testing the role of ϕ and κ in possible experimental tests of community assembly.** In (A) we study if assembling pairs of coexisting species will result in a coexisting community as a function of ϕ. As discussed throughout sections I.I and II.B.5, we see how communities with very small collectivity (few species, weak indirect effects) result in a success, meaning that the final community coexists. Yet, as ϕ increases, the likelihood that the reductionist approach succeeds decreases rapidly, so that assembling species that coexist by pairs will not result in a successful, coexisting community. Instead, EC indicates that indirect effects dominate over the community, and assembly tests based on pairwise interactions alone will not succeed. In (B) we test if the predicted coexistence based on imprecise measurements of $A^*_{ij} + \epsilon$, with $\epsilon \sim \mathcal{N}(\mu = 0, \sigma = 0.1)$, successfully predicts the coexistence of the real community with interactions A^*_{ij}. We find that, as condition number increases, small errors in our measurements of species interactions rapidly propagate, and for $\kappa \approx 20$, most of the predicted coexisting communities will not coexist in reality.

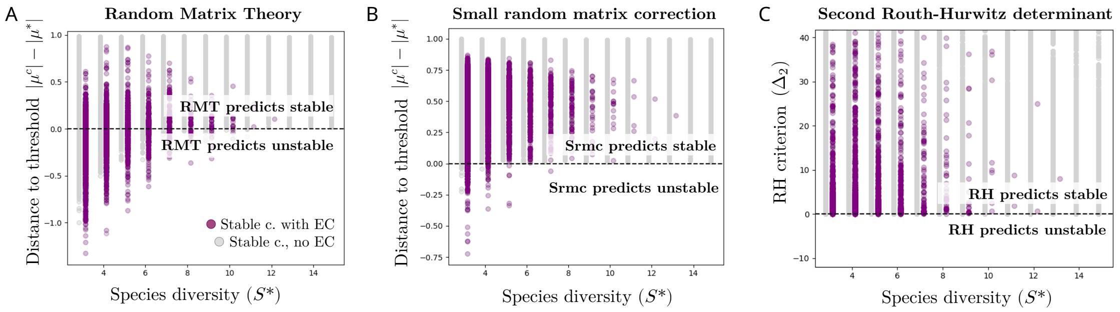

FIG. M **Stability predictions for EC states.** Different stability predictions based on the properties of A^* and J^* for communities with (purple) and without (gray) EC, where communities with EC are in fact communities with strong competition. In (A) we see that many communities have stronger competition than what the Random Matrix Theory threshold predicts (Section I.J). Once we correct the threshold using recent approximations for communities of moderate size in (B), we see that more communities fulfill the stability prediction and have absolute interaction strength weaker than the limit prediction $|\mu^c|$. However, it is clear that for the smallest communities these approximations for large and moderate sized communities do not apply, and more subtle patterns allow the states to maintain stability even under strong competition. These patterns are better captured by the Routh-Hurwitz criteria and in particular the condition on the second determinant (Section I.K). We can see that all communities, with and without EC, fulfill the condition for a positive second RH determinant, mostly because it is not based on aggregate statistics of the community but rather the exact values and locations of interaction strengths.

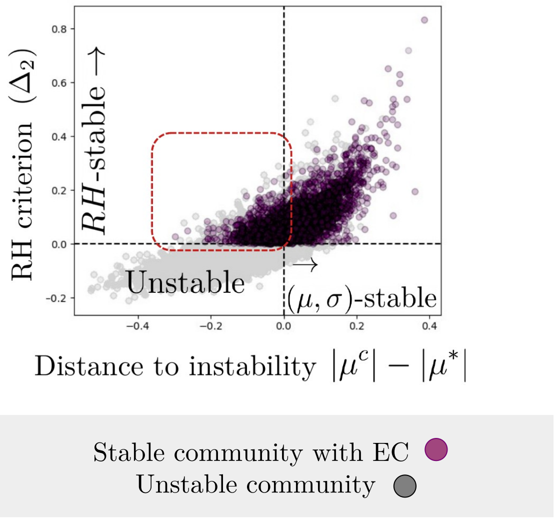

FIG. N **Random matrix theory and Routh-Hurwitz predictions on stability.** Bringing together the results of the figure in the previous page, here we plot the distance to May's threshold (x-axis) and the sign of the second Routh-Hurwitz determinant (y-axis) for stable communities with EC (purple) and unstable communities (gray) sampled from the GLV model within the parameter domain of figure 2A in the main text. While many communities have stronger competition than what the Random Matrix Theory approximation predicts (dashed red square, Random Matrix Theory does not operate for such small communities), they all fulfill the RH criteria, providing a microscopic, interaction-structure explanation for linear stability under strong competition.

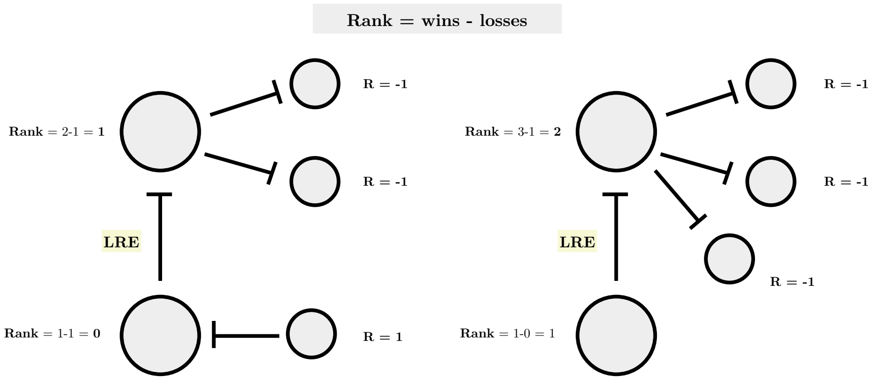

FIG. O **Low rank exclusions require at least four exclusionary interactions.** Circles represent species and arrows represent exclusionary interactions. We do not paint the chains of non-exclusionary interactions that we have shown could lead to species coexistence. In any case, we show that for a LRE to happen, we require a minimum of four exclusionary interactions. Because the higher-ranked species will have at least one loss (that of the LRE), it requires additional wins with other species in the community to qualify as a LRE. This shows that LRE are in fact more complex and specific motifs than what the initial intuition proposes, and they might not be a good metric to assess intransitivity in systems with few species or few exclusionary elements.

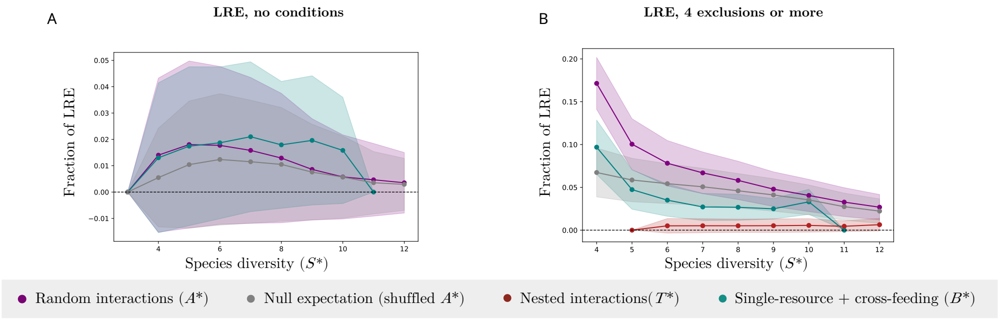

FIG. P **Low rank exclusions without and with the 4-exclusions requirement.** LRE requires at least 4 exclusionary interactions (see figure above). If we do not consider this requirement, the apparent fraction of exclusions that is LRE is extremely low (A), mostly because there are a lot of communities with 3 or fewer exclusions that will never harbor a LRE. Instead, in (B) we plot the figure of the main text that corrects for this requirement, and studies the presence of LRE in systems with 4 or more interactions only. Both for the unconditioned case (A) and for the conditioned case (B), we can see that large communities very rarely harbor LRE's, consistent with empirical observations.

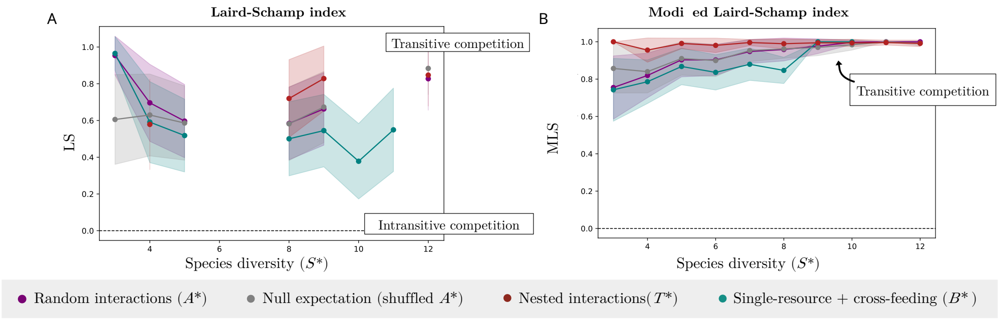

FIG. Q **Laird-Schamp index with and without $s_{\min}$.** The original intransitivity index used in Laird and Schamp (Laird and Schamp, 2006) fails in our model when the divisor $s_{\max} - s_{\min} = 0$, which happens if there is only one exclusionary element in a system and so there is no such maximum or minimum rank heterogeneity. We can correct this by defining a less restrictive index $s_{obs}/s_{\max}$ (Section II.D.3). If this index equals 1, it means that species ranks are distributed in the most hierarchical (heterogeneous) way, indicative of a transitive competition matrix. The results are not surprising and qualitatively equivalent to the two intransitivity indexes presented in the main text, which indicate that clear intransitive motifs disappear with species diversity, where in fact few exclusions are present and many indirect effects explain the survival of these excluded species in the absence of intransitive motifs.

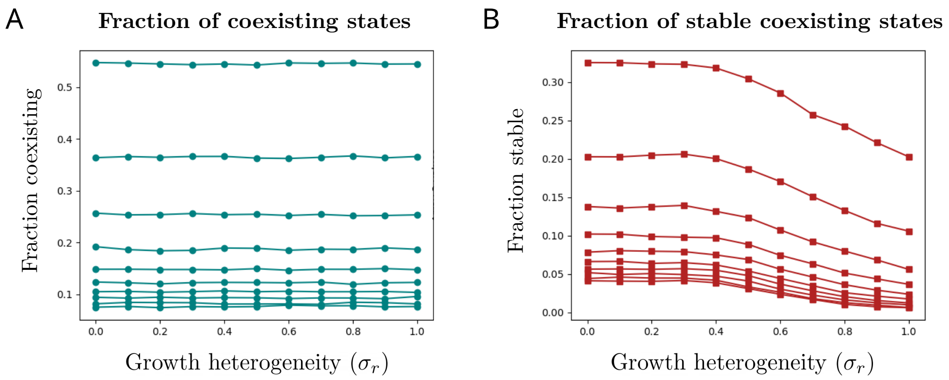

FIG. R **Fraction of coexisting and stable+coexisting states under increasing** σ_r. We generate random matrices A within the domain of figure 2A in the main text and growth rates $r \sim \mathcal{N}(1, \sigma_r)$. For each diversity value between $S^* = 3$ and $S^* = 12$, we sample 50000 subsets of this size from A and r and ask what fraction of all sampled subsets are coexisting (positive abundances, A) and stable (negative eigenvalues, B) communities with EC. As expected from the discussion in section II.E, coexistence is not affected by increasing growth rates, whereas the likelihood of stability decreases because the Jacobian increases in heterogeneity with σ_r.

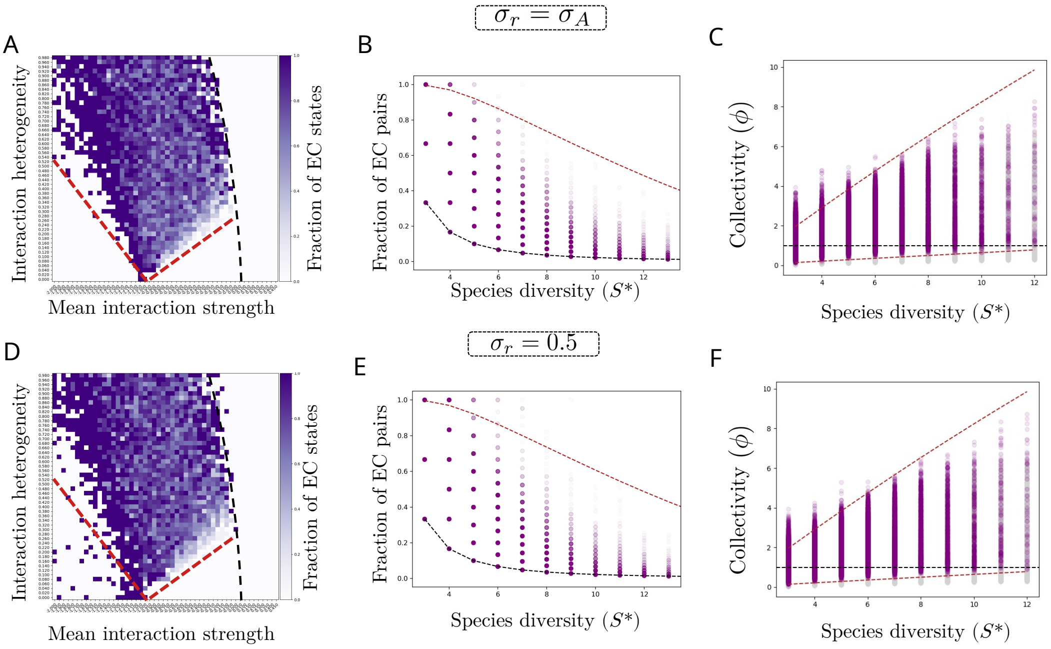

FIG. S **Fraction of communities with EC and fraction of excluding pairs in those communities**. Here we replicate the simulations of figure 2A and 2C in the main text, corresponding to the fraction of communities that harbor EC and the fraction of excluding pairs in those communities, for the case where $\sigma_r = \sigma_A$ (A,B) and the case where $\sigma_r = 0.5$ (C,D). We incorporate the same dashed lines of the original figures, corresponding to analytical predictions for $\sigma_r = 0$ for comparison. The main qualitative observations are maintained when growth rates are heterogeneous across species and are discussed in section II.E.

REFERENCES

Aguadé-Gorgorió, G., J.-f. Arnoldi, M. Barbier, and S. Kéfi (2024), Ecology Letters **27** (4), e14413.

Aguadé-Gorgorió, G., and S. Kefi (2024), Journal of Physics: Complexity.

Aguadé-Gorgorió, G., I. Lajaaiti, J.-f. Arnoldi, and S. Kéfi (2025), Oikos **2025** (1), e10980.

Allesina, S., and J. M. Levine (2011), Proceedings of the National Academy of Sciences **108** (14), 5638.

Allesina, S., and S. Tang (2012), Nature **483** (7388), 205.

Altieri, A., F. Roy, C. Cammarota, and G. Biroli (2021), Physical Review Letters **126** (25), 258301.

Arnoldi, J.-F., M. Barbier, R. Kelly, G. Barabás, and A. L. Jackson (2022), Methods in Ecology and Evolution **13** (1), 167.

Arya, S., A. B. George, and J. O'Dwyer (2025), Current Opinion in Microbiology **83**, 102580.

Arya, S., A. B. George, and J. P. O'Dwyer (2023), Proceedings of the National Academy of Sciences **120** (48), e2307313120.

Barabás, G., M. J. Michalska-Smith, and S. Allesina (2017), Nature ecology & evolution **1** (12), 1870.

Barbier, M. (2025), .

Barbier, M., J.-F. Arnoldi, G. Bunin, and M. Loreau (2018), Proceedings of the National Academy of Sciences **115** (9), 2156.

Barbier, M., C. De Mazancourt, M. Loreau, and G. Bunin (2021), Physical Review X **11** (1), 011009.

Bascompte, J. (2009), Frontiers in Ecology and the Environment **7** (8), 429.

Bascompte, J. (2010), Science **329** (5993), 765.

Bender, E. A., T. J. Case, and M. E. Gilpin (1984), Ecology **65** (1), 1.

van den Berg, N. I., D. Machado, S. Santos, I. Rocha, J. Chacón, W. Harcombe, S. Mitri, and K. R. Patil (2022), Nature ecology & evolution **6** (7), 855.

Billick, I., and T. J. Case (1994), Ecology **75** (6), 1529.

Biroli, G., G. Bunin, and C. Cammarota (2018), New Journal of Physics **20** (8), 083051.

Bodson, M. (2020), IEEE Control Systems Magazine **40** (1), 45.

Bunin, G. (2017), Physical Review E **95** (4), 042414.

Calleja-Solanas, V., N. Khalil, J. Gómez-Gardeñes, E. Hernández-García, and S. Meloni (2022), Physical Review E **106** (6), 064307.

Camacho-Mateu, J., A. Lampo, M. Sireci, M. A. Muñoz, and J. A. Cuesta (2024), Proceedings of the National Academy of Sciences **121** (5), e2309575121.

Castillo-Alvino, H., and M. Marvá (2020), Journal of Biological Dynamics **14** (1), 222.

Castledine, M., J. Pennycook, A. Newbury, L. Lear, Z. Erdos, R. Lewis, S. Kay, D. Sanders, D. Sünderhauf, A. Buckling, *et al.* (2024), Microbiology **170** (9), 001489.

Cenci, S., and S. Saavedra (2018), Physical Review E **97** (1), 012401.

Chang, C.-Y., D. Bajić, J. C. Vila, S. Estrela, and A. Sanchez (2023), Science **381** (6655), 343.

Chen, Y.-C. (2017), Biostatistics & Epidemiology **1** (1), 161.

Chesson, P. (2000), Annual review of Ecology and Systematics **31** (1), 343.

Clark, R. N. (1992), IEEE Control Systems Magazine **12** (3), 119.

Courchamp, F., L. Berec, and J. Gascoigne (2008), *Allee effects in ecology and conservation* (OUP Oxford).

Czárán, T., and E. Szathmáry (2000), The geometry of ecological interactions **116**, 134.

Dal Bello, M., H. Lee, A. Goyal, and J. Gore (2021), Nature Ecology and Evolution **5** (10), 1424.

Dambacher, J. M., H.-K. Luh, H. W. Li, and P. A. Rossignol (2003), The American Naturalist **161** (6), 876.

Demmel, J. W. (1987), Numerische Mathematik **51**, 251.

Domínguez-García, V., V. Dakos, and S. Kéfi (2019), Proceedings of the National Academy of Sciences **116** (51), 25714.

Donohue, I., H. Hillebrand, J. M. Montoya, O. L. Petchey, S. L. Pimm, M. S. Fowler, K. Healy, A. L. Jackson, M. Lurgi, D. McClean, *et al.* (2016), Ecology letters **19** (9), 1172.

Dormand, J. R., and P. J. Prince (1980), Journal of computational and applied mathematics **6** (1), 19.

Dormann, C. F. (2007), Plant Ecology **191**, 171.

Dormann, C. F., and S. H. Roxburgh (2005), Proceedings of the Royal Society B: Biological Sciences **272** (1569), 1279.

Dunne, J. A. (2006), Ecological networks: linking structure to dynamics in food webs **1**, 27.

Edelman, A. (1988), SIAM journal on matrix analysis and applications **9** (4), 543.

El Ghaoui, L. (2002), Linear algebra and its applications **343**, 171.

Engel, E. C., and J. F. Weltzin (2008), Plant Ecology **195**, 77.

Estrela, S., J. C. Vila, N. Lu, D. Bajić, M. Rebolleda-Gómez, C.-Y. Chang, J. E. Goldford, A. Sanchez-Gorostiaga, and Á. Sánchez (2022), Cell Systems **13** (1), 29.

Feng, Y., S. Soliveres, E. Allan, B. Rosenbaum, C. Wagg, A. Tabi, E. De Luca, N. Eisenhauer, B. Schmid, A. Weigelt, *et al.* (2020), Methods in Ecology and Evolution **11** (1), 117.

Fortuna, M. A., D. B. Stouffer, J. M. Olesen, P. Jordano, D. Mouillot, B. R. Krasnov, R. Poulin, and J. Bascompte (2010), Journal of animal ecology , 811.

Friedman, J., L. M. Higgins, and J. Gore (2017), Nature ecology & evolution **1** (5), 0109.

Galla, T. (2018), Europhysics Letters **123** (4), 48004.

Gallien, L., N. E. Zimmermann, J. M. Levine, and P. B. Adler (2017), Ecology Letters **20** (7), 791.

Gibbs, T., J. Grilli, T. Rogers, and S. Allesina (2018), Physical Review E **98** (2), 022410.

Gilbert, G. T. (1991), The American Mathematical Monthly **98** (1), 44.

Gilpin, M. E. (1975), The American Naturalist **109** (965), 51.

Gilpin, W. (2024), arXiv preprint arXiv:2403.19186.

Giral Martínez, J., M. Barbier, and S. De Monte (2024), bioRxiv , 2024.

Goldford, J. E., N. Lu, D. Bajić, S. Estrela, M. Tikhonov, A. Sanchez-Gorostiaga, D. Segrè, P. Mehta, and A. Sanchez (2018), Science **361** (6401), 469.

Grilli, J., M. Adorisio, S. Suweis, G. Barabás, J. R. Banavar, S. Allesina, and A. Maritan (2017a), Nature communications **8** (1), 14389.

Grilli, J., G. Barabás, M. J. Michalska-Smith, and S. Allesina (2017b), Nature **548** (7666), 210.

Grilli, J., T. Rogers, and S. Allesina (2016), Nature communications **7** (1), 12031.

Guimaraes Jr, P. R. (2020), Annual Review of Ecology, Evolution, and Systematics **51** (1), 433.

Hardin, G. (1960), science **131** (3409), 1292.

Hatton, I. A., O. Mazzarisi, A. Altieri, and M. Smerlak (2024), Science **383** (6688), eadg8488.

Higgins, L. M., J. Friedman, H. Shen, and J. Gore (2017), BioRxiv , 175737.

Holling, C. S. (1959), The canadian entomologist **91** (5), 293.

Hu, J., D. R. Amor, M. Barbier, G. Bunin, and J. Gore (2022), Science **378** (6615), 85.

Hutchinson, G. E. (1953), Proceedings of the Academy of Natural Sciences of Philadelphia **105**, 1.

Ives, A. R., and S. R. Carpenter (2007), science **317** (5834), 58.

Jacquet, C., C. Moritz, L. Morissette, P. Legagneux, F. Massol, P. Archambault, and D. Gravel (2016), Nature communications **7** (1), 12573.

Kéfi, S., V. Domínguez-García, I. Donohue, C. Fontaine, E. Thébault, and V. Dakos (2019), Ecology letters **22** (9), 1349.

Kerr, B., M. A. Riley, M. W. Feldman, and B. J. Bohannan (2002), Nature **418** (6894), 171.

Kessler, D. A., and N. M. Shnerb (2015), Physical Review E **91** (4), 042705.

Kessler, D. A., and N. M. Shnerb (2025), Physical Review E **111** (3), 034408.

Koch, F., A.-M. Neutel, D. K. Barnes, and K. T. Allhoff (2024), bioRxiv , 2024.

Koch, F., A.-M. Neutel, D. K. Barnes, K. Tielborger, C. Zarfl, and K. T. Allhoff (2023), Communications Biology **6** (1), 690.

Kvrivan, V., and J. Eisner (2006), Theoretical Population Biology **70** (4), 421.

Laird, R. A., and B. S. Schamp (2006), The American Naturalist **168** (2), 182.

Lajaaiti, I., S. Kéfi, and J.-F. Arnoldi (2024), Proceedings of the Royal Society B **291** (2032), 20240930.

Landi, P., H. O. Minoarivelo, Å. Brännström, C. Hui, and U. Dieckmann (2018), Population ecology **60** (4), 319.

Lee, H., B. Bloxham, and J. Gore (2023), Proceedings of the National Academy of Sciences **120** (35), e2212113120.

Leibold, M., and M. Barbier (2025), .

Lele, K., B. E. Wolfe, and L. H. Uricchio (2024), bioRxiv , 2024.

Levine, J. M. (1999), Ecology **80** (5), 1762.

Levine, J. M., J. Bascompte, P. B. Adler, and S. Allesina (2017), Nature **546** (7656), 56.

Levine, S. H. (1976), The American Naturalist **110** (976), 903.

Levins, R. (1974), Annals of the New York Academy of Sciences **231** (1), 123.

Liautaud, K., E. H. van Nes, M. Barbier, M. Scheffer, and M. Loreau (2019), Ecology letters **22** (8), 1243.

Lubiana Botelho, L., C. Jeynes-Smith, S. A. Vollert, and M. Bode (2025), Ecology Letters **28** (1), e70034.

Mallmin, E., A. Traulsen, and S. De Monte (2024), Proceedings of the National Academy of Sciences **121** (11), e2312822121.

Marcus, S., A. M. Turner, and G. Bunin (2022), PLoS computational biology **18** (7), e1010274.

Marcus, S., A. M. Turner, and G. Bunin (2024), arXiv preprint arXiv:2405.11360.

Martínez, J. G., S. De Monte, and M. Barbier (2024), arXiv preprint arXiv:2411.14969.

May, R. M. (1972), Nature **238** (5364), 413.

May, R. M. (2019), *Stability and complexity in model ecosystems* (Princeton university press).

May, R. M., and W. J. Leonard (1975), SIAM journal on applied mathematics **29** (2), 243.

Mazzarisi, O., and M. Smerlak (2024), Physical Review E **110** (5), 054403.

McCann, K. S. (2000), Nature **405** (6783), 228.

Mehta, P., and R. Marsland III (2021), arXiv preprint arXiv:2110.04965.

Miller, Z. R., and D. Max (2025), Ecology Letters **28** (9), e70206.

Neutel, A.-M., J. A. Heesterbeek, and P. C. De Ruiter (2002), Science **296** (5570), 1120.

Neutel, A.-M., J. A. Heesterbeek, J. Van de Koppel, G. Hoenderboom, A. Vos, C. Kaldeway, F. Berendse, and P. C. De Ruiter (2007), Nature **449** (7162), 599.

Newman, M. (2018), *Networks* (Oxford university press).

Ortiz, A., N. M. Vega, C. Ratzke, and J. Gore (2021), The ISME Journal **15** (7), 2131.

Pasqualini, J., A. Maritan, A. Rinaldo, S. Facchin, E. Savarino, A. Altieri, and S. Suweis (2024), arXiv preprint arXiv:2406.07465.

Payrató-Borras, C., L. Hernández, and Y. Moreno (2019), Physical Review X **9** (3), 031024.

Pearl Mizrahi, S., H. Lee, A. Goyal, E. Owen, and J. Gore (2025), bioRxiv , 2025.

Pennekamp, F., M. Pontarp, A. Tabi, F. Altermatt, R. Alther, Y. Choffat, E. A. Fronhofer, P. Ganesanandamoorthy, A. Garnier, J. I. Griffiths, *et al.* (2018), Nature **563** (7729), 109.

Picot, A., S. Shibasaki, O. J. Meacock, and S. Mitri (2023), Current Opinion in Microbiology **75**, 102354.

Pilosof, S., M. A. Porter, M. Pascual, and S. Kéfi (2017), Nature Ecology & Evolution **1** (4), 0101.

Piñero, J., and R. Solé (2018), Entropy **20** (2), 98.

Arnoulx de Pirey, T., and G. Bunin (2024), Physical Review X **14** (1), 011037.

Poley, L., T. Galla, and J. W. Baron (2025), Physical Review E **111** (1), 014318.

Qian, J. J., and E. Akçay (2020), Nature ecology & evolution **4** (3), 356.

Rao, C., K. Z. Coyte, W. Bainter, R. S. Geha, C. R. Martin, and S. Rakoff-Nahoum (2021), Nature **591** (7851), 633.

Rohr, R. P., S. Saavedra, and J. Bascompte (2014), science **345** (6195), 1253497.

Rosenbaum, B., and E. A. Fronhofer (2023), Ecosphere **14** (4), e4503.

Routh, E. J. (1877), *A treatise on the stability of a given state of motion: particularly steady motion. Being the essay to which the adams prize was adjudged in 1877, in the University of Cambridge* (Macmillan and Company).

Roxburgh, S. H., and J. B. Wilson (2000), Oikos **88** (2), 395.

Roy, F., M. Barbier, G. Biroli, and G. Bunin (2020), PLoS computational biology **16** (5), e1007827.

Saavedra, S., R. P. Rohr, J. Bascompte, O. Godoy, N. J. Kraft, and J. M. Levine (2017), Ecological Monographs **87** (3), 470.

Schmitz, D. A., T. Wechsler, I. Mignot, and R. Kümmerli (2024), ISME communications **4** (1), ycae045.

Serván, C. A., J. A. Capitán, J. Grilli, K. E. Morrison, and S. Allesina (2018), Nature ecology & evolution **2** (8), 1237.

Sinervo, B., and C. M. Lively (1996), Nature **380** (6571), 240.

Solé, R., V. Maull, D. R. Amor, J. P. Mauri, and C.-P. Núria (2024), ACS Synthetic Biology.

Soliveres, S., F. T. Maestre, W. Ulrich, P. Manning, S. Boch, M. A. Bowker, D. Prati, M. Delgado-Baquerizo, J. L. Quero, I. Schöning, *et al.* (2015), Ecology letters **18** (8), 790.

Strauss, S. Y. (1991), Trends in Ecology & Evolution **6** (7), 206.

Strogatz, S. H. (2018), *Nonlinear dynamics and chaos with student solutions manual: With applications to physics, biology, chemistry, and engineering* (CRC press).

Suweis, S., F. Simini, J. R. Banavar, and A. Maritan (2013), Nature **500** (7463), 449.

Szathmáry, E., and I. Gladkih (1989), Journal of Theoretical Biology **138** (1), 55.

Szathmáry, E., and J. M. Smith (1997), Journal of theoretical biology **187** (4), 555.

Tilman, D., C. L. Lehman, and C. E. Bristow (1998), The American Naturalist **151** (3), 277.

Toni, B. (2014), in *New Frontiers of Multidisciplinary Research in STEAM-H (Science, Technology, Engineering, Agriculture, Mathematics, and Health)* (Springer) pp. 205–240.

Trefethen, L. N., and D. Bau (2022), *Numerical linear algebra* (SIAM).

Valverde, S., J. Piñero, B. Corominas-Murtra, J. Montoya, L. Joppa, and R. Solé (2018), Nature ecology & evolution **2** (1), 94.

Van Der Hofstad, R. (2024), *Random graphs and complex networks*, Vol. 2 (Cambridge university press).

Vandermeer, J. (1980), The American Naturalist **116** (3), 441.

Venturelli, O. S., A. V. Carr, G. Fisher, R. H. Hsu, R. Lau, B. P. Bowen, S. Hromada, T. Northen, and A. P. Arkin (2018), Molecular systems biology **14** (6), e8157.

Wootton, J. T. (1994), Annual review of ecology and systematics , 443.

Wright, E. S., and K. H. Vetsigian (2016), Nature communications **7** (1), 11274.

Yodzis, P. (1988), Ecology **69** (2), 508.

Yu, X., M. F. Polz, and E. J. Alm (2019), The ISME journal **13** (6), 1602.

Zelnik, Y. R., N. Galiana, M. Barbier, M. Loreau, E. Galbraith, and J.-F. Arnoldi (2024), Ecology Letters **27** (1), e14358.

LEGEND OF FIGURES

1. **Comparing the EC and multistability boundaries in random uncorrelated matrices.** Multistability boundaries shift with diversity, whereas EC boundaries sharpen but remain fixed; EC can precede multistability only for very small communities.

2. **Probability of observing certain motifs in a random matrix.** Coexisting pairs and RPS triplets appear sharply as σ increases, matching analytical coexistence thresholds.

3. **Emergent coexistence under different matrix randomizations.** EC is common across randomization types except in fully symmetric interactions, where EC is rare.

4. **Emergent coexistence in different dynamical models.** Extended caption in Section II.A.5.

5. **Fraction of surviving species and EC under decreasing connectivity.** Lower connectivity increases coexistence and the likelihood that stable states exhibit EC.

6. **Fraction of excluding pairs under decreasing connectivity and hierarchical interactions.** Connectivity and hierarchy shape EC patterns; analytical boundaries still capture observed communities.

7. **Row sums of coexisting species interaction matrices are homogeneous.** Feasible communities show more homogeneous row sums than randomized expectations.

8. **Fraction of positive net effects.** Before EC appears, nearly half of net effects become positive; facilitation dominates early.

9. **Correlation between direct and net effects.** Direct and net effects correlate only in weak-interaction communities; EC states show decorrelation or anticorrelation.

10. **Numerical and analytical predictions for the condition number κ.** Strong interactions increase the condition number far beyond RMT expectations, reducing predictability.

11. **Testing the role of ϕ and κ in community assembly.** Pairwise assembly fails as collectivity increases; measurement errors propagate strongly when κ is large.

12. **Stability predictions for EC states.** RMT thresholds underpredict stability; RH criteria correctly capture stability even under strong competition.

13. **Random matrix theory and Routh-Hurwitz predictions on stability.** Communities can violate RMT thresholds yet satisfy RH, explaining stability under strong competition.

14. **Low rank exclusions require at least four exclusionary interactions.** LRE motifs require a minimum structural complexity; they are rare in small systems.

15. **Low rank exclusions with and without the 4-exclusions requirement.** Accounting for the 4-interaction minimum clarifies why LRE are extremely rare in large communities.

16. **Laird-Schamp index with and without $s_{\min}$.** A modified index resolves degeneracies and aligns with other intransitivity measures: intransitivity vanishes with diversity.

17. **Fraction of coexisting and stable+coexisting states under increasing σ_r.** Growth-rate heterogeneity leaves coexistence unchanged but decreases stability by increasing Jacobian heterogeneity.

18. **Fraction of communities with EC and fraction of excluding pairs.** Heterogeneous growth rates preserve qualitative EC patterns across parameter space.
